# Noninvasive Biological Samples to Detect and Diagnose Infections due to Trypanosomatidae Parasites: A Systematic Review and Meta-Analysis

**DOI:** 10.3390/ijms21051684

**Published:** 2020-02-29

**Authors:** Denis Sereno, Mohammad Akhoundi, Kourosh Sayehmri, Asad Mirzaei, Philippe Holzmuller, Veerle Lejon, Etienne Waleckx

**Affiliations:** 1 Institut de Recherche pour le Dévelopement, Université de Montpellier, UMR INTERTRYP IRD, CIRAD, 34032 Montpellier, France; veerle.lejon@ird.fr (V.L.); etienne.waleckx@ird.fr (E.W.); 2 Institut de Recherche pour le Dévelopement, Université de Montpellier, UMR MIVEGEC IRD, CNRS, 34032 Montpellier, France; 3 Parasitology-Mycology Department, Avicenne Hospital, AP-HP, 93000 Bobigny, France; m.akhoundi@yahoo.com; 4 Psychosocial Injuries Research Center, Department of Biostatistics, Ilam University of Medical Sciences, Ilam 6931851147, Iran; sayehmiri@razi.tums.ac.ir; 5 Parasitology Department, Paramedical School, Ilam University of Medical Sciences, Ilam 6931851147, Iran; amirzaeii@yahoo.com; 6 Zoonotic Diseases Research Center, Ilam University of Medical Sciences, Ilam 6931851147, Iran; 7 CIRAD, UMR ASTRE “Animal, Santé, Territoires, Risques et Ecosystèmes”, F-34398 Montpellier, France; philippe.holzmuller@cirad.fr; 8 ASTRE, CIRAD, INRAE, Université de Montpellier (I-MUSE), 34000 Montpellier, France; 9 Centro de Investigaciones Regionales «Dr Hideyo Noguchi», Universidad autònoma de yucatán, Merida, Yucatán 97000, Mexico

**Keywords:** leishmaniases, chagas disease, human African trypanosomiasis, animal trypanosomiasis, diagnosis, non-invasive, meta-analysis, vector-borne diseases, neglected tropical disease

## Abstract

Unicellular eukaryotes of the Trypanosomatidae family include human and animal pathogens that belong to the *Trypanosoma* and *Leishmania* genera. Diagnosis of the diseases they cause requires the sampling of body fluids (e.g., blood, lymph, peritoneal fluid, cerebrospinal fluid) or organ biopsies (e.g., bone marrow, spleen), which are mostly obtained through invasive methods. Body fluids or appendages can be alternatives to these invasive biopsies but appropriateness remains poorly studied. To further address this question, we perform a systematic review on clues evidencing the presence of parasites, genetic material, antibodies, and antigens in body secretions, appendages, or the organs or proximal tissues that produce these materials. Paper selection was based on searches in PubMed, Web of Science, WorldWideScience, SciELO, Embase, and Google. The information of each selected article (*n* = 333) was classified into different sections and data were extracted from 77 papers. The presence of Trypanosomatidae parasites has been tracked in most of organs or proximal tissues that produce body secretions or appendages, in naturally or experimentally infected hosts. The meta-analysis highlights the paucity of studies on human African trypanosomiasis and an absence on animal trypanosomiasis. Among the collected data high heterogeneity in terms of the I^2^ statistic (100%) is recorded. A high positivity is recorded for antibody and genetic material detection in urine of patients and dogs suffering leishmaniasis, and of antigens for leishmaniasis and Chagas disease. Data on conjunctival swabs can be analyzed with molecular methods solely for dogs suffering canine visceral leishmaniasis. Saliva and hair/bristles showed a pretty good positivity that support their potential to be used for leishmaniasis diagnosis. In conclusion, our study pinpoints significant gaps that need to be filled in order to properly address the interest of body secretion and hair or bristles for the diagnosis of infections caused by Leishmania and by other Trypanosomatidae parasites.

## 1. Introduction

**Figure 1:**
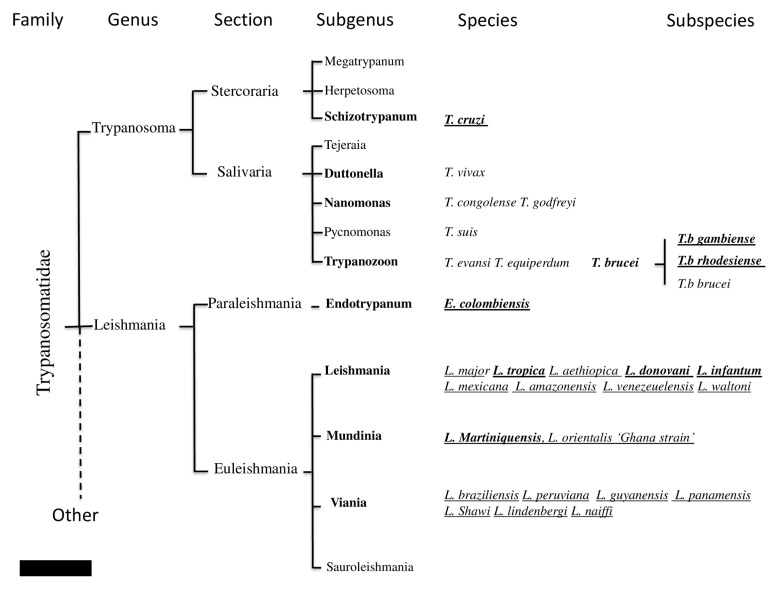
Classification of human and animal pathogenic trypanosomatids. Human pathogenic species are underlined, and pathogens causing systemic infection are in bold.

Unicellular eukaryotes of the Trypanosomatidae family include human and animal pathogens that belong to the *Trypanosoma* and *Leishmania* genera (including *Endotrypanum*) (Figure 1). *Leishmania* and possibly *Trypanosoma* are probably descended from the parasites of blood-sucking insects that survived accidental transmission to a vertebrate host during feeding [1]. They possess a complex life cycle that includes arthropod vectors belonging to the Hemiptera and Diptera orders. Two *Trypanosoma* subspecies of *T. brucei* (i.e., *Trypanosoma brucei gambiense*, *T. brucei rhodesiense*) and *T. cruzi*, along with 21 species of *Leishmania*, are pathogenic for humans. They cause human African trypanosomiasis (HAT or sleeping sickness), Chagas disease (CD), and cutaneous (CL), muco-cutaneous (MCL), or visceral (VL) leishmaniases [2,3,4,5]. Occasional infections in humans with *T. evansi*, *T lewisi*, *T. brucei brucei*, or *T. congolense* have been described, but little is known about the public health importance of these diseases [6]. In addition to their impact on human health, these diseases also affect domestic, feral, or wild animals. Canine visceral leishmaniases (CVL) are mainly caused by *L. infantum* infection and occasionally by *L. donovani* or *L. major*. *Trypanosoma congolense*, *T. evansi*, *T. b. brucei*, *T. vivax*, *T. simiae*, *T. suis*, and more rarely, *T. godfreyi*, affect livestock, causing animal trypanosomiasis, and *T. equiperdum* affects equids [7,8] (Figure 1). Altogether, more than 30 million people are infected with these pathogens, and approximately 100,000 persons die every year from *Trypanosoma brucei* spp., *T. cruzi*, or *Leishmania* spp. infections [9]. An estimated 48 million cattle are at risk of contracting animal trypanosomiasis in Africa. African animal trypanosomiasis (AAT) causes about 3 million deaths in cattle every year (http://www.fao.org/paat/the-programme/the-disease/en/).

Leishmaniases rank after malaria in terms of annual incidence and affect 98 countries and territories worldwide. Visceral leishmaniasis kills between 20,000 and 30,000 persons annually; 1 million cutaneous leishmaniasis cases have been reported over the past five years, and over 1 billion people live at risk of infection (http://www.who.int/leishmaniasis/en/). *Leishmania* spp. are obligate intracellular protozoan parasites transmitted mainly by two genera of sandflies, namely, *Phlebotomus* and *Lutzomyia* [4]. Sexual transmission of *Leishmania* species responsible for canine and human visceral leishmaniases is documented [10,11,12,13], as well as blood transmission in dogs and transmission between drug users through contaminated needles [14,15]. Congenital transmission was first described in 1926 and is more frequently reported today [16,17]]. Following *Leishmania* infection, metacyclic promastigotes are rapidly engulfed (macrophages and dendritic cells) and then disseminate from the skin to the spleen, liver, and bone marrow myeloid cells [18]. *Leishmania* causes cutaneous or visceral afflictions. Cutaneous lesions vary in their severity (e.g., lesion size), numbers, clinical appearance (e.g., dry or wet lesion) and incubation time (e.g., the time for spontaneous cure) [19]. Nevertheless, at least in an experimental model of infection, some *Leishmania* species responsible for cutaneous forms have the capacity to disseminate into internal organs [20]. Most of the patients infected with *L. donovani* and *L. infantum* develop only subclinical disease or chronic latent infections without any clinical manifestation [21,22]. In patients, irregular fever, splenomegaly, pancytopenia, hepatosplenomegaly, and hypergammaglobulinemia characterize visceral leishmaniasis. Atypical disseminated leishmaniasis might be observed in Leishmania–HIV coinfected persons, with parasites colonizing the gastrointestinal mucosa, the respiratory tract, and the liver [19]. In addition to human diseases, leishmaniasis affects dogs, where it provokes a deadly disease if not treated. Lymphadenomegaly, a loss of body condition, pale mucous membranes, splenomegaly, alopecia, furfur, and onychogryphosis are the most frequently observed clinical signs, but many other clinical features, alone or combined, such as polyuria/polydipsia, diarrhea, fever, arthropathy, or ocular lesions, can be present [23,24,25]. 

Chagas disease, also known as American trypanosomiasis, is caused by *T. cruzi* and has been reported in all Latin American countries, where it constitutes the most important parasitic infection and has emerged as a disease of importance outside of endemic areas, largely as a result of migration [26,27]. Six to seven million people are estimated to be infected with the parasite, but the disease burden may be underestimated, as evidenced at least in Mexico [28]. *Trypanosoma cruzi* is mainly transmitted to humans and other mammals by hematophagous insects called triaomines or kissing bugs belonging to the subfamily Triatominae [27]. Nevertheless, the parasite can also be transmitted via non-vectorial routes, such as blood transfusion, congenital transmission, organ transplantation, ingestion of food and beverages contaminated with *T. cruzi* (oral transmission; a typical example is the ingestion of fruit juice contaminated by triaomine feces, frequently reported in the Amazonian region [29], or laboratory accidents. Sexual transmission was also recently documented in a murine model, but there is no evidence of such a mechanism of transmission in humans [30]. Inside its host, *T. cruzi* is internalized in the cells of the immune system. Once an individual acquires the parasite, the infection develops progressively. Chagas disease has two clinical phases. The first, called acute phase, is characterized by a high parasitaemia in patients’ blood. During this phase, the parasite undergoes multiplication and infects local macrophages, fibroblasts, and muscle cells. During this phase, the patients have generally no or only mild nonspecific symptoms (e.g., fever). Nevertheless, some acute cases (2–6%) can lead to death due to myocarditis and meningoencephalitis, mostly in children. Accute cases can also be observed following organ transplantation [31] or HIV infection [32]. The second phase of the infection is known as chronic phase. At the beginning of this phase, the parasites remain hidden in the body, and there are no clinical or physical signs. However, over the years, 30–40% of patients develop clinical symptoms. Chronic chagasic cardiomyopathy is the most frequent and serious clinical manifestation leading to heart failure or sudden death [33]. Other patients present digestive (megaesophagus, megacolon among others) or neurological alterations or a combination of them [33,34]. Urinary tract disorder and dysfunction is documented in patients with cardiac and digestive Chagas Disease [35].There is evidence of functional and structural kidney abnormalities after *T. cruzi* infection, associated with reduction in renal blood flow, proximal tubular damage, and inflammatory interstitial infiltrate [36,37]. In immune compromised patients cutaneous lesions caused by reactivation of *T. cruzi* is also described [38].

Sleeping sickness is caused by trypanosomes transmitted by tsetse flies (*Glossina* spp.). The overall number of infected people is approximately 10,000 [39]. The disease presents two distinct forms, chronic and acute, which are caused by two distinct trypanosome subspecies, transmitted by two distinct vector species. The chronic form, caused by *T. brucei gambiense*, is distributed in western and central Africa and is transmitted mainly by *Glossina palpalpalis* sp., while the acute form, caused by *T. brucei rhodesiense*, is distributed in east Africa and is transmitted mainly by *Glossina morsitans* species. Bites of infected tsetse flies and the injection of salivarian trypanosomes often result in the formation of skin ulceration (chancre). During the first stage of the disease (stage 1, known as the hematolymphatic stage), the trypanosomes are present and multiply in the blood and in the lymph nodes. Stage 2, the meningo-encephalitic stage, begins after the invasion of the central nervous system (CNS) [40,41]. An early symptom of *T. b. gambiense* infection is hypertrophy of the lymph nodes, which corresponds to a multiplication of parasites within the lymphatic system. Parasites can be seen in the lymph nodes of the cervical chain after a puncture of the ganglion. During this invasive phase of the disease, lesions are observed in the liver, the spleen, the cardiovascular and endocrine system, as well as the eyes [42,43]. Heart abnormality cases are scarce, but in cases of occurrence, they can provoke acute and fatal cardiac damage by arrhythmia, mainly in *T. b. rhodesiense*-infected patients. *T. b. gambiense* infection can cause persistent and dissociated tachycardia and atrioventricular rhythm or signs in favor of early myocarditis or pericarditis [44,45,46,47,48]. In addition, descriptions of cutaneous symptoms associated with African trypanosomiasis and distinct “trypanid” skin lesions are documented [49,50,51]. The ability of *Trypanosoma brucei* to survive at the epidermis interface is reported in experimental infection models [52,53]. They have also been observed in slides of historical skin biopsies taken as a part of a diagnostic screening program for *Onchocerca microfilariae* in the trypanosomiasis-endemic region of the Democratic Republic of the Congo [52].

In addition to human African trypanosomiasis, animal trypanosomiasis (AT; nagana, surra, dourine) affects livestock (cattle, small ruminants, and pigs), equids (horses, donkeys) and camelids (camels, dromedaries, llamas, and alpacas). They give rise to important economic losses in Africa, the Middle East, Asia, and Latin America [7,54]. In addition to their cyclical transmission by tsetse flies (*Glossina* sp.), trypanosomes infecting animals can also be transmitted mechanically by blood-sucking insects (mainly stomoxines and tabanids) and even directly via venereal transmission in the case of dourine [54,55,56]. In cattle and small ruminants, most cases are caused by *Trypanosoma congolense* and *T. vivax*, but infection by a variety of other trypanosomes and mixed infections are also frequent. *Trypanosoma evansi* affects horses and camels and can affect other mammals [56]. *Trypanosoma brucei* s. l. might have a minor role in the pathogenesis of cattle even if it is often isolated in the blood of infected animals. *Trypanosoma godfreyi* is rare and was first described in the midgut of tsetse flies caught in Gambia [57]. *Trypanosoma simiae* and, rarely, *T. suis* have been identified in livestock, but their precise role in the disease remains unclear [58]. *Trypanosoma equiperdum* is venereally transmitted and appears to be restricted to horses and donkeys. *Trypanosoma evansi* is the trypanosome species that presents both the widest variety of described hosts, including rodents, ruminants, equids, camelids, and humans [56], and the largest diversity in transmission modes, including cyclical via tsetse flies, mechanical via blood-sucking flies and vampire bats, possibly sexual, and via contaminated meat or carcasses [54,55,56]. Under experimental conditions, *T. congolense* can be mechanically transmitted by biting flies (e.g., tabanids and stomoxines) [59], the epidemiological consequence of which in nature is not clear, and currently, only *T. evansi* and *T. vivax* have adapted to mechanical transmission and spread beyond the tsetse transmission zone in livestock industries of Asia (*T. evansi*) and South America (*T. vivax*, *T. evansi*) [60]. Overall, African animal trypanosomiasis remains one of the most important infectious disease constraints to livestock production in sub-Saharan Africa. The hallmarks of clinical signs reminiscent of animal trypanosomiasis infection include ventral edema, emaciation, anemia, and neurological symptoms [61,62], but there is no dichotomy in the clinical evolution as for human infections, probably because trypanosomes can be found in all body fluids of animals (blood, lymph, CSF, urine, aqueous humor, synovium). Overall, animal trypanosomiases remain among the most important infectious disease constraints to livestock production in sub-Saharan Africa, as well as Latin America and Asia.

The methodologies used for typing trypanosomatidae parasites and diagnosing infections are summarized in Table 1. Microscopic examination of biopsy represents the simplest methodological approach to diagnose infection and detect pathogens. The hard identification at the species/subspecies level and its low sensitivity are limitations. Like microscopic examination, the in vitro parasite cultivation presents the advantage of being relatively simple to perform but has low sensitivity and requires sophisticated laboratory equipment. Xenodiagnosis is more complex than the other parasitological approaches but does not require biological sampling. Molecular methods involved polymerase chain reaction (PCR) or isothermal amplification of the genetic material. PCR is relatively simple to perform and to visualize. Refinements in PCR technologies included the development of nested PCR and of multiplexed PCR methodologies that have increased sensitivity and discriminative capacity of the test. Other refinements in the detection of the amplified product include PCR–ELISA (enzyme-linked immunosorbent assay). PCR–RFLP (restriction fragment length polymorphism) allows the detection of the variation between DNA fragments patterns, generated by restriction enzyme digestion caused by alternative nucleotides at the restriction sites that can be used for *Leishmania* and trypanosome species discrimination. PCR–HRM (high resolution melting) detects double-stranded DNA (dsDNA) alternatives by ascertaining changes in the fluorescence intensity, of a DNA-intercalating dye, during the dissociation process of dsDNA to single-stranded DNA (ssDNA). It was applied with success to *Leishmania* and *T. cruzi* detection and species and Discrete Typing Unit (DTU) delineation [63,64]. Oligochromatography-PCR (OC–PCR) provides a simple and rapid format for the detection of PCR or nucleic acid sequence-based amplification (NASBA) products, visualized on a dipstick by hybridization with a gold-conjugated probe. This detection format takes only 5–10 min and requires no equipment other than a water bath and a pipette [65,66]. Loop-mediated isothermal amplification (LAMP) uses the strand displacement activity of a DNA polymerase to amplify the dsDNA target with four primers designed to recognize six distinct regions. Amplification is completed in a single step at an isothermal temperature [67]. LAMP can be more sensitive than conventional PCR for the detection of *Leishmania* and *Trypanosoma* species [68,69]. The dermal diagnostic tests or Leishmanin skin test (LST)/Montenegro test is based on the delayed-type hypersensitivity (DTH) reactions raised following intradermal injection of killed *Leishmania* promastigotes into the skin forearm. It does not require biological sampling. Indirect immunofluorescence (IFAT) relies on the use parasites layered on a fluorescent glass slide that is used to test the presence of anti-parasites antibodies in the patient serum. This methodology was assayed for the serodiagnosis of Chagas disease, sleeping sickness, leishmaniasis [70,71,72], and animal trypanosomiasis [73]. IFAT methodology is more used for surveillance programs than for clinical diagnosis. Western blot allows us to visualize antigens targeted during antibody response. It presents the advantage of being more sensitive and specific than ELISA (see below). The direct agglutination test (DAT), further modified for detection of the agglutination activity on a card (CATT), allows the visualization of the precipitin activity. It uses whole micro-organisms as a means of looking for serum antibodies. CATT is a commonly serological test for HAT and is still in use for AAT serodiagnosis [74,75]. The agglutination methodology can also be performed with antibody-coated latex beads to trap antigen. KAtex, a commercialized latex agglutination test, is developed for the diagnosis of visceral leishmaniasis and uses a specific *Leishmania* antibody coated on latex particles [76]. Enzyme-linked immunoabsorbent assays (ELISA) can be performed to detect and quantify antibodies or antigens in samples. Alternatively, sandwich ELISA can be used to detect the circulating parasite’s antigens, which informs on the ongoing infectious process. Immunochromatography (ICT) or lateral flow test is based on a series of capillary beds that have the capacity to transport fluid spontaneously. The analyte is deposited on the dipstick and then spontaneously migrates to the first element that acts as a sponge to holds an excess of sample fluid. Once soaked, the fluid migrates to the second element in which the antibody or antigen is present in conjunction with colored particles. The analyte migrates to the third component of the test, in which antibodies are immobilized to stop the flow.

Hematogenous dissemination and tissue tropism are part of the infectious process of trypanosomatid pathogens. During the blood-feeding process, trypanosomatid pathogens are injected (*Leishmania*, salivarian trypanosomes) or deposited (stercorarian trypanosomes) on the skin of the host. Following their introduction into the bloodstream, they disseminate into specific organs or tissues and multiply. In these infections, as in a majority of other infections, pathology often correlates with the sites of accumulation of the infectious agent, but variations in disease outcomes and presentations are also related to the interaction between host and parasite [113,114,115,116]. The selection of the appropriate biopsy site for diagnoses relates to the physiopathology of the diseases, reflecting the disseminative capacity (tissue or organ tropism) of these pathogens within its host. Therefore, the diagnosis of these diseases requires the sampling of body fluids (e.g., blood, lymph, peritoneal fluid, cerebrospinal fluid) or organ biopsies (e.g., bone marrow, spleen), which are mostly obtained through invasive methods. Alternative biological samples, such as body secretions (e.g., milk, saliva, urine, semen, nasal secretion, lacrimal fluid, earwax, sweat, feces) or appendages (e.g., nail, hair, bristles) that are constantly produced might be an interesting alternative to invasive biopsies. Non-invasive biological sampling that does not require trained professionals and are easy and safe to collect would render the diagnosis more convenient. We address the interest of such biological material via a systematic review of the published literature and meta-analysis on data extracted from a defined pool of published papers. 

## 2. Results and Discussion

### 2.1. Study Selection

**Figure 2:**
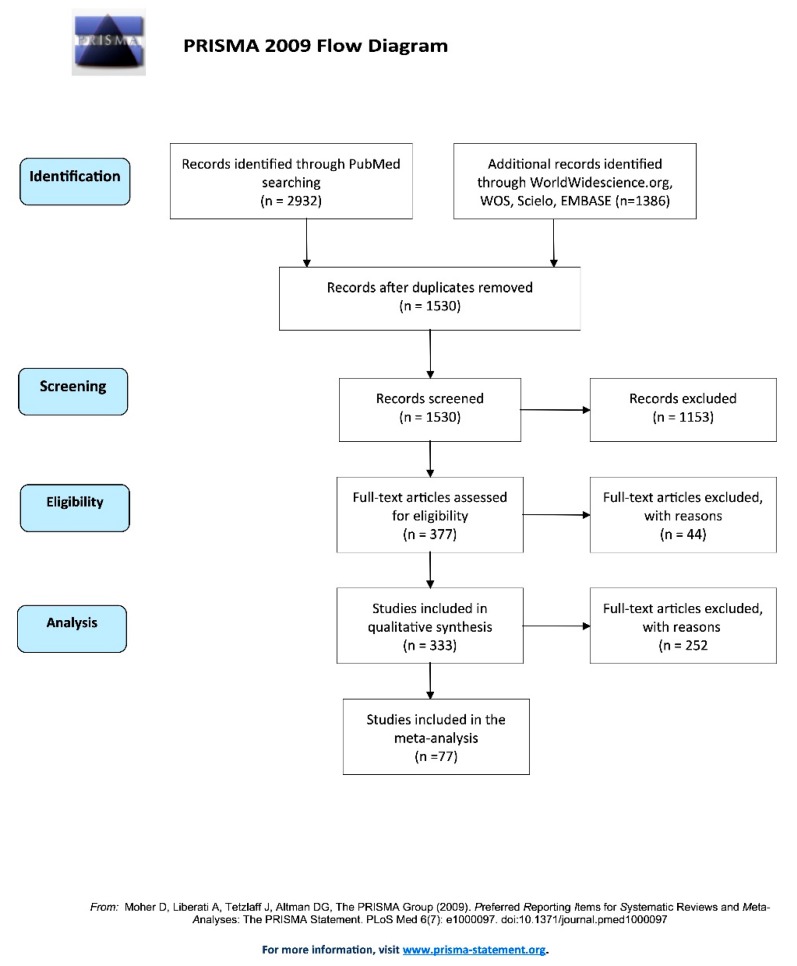
PRISMA flowchart of the systematic review and meta-analysis.

In the primary search, 2932 documents were identified via PubMed, and additional screening with other databases allowed the selection of another 1386 documents. After duplicate removal, 1530 documents were screened for relevance according to their titles and abstracts; at this stage, 1153 documents were omitted. The full text of the 377 studies was carefully read, and the ineligible (*n* = 44) documents were omitted. The 333 remaining manuscripts were included in the systematic review. Data could be extracted from 77 articles. A diagram of the study plan, following the PRISMA statement, is given in Figure 2.

### 2.2. Systematic Review of Non-Invasive Sampling Strategies for the Diagnosis and Detection of Trypanosomatid Pathogens and Infections

#### 2.2.1. Urine

Urine is an easy-to-collect secretion that is produced daily. Therefore, a large amount of information has been gathered on the presence of trypanosomatid parasites within this liquid.

Human and animal leishmaniases. The survival capacity of *Leishmania,* and *T. cruzi* in urine has been known since 1966 [117]. *In vitro,* urine can promote the growth of the *Leishmania* promastigote and can be used as a low-cost culture adjuvant alternative to serum [118]. The first evidence of the presence of *Leishmania* in the urine of patients infected by *L. donovani* came in the 1930s through the detection of Leishman–Donovan bodies in the urine of infected patients [119]. The presence of viable *Leishmania* parasites in the urine of infected individuals is documented [120,121,122]. The crossing of the glomerular barrier by *Leishmania* is thought to be a consequence of VL renal lesions and renal failure [123,124]. Tubulointerstitial involvement and glomerulonephritis are the main causative agents of the proteinuria disorder, which is common in most patients with a clinical episode of leishmaniasis [125,126,127]. In infected individuals, urine represents a fluid from which parasite DNA is easily extracted for detection and species identification [128], which has been probed in the urine of patients [109,121,129,130] and in animal reservoirs [131,132]. These searches were performed in VL caused by *L. infantum* [109,120,121,133]; in CL and VL-HIV+ patients infected by *L. martiniquensis* [134]; in CL due to *L. major* or *L. tropica* [109]; in South American cutaneous and mucocutaneous leishmaniasis caused by *L. braziliensis*, *L. guyanensis*, or *L. peruviana* [130]; and in canine visceral leishmaniasis [131,132,135]. The presence, in urine, of precipitin activities directed against several microorganisms has been known since 1948 [136]. The nature of these activities was formerly attributed to antibodies in 1965 [137]. In 1983, the presence of anti-*Leishmania* antibodies in urine was demonstrated [138,139]. Since then, the anti-*Leishmania* antibody response in patient urine to diagnose VL has been further investigated. ELISA, which uses recombinant antigens or whole antigen preparations as well as the direct agglutination test (DAT), has been used to test for disease diagnosis using patient urine [105,139,140,141,142,143,144,145,146,147]. Immunochromatographic tests to detect rk39 antibodies are currently commercialized and have been thoroughly tested in urine [105,144,148,149,150]. Antibodies present in urine directed against rKP42, a kinesin-related protein and a homolog of rK39, also showed remarkable sensitivity and specificity for VL [151]. This specificity and sensitivity were comparable to those obtained with ELISA performed using acetone-treated *L. donovani* promastigote antigens or DAT [139,140]. The detection of the antibody response against *Leishmania* infection, due to *L. major*, *L. tropica*, or *L. infantum*, was also investigated using Western blot [109,152]. IgA or IgG are detected in the urine of dogs suffering from leishmaniasis [153], where antibodies directed against *L. infantum* are present [154,155,156]. Because of the persistence of antibodies after cure, these tests cannot be used to diagnose VL in people with a past history of VL. A search for *Leishmania* proteins in urine has therefore been undertaken [138,152,157,158,159], as well as for changes in the urinary proteome of infected individuals [160]. In *L. infantum*-infected patients, iron superoxide dismutase, *L. infantum* tryparedoxin, and *L. infantum* nuclear transport factor 2 (Li-ntf2) were identified by mass spectrometry analysis [158,159]. When used in a multiplex ELISA test, these biomarkers show a sensitivity superior to 80% for VL diagnosis caused by *L. infantum* but fail to accurately diagnose VL due to *L. donovani* [158,159]. In *L. donovani*-infected patients, two biomarkers showing a sensitivity of approximately 82% were characterized [161]. A low-molecular-mass heat-stable leishmanial carbohydrate antigen [162] has allowed the development of a latex agglutination test (KAtex) to be commercialized [76,162] and compared to ELISA [163]. Its efficiency was thoroughly tested in various VL endemic areas [76,105,129,148,164,165,166,167,168,169]. The KAtex test in urine might be useful for the detection of VL within the clinical case definition: fever for more than two weeks, splenomegaly, and no previous history of VL [170]. As a simple field-deployable *Leishmania* urine antigen test, the capacity of this test to predict initial treatment failure and relapse was investigated. Overall, this preliminary study showed that the test may be used for risk stratification of initial treatment failure and VL relapse in HIV patients [171]. A meta-analysis of these various tests has been recently performed [170,172]. Overall, these meta-analyses point out that the rK39 assay provides the highest sensitivity and that the ELISA has the highest specificity for the diagnosis of VL [172].

Human African trypanosomiasis and animal trypanosomiases. No information on the presence of DNA, antibodies, or antigens in the urine of human individuals affected by sleeping sickness was collected during the systematic review. The sole evidence on the presence of genetic material in urine comes from an experimental infection of vervet monkeys by *T. brucei.* In this model, *Trypanosoma* DNA could be amplified from urine, with LAMP as early as 17 days postinfection [173]. Biochemical changes associated with trypanosome infection have been published on animal models infected by various *Trypanosoma* species. Rabbits infected by parasites of the *T. brucei* subgroup showed a progressive increase in proteins released in the urine [174]. The presence of fibrinogen and fibrin degradation products in the urine of rabbits infected by *T. brucei* is suggestive of a glomerular permeability change [175]. Mice or *Microtus montanus* infected by *T. b. gambiense* showed an increase in the excretion of aromatic amino acid catabolites [176,177,178,179]. In mice infected with *T. evansi,* the concentration in phenylpyruvic acid, 4-hydroxyphenylpyruvic acid, and indole-3-pyruvic acid correlates with parasitemia and returns to normal following suramin treatment [180]. These metabolites were also detected in dogs and donkeys experimentally infected [180]. The high rate of aromatic amino acid catabolism by African trypanosomes was associated with the large decrease in free serum levels of aromatic amino acids and with alterations in host tyrosine and phenylalanine metabolism. These events correlate with the pathology of sleeping sickness and the depletion reported in certain amino acids (tryptophan), which would lead to the depletion of essential metabolites such as serotonin and the toxicity of end products such as phenylpyruvate, reviewed in [181]. Changes in the urinary proteome of patients suffering from sleeping sickness were observed, notably, in proteins related to several infectious processes. These changes can be the rationale for developing non-invasive tools aimed at tracking the disease stage [182]. In animal models, *T. brucei* parasites were observed in the kidney glomeruli of infected rats, and *T. lewisi* in the kidney capillaries [183,184]. *T. musculi,* a parasite specific to mice, resides in the blood and lacks intracellular stages. After immune clearance of the flagellates from the general circulation, mice became resistant to reinfection. However, long after parasites were no longer detected in the peripheral blood, they still persisted in the vasa recta of the kidneys in a peculiar biological stage [185], releasing molecular determinants in the urine as potential diagnostic biomarkers.

Chagas Disease. Evidence on the capacity of *T. cruzi* to survive in urine came along with those on *Leishmania* [117], and *T. cruzi* amastigotes have been occasionally detected in the kidney [186]. Parasite DNA was detected in the urine of experimentally infected pigs (*Sus scrofa*) or mice [187,188,189]. The crossing of *T. cruzi* to urine in experimentally infected mice is apparently independent of renal injuries [189]. The presence of DNA in urine is associated with the presence of parasite DNA in blood and heart and with a high level of parasite DNA in blood, but not with the presence of parasites in kidney or kidney injury [189,190,191]. The detection of antigens within the urine of patients suffering from acute [192] or chronic CD [193] has opened up some new innovative approaches for diagnosis. A number of *T. cruzi* urinary antigens can be identified and classified according to their molecular weight, such as the 80 kDa iron-binding protein or the 150–160 kDa antigen. These antigens were detected by the use of antibodies raised against an immunodominant epitope of *T. cruzi*. In addition, parasite tubulin was also detected in urine as well as a set of immunoreactive antigens [192,194,195,196]. In mice, cruzipain, a major cysteine protease of *T. cruzi,* was detected in urine [187]. To develop a diagnostic test based on a capture ELISA system, a panel of polyclonal antibodies was produced against membrane antigens or trypomastigote excreted/secreted antigens. The test performed on urine from patients positive for ELISA capture against sera demonstrated a 100% positivity [197]. Antigens are present in urine at low concentrations and are susceptible to degradation after collection. These characteristics limit the sensitivity and reliability of all urinary-based antigen detection. The use of nanoporous hydrogel particles produced with poly(N-isopropylacrylamide) (poly(NIPAm)) and N,N9-methylenebisacrylamide (BAAm) coupled to chemical baits via amidation reaction has the potential to concentrate and preserve the antigens [198] for its application using urine [199]. The test, called Chunap (Chagas urine nanoparticle test), has been further developed and evaluated for congenital transmission of *T. cruzi* [200]. In this condition, it showed more than 90% sensitivity and more than 95% specificity [200]. It also demonstrated good sensitivity in HIV–*T. cruzi* coinfected cases [201].

#### 2.2.2. Feces

Human and Animal Leishmaniases. Most of the references that document the findings of *Leishmania* in human feces were published during the 1920s and l930s [202,203]. Anecdotally, descriptions of the presence of *L. tarentolae* in feces of the *Tarentola mauritanica* lizard were published approximately at the same time [204]. The detection of *Leishmania* amastigotes and its DNA in the feces of a dog infected by *L. infantum* was documented [205]. More recently, a screening of wild gorilla fecal samples revealed the presence of promastigotes and amastigotes of *L. major* within these samples [206]. Nevertheless, this finding has been a matter of debate [207,208]. More recently, a large diversity of trypanosomatid parasites in the feces of great apes, but no *Leishmania* DNA, was evidenced [209]. Since the 1920s, at the time Donovan bodies were detected in human feces, no additional information on the detection of parasites or the DNA of *Leishmania* in human feces has been published. The only other clues on the presence of *Leishmania* DNA in the human gut come from studies performed on pre-Columbian mummies using next-generation sequencing. These analyses highlight the presence of DNA related to *Leishmania* and *T. cruzi*, without being able to firmly identify *Leishmania* at the species level [210,211,212].

Human African Trypanosomiasis and Animal Trypanosomiases. The ITS1 region of *T. b. brucei, T. b. gambiense*, *T. b. rhodesiense*, and *T. b. evansi* was successfully amplified from DNA isolated from fecal samples of experimentally infected mice [213] and *T.* b. rhodesiense and/or *T.* b. gambiense DNA was detected in the feces of wild gorillas [209,213].

Chagas Disease. Megacolon is a pathological affliction that occurs in chagasic patients [34]. Evidence of the presence of *T. cruzi* DNA in the gut of pre-Columbian mummies is documented, depicting that the disease has a long evolutionary history with humans in South America [210,211,212,214,215]. The tissue tropism of various *T. cruzi* isolates was investigated in a mouse model of infection. In these experiments, parasite DNA was detected in the small intestine and rectum of the animals [216,217]. In infected mice, the gut is the primary site of parasite persistence in the BALB/c model of chronic Chagas disease and is associated with a perturbation in the gut microbiome [218,219]. In opossums (*Didelphis marsupialis*), one of the multiple wild reservoirs of *T. cruzi*, the developmental cycle that usually occurs in the intestine of the triatomine vector can take place in the anal odoriferous glands [220]. In human feces, to our knowledge, no information is currently published.

#### 2.2.3. Saliva/Oral Swab/Sputum

Oral swab, saliva, and sputum are the easiest and least-invasive sampling methodology for the detection of infectious pathogens. Although bronchoalveolar lavage is not considered a nonintrusive method to collect biological samples, it does not cause damage to tissues.

Human and animal leishmaniases. The presence of viable *Leishmania* parasites in the saliva of infected patients was demonstrated in 1934 by Forkner [221]. More recently, *L. braziliensis* was recovered from the saliva of a person suffering from cutaneous leishmaniasis [222]. A large number of studies describe the successful detection and identification of *Leishmania* DNA in saliva or oral swabs, with PCR or other methodologies of DNA amplification (LAMP). The DNA was amplified in *L. martiniquensis*-HIV positive and negative patients [134,223,224,225,226] but also in kala-azar patients infected by *L. donovani* [227,228] and in dogs suffering from CVL [229,230]. In 1994, a report discussed the presence of agglutinating anti-leishmania activity, an antibody, in the saliva of kala-azar patients [231]. The capacity of anti-leishmania antibodies present in the saliva to be used to diagnose CVL and VL was investigated more recently. For CVL, the detection of IgG2 and IgA antibodies targeting specific recombinant K39 protein (rK39) in saliva demonstrated the usefulness of this test to diagnose CVL and to differentiate between seropositive and seronegative dogs [232]. In humans, a preliminary experiment involving the detection of rK39 antibodies demonstrated 99.2% sensitivity and 100% specificity for *Leishmania* diagnosis using patient sputum [149]. Interestingly, KAtex showed a higher sensitivity to diagnose Mediterranean visceral leishmaniasis with oral fluid than with urine, even though this test was originally conceived to be used with urine [129]. In addition to saliva, *Leishmania* has been occasionally detected in the bronchoalveolar lavages of patients suffering from VL [233,234].

Human African Trypanosomiasis and Animal Trypanosomiases. Trypanosome-specific IgG can be detected in the saliva of *T. b. gambiense*-infected HAT patients using ELISA. Nevertheless, the antibody concentration is at least 250-fold lower in saliva than in serum [235]. The ELISA performed on the saliva of a cohort of 208 individuals, including 78 parasitologically confirmed patients, demonstrated a robust sensitivity and specificity (>90%) comparable with CATT performed on sera [236]. Since then, no additional experiments have been performed.

Chagas Disease. The first evidence on the presence of *T. cruzi* in the saliva of experimentally infected dogs dates from 1966 [117,237]. More recently, an ELISA that detected and quantified the IgG response to *T. cruzi* was developed using saliva from infected patients. The methodology was tested with success on saliva from patients with chronic infection, which is characterized by the absence of blood circulating parasites [238,239]. The oral swab was also tested to detect fragments of *Trypanosoma* DNA (*Trypanosoma dionisii*, *T. rangeli*, and *T. cruzi*) to evaluate the potential reservoirs for *T. cruzi* in gallery forest bats [240].

#### 2.2.4. Conjunctival Swab/Lacrimal Fluid/Occular

A swab is a small piece of soft material used for taking a small amount of substance from a body. The conjunctival or corneal swab, a routine practice to perform biological sampling to diagnose eye infection, has been applied to detect trypanosomatid pathogens.

Human and animal leishmaniases. In humans, ocular lesions are usually associated with systemic signs [241,242,243]. Ocular pathologies are documented in patients suffering from cutaneous [244,245,246,247,248,249], diffuse cutaneous [244,250] or post-kala-azar leishmaniasis [241], and in VL [251,252]. In dogs, keratoconjunctivitis and kerato-uveitis are described as the most usual symptoms, occurring in 16–80% of affected dogs [253,254]; keratoconjunctivitis is also observed in feline leishmaniasis [255]. *Leishmania* has been isolated from the aqueous humor of a patient suffering from leishmaniasis [242]. In addition, eyelid leishmaniasis is frequently described [247,248,249,256]. In naturally infected dogs, anti-*Leishmania* IgG was detected in the aqueous humor, although at a level not related to the serum level of IgG [257,258]. In dogs, histopathological investigations depicted the presence of plasmatic cells and macrophages bearing amastigote forms of *Leishmania*, in the ciliary body, sclerocorneal limbus, iris, and lacrimal duct, but also in smooth and striated muscles [257,259,260,261]. *Leishmania* were observed in squamous carcinoma cells from conjunctival swab samples from a HIV+ patient [262]. *Leishmania* DNA can be detected and quantified by qPCR in the lacrimal glands of symptomatic dogs [263]. All these clues have prompted testing the efficiency of the conjunctival swab for CVL diagnosis [264,265,266,267] and tracking asymptomatic dog infections [268] but also for diagnosing feline leishmaniasis [267,269,270]. In addition, the detection of *Leishmania* DNA in conjunctival swabs has also been applied to track *L. infantum* wild reservoirs [271,272].

Human African Trypanosomiasis and Animal Trypanosomiases. In humans, eye pathologies associated with trypanosome infections remain unusual [273], and an investigation for the presence of parasites, DNA, or antibodies within conjunctival swabs has not been performed. In dogs infected by *T. b. brucei*, the eyes are one of the most severely affected organs, and infection by *T. evansi* can provoke blindness [274,275]. Experimental infections of cats with *T. brucei* [276] and of cats and goats with *T. evansi* highlight their disseminative capacity in the eye, with their presence being detected in the aqueous humor [277,278].

Chagas Disease. In 1935, Romana first described the “unilateral schyzotrypanosomic conjunctivitis” associated with acute *T. cruzi* infection, later known as Romana’s sign [279]. The invasion of the human host by *T. cruzi* occurs in various ways but mainly via skin lesions or the conjunctival way [280,281]. *T. cruzi* parasites deposited on the conjunctiva, via the manipulation of contaminated bug feces, are drained with tears into the nasolacrimal duct and nasal cavity. Then, *T. cruzi* infects the most proximal tissues lined with cuboidal and columnar epithelial cells [281,282]. Surprisingly, reports on eye pathology in CD patients are very scarce. Recently, the first case of *Trypanosoma cruzi*–associated retinitis was diagnosed [283]. The presence of *T. cruzi* amastigotes in the conjunctiva, corneal stroma, the adjacent ocular muscle, and the interstitial macrophages of *Thrichomys apereoides* (Rodentia, Echimyidae) experimentally infected with *T. cruzi* has been documented [284].

#### 2.2.5. Genital Organs: Semen/Vulvular Secretion

Some trypanosomatid infections impact male and female reproductive organs, causing infertility [285]. *Leishmania* infection provokes a decrease in sperm quality, genital lesions, testicular amyloidosis, chronic prostatitis, and epididymal inflammation [285]. Chagas disease is associated with male hormonal changes and a loss in sperm quality due to parasitic load. In females, the invasion of the placenta and hormonal changes are associated with the overproduction of inflammatory cytokines in the oviduct and uterus. In sleeping sickness, an impairment in the spermatogenic cycle due to damage in the pituitary gland as well as damage to the reproductive organs is reported. In females, impairment in the estrus cycle due to pituitary gland damage is noticed [285].

Human and animal leishmaniases. Leishmaniasis does not belong to the broad list of potential sexually transmitted infections (STIs). Nevertheless, some evidence suggests that venereal transmission of leishmaniasis does occur in dogs and humans [10,286,287,288]. In humans, lesions in the male genitalia are well documented [289,290,291], with the presence of parasites [292,293]. In dogs, genital lesions associated with visceral leishmaniasis and the shedding of *Leishmania* sp. in the semen of naturally or experimentally infected dogs has been described and can lead to infertility [122,294,295]. In the prepuce and glans of male symptomatic dogs, heavy parasite burden has been detected and is associated with inflammation, testicular degeneration, atrophy, an absence of spermatogenesis, and necrosis [296]. In these dogs, immunohistochromatography showed that 75% of symptomatic dogs and 35% of asymptomatic dogs were positive for *Leishmania* in the testis. These percentages rose to 95% and 60% for symptomatic and asymptomatic leishmaniasis, respectively, in the epididymal duct. The detection of *Leishmania* parasites in semen has been evidenced through parasite culture [108], microscopic observation or immunohistology [294,296], and polymerase chain reaction [294,295,297]. A CVL experimental infection of 8 female dogs pinpoints that vulvar swab is at least as sensitive as an oral swab for the detection and quantification of *Leishmania* kDNA, and this methodology is proposed to confirm *Leishmania* infection in seropositive dogs [298]. The presence of *L. infantum* amastigotes in the genital tract of naturally infected bitches has been documented [299].

Human African Trypanosomiasis and Animal Trypanosomiases. In humans suffering from sleeping sickness, sterility or infertility, menstrual disorder, a loss of libido, impotence, and amenorrhea have been reported [300]. Testicular damage and clinical manifestation have been described [301], and sexual transmission has been very occasionally observed [302]. *Trypanosoma equiperdum,* responsible for dourine, is a sexually transmitted disease of Equidae [303,304,305]. A loss of fertility is observed in infected animals and is associated with the detection of parasites in semen [306,307]. For *Trypanosoma vivax,* in addition to tsetse flies, transmission routes include transplacental and sexual routes, and parasites were detected in the semen of infected animals [302,308]. In naturally acquired or experimentally induced animal trypanosomiasis caused by *T. brucei* or *T. congolense*, a decrease in semen production associated with an alteration in spermatogenesis has been recorded [309,310,311,312,313]. Histological lesions characterized by testicular degeneration, epididymitis, and epididymal epithelial hyperplasia were detected in the same animals and suggest the participation of the parasite in the etiopathogenic mechanism of reproductive damage, frequently reported in infected animals [308,310,314]. In experimentally infected mice, bioluminescent imaging confirmed the localization of viable trypanosomes in infected mice [315] with an accumulation in the epididymal adipose tissue and in the epididymis [316].

Chagas Disease. The main transmission route of *T. cruzi* is via reduviids, but sexual and transplacental transmission have been described and have epidemiological relevance [317,318]. In 1911, Vianna described testis lesions in experimentally *T. cruzi*-infected guinea pigs [319]. Human orchitis was described in 1916 [320]. The first evidence on the infection of the testis by *T. cruzi* during the acute phase of the disease dates from 1982 [321]. Since then, experimental infection has further shed light on the disseminative capability of this organism during the acute phase of the disease into the male and female genital organs. In a mouse model of infection, *T. cruzi* was detected in the preputial glands and skin, penis, testicular albuginea, epididymis, vas deferens, seminal vesicles, prostate, and urethral glands [322,323]. In females, *T. cruzi* invades cells of the vagina, uterus, oviduct, ovary, and clitoris [313,315]. In addition, *T. cruzi* DNA has been detected in the semen of patients suffering from Chagas disease [317,324]. Limited data exist for humans, but the presence of *T. cruzi* was reported in seminiferous tubules and ovarian cells of children who succumbed to Chagas disease and in menstrual blood of infected patients [30,325].

#### 2.2.6. Milk

The presence of parasites of the Trypanosomatidae family in milk has been probed in view of a maternal transmission risk.

Human and animal leishmaniases. Attempts to test the capacity of *Leishmania* to survive and proliferate in milk were undertaken as early as the 1930s; some evidence on the adequacy of this medium to support *Leishmania* survival was published [326]. Histopathological investigation of female dogs suffering from CVL probed the presence of *Leishmania* amastigotes in the mammary glands [296]. Nevertheless, the presence of *Leishmania* in milk has not yet been reported in patients suffering from leishmaniasis.

Human African Trypanosomiasis and Animal Trypanosomiases. The investigation of trypanosomes in milk has a long history of research and had first focused on the risk of the transmission of pathogens, mainly the risk of transmission of *T. evansi*. Evidence on the presence of *T. evansi* in the milk of lactating cows comes from the work of Zwick and Fisher and was described by Henry and Guilhon in 1944 [327]. During the 1910–1930 period, a set of experimental procedures was employed to detect the presence of various species of *Trypanosoma* species in the milk of experimentally or naturally infected animals [328]. Nathan-Larrier reported that mice and rats experimentally infected by *T. equiperdum* show trypanosomes in their milk [328].

Chagas Disease. Because *T. cruzi*, originally named *Schyzotrypanum cruzi*, possesses the capacity to cross the epithelium and to infect via the oral route [29,327,329], the presence of this pathogen in the milk has been searched for. *T. cruzi* was found in the milk of experimentally infected mice [328,330,331], and several reports describe the presence of this pathogen in the milk from pregnant women [332,333,334,335], as reviewed by Norman and Lopez-Vélez [336]. In most cases, the presence of *T. cruzi* in the milk of pregnant women has been attributed to contamination by infected blood due to nipple bleeding [336]. Therefore, the capacity of *T. cruzi* to invade the mammary gland of infected females has been undertaken. These histological investigations on mice demonstrate the presence of *T. cruzi* amastigotes in the mammary gland alveoli, excretory ducts, the connective tissue envelope of the ducts, inter- and intralobular connective tissue, histiocytes, adipose tissue, the sebaceous glands of the nipple, striated muscle fibers beneath the nipple, and inside the duct lumen [337]. Such proximity of *T. cruzi* parasites with colostrum or milk argues for the inactivation of *T. cruzi* by pasteurization or microwave treatment [338,339].

#### 2.2.7. Nasal Secretion

Human and animal leishmaniases. The presence of *L. donovani* parasites has been detected in the nasal secretions of patients as early as 1936 and reconfirmed sixty years later [119,221]. Parasite DNA can efficiently be detected in this secretion [230]. Parasite DNA has also been detected in the clinically unaffected nasal mucosa of patients infected by *L. braziliensis* [340]. Among the clinical presentation of human leishmaniasis, mucocutaneous alterations are described. They are mainly present in South America and are caused by a restricted number of *Leishmania* species [4]. Nevertheless, this uncommon presentation is also reported to be caused by some Old World species [341], suggesting that nasal secretion deserves further investigation to be confirmed as a positive fluid for *Leishmania* detection.

Human African trypanosomiasis and animal trypanosomiases. No information gathered during the study.

Chagas disease. No information gathered during the study.

#### 2.2.8. Ear Swab/Cerumen

Human and animal leishmaniases. *Leishmania* DNA has been detected and quantified in the cerumen of infected dogs [342]. A recent publication demonstrates that cerumen–qPCR expresses the highest sensitivity (87.5%) to detect genetic materials, followed by hair (lesions: 78.57%, healthy skin: 62.5%), and blood (68.75%) [343]. The ear skin of infected dogs bears a high parasite load compared to other corporal zones and tends to be more infective to sand flies than that of the abdomen [344]. The usefulness of ear swab was investigated in CVL-positive dogs, and positivity of 43% was recorded [230]. In addition, ear lesions caused by *L. mexicana* (Chiclero’s ulcer) are known, but the lesions at this site can be caused by other *Leishmania* species [345].

Human African trypanosomiasis and animal trypanosomiases. No information gathered during the study.

Chagas disease. No information gathered during the study.

#### 2.2.9. Hair/Bristles

Appendages such as hair, bristles, and nails are not referenced as target tissues for trypanosomatid survival and proliferation. Therefore, only a few studies were performed using these materials for the investigation of trypanosomatid infection.

Human and animal leishmaniases. The first series of analyses were performed on dog hair by searching for markers of infection via the analysis of volatile organic compounds. This approach is based on the hypothesis that illnesses can modify odors exhaled by individuals [346] and that canine *Leishmania* infection involves the liberation of some volatile compounds specific to the infection [347,348]. Therefore, with this methodology, it is not the infectious agent that is detected, nor the immunologic response, nor the set of volatile compounds exhaled by the dogs. Although hair is not known as a target tissue for *Leishmania*, an investigation of *Leishmania* DNA was undertaken on CVL in a mouse experimental model of infection with *L. major* but also in the hair of wild mammals or Leporidae [342,349,350]. In mice, the DNA of *L. major* is detected near the inoculation site but also in hair collected in body areas far from the infection site [349]. The first evidence on the usefulness of PCR performed on hair to act as a biomarker of infectiousness of the host came from CVL [351]. The rationale for such an accumulation of parasite DNA into the hair of the infected animal is not entirely understood. The hypothesis of a transdermal elimination process has been raised. This process, observed as a secondary component of primary skin diseases, includes the elimination of endogenous substances but also exogenous infectious organisms, such as *Mycobacterium tuberculosis* or HIV [352]. It requires the direct incorporation of the parasite DNA among skin and hair keratinocytes at the site of inoculation. The intracellular infection of keratinocytes with *Leishmania* was not detected following the infection of C57Black/6j mice [353], but the presence of *Leishmania* amastigotes has been observed in the hair follicles of patients with cutaneous leishmaniasis [352].

Human African trypanosomiasis and animal trypanosomiases. No information gathered during the study.

Chagas disease. No information gathered during the study.

### 2.3. Meta-Analysis of Non-Invasive Sampling Methodologies for Trypanosomatid Infection Diagnosis: Overview and Limitations

Meta-analyses on value for the diagnosis of leishmaniasis, of rapid diagnosis tests (RDTs) of VL, and on urine as a biological sample have recently been published [170,172]. The results from these analyses disclose the interest of 2 tests, an antibody detection test (rK39), and an antigen detection test (KAtex) for VL confirmatory diagnosis. Here, we performed a meta-analysis on the positivity of various non-invasive methodologies for diagnosing human and animal infections with parasites belonging to the Trypanosomatidae family, to delineate their potential as an alternative to invasive biopsies for disease diagnosis and pathogen tracking. It is, therefore, a comparative study. Data were extracted from selected papers according to the following criteria: (1) diagnosis performed on the basis of clinical symptoms and confirmed by PCR, ELISA, IFI, or parasitology; and (2) data available for individual subjects or grounded according to clinical symptoms and diagnosis confirmation (see Section 4 for the methodology).

Overview and limitation of the collected data for meta-analysis. A large majority of studies focus on urine as a biological sample, primarily in *Leishmania* infection and CD. Strikingly, in urine, *Leishmania* parasites, antibodies, antigens, and genetic material have been tracked. A test based on the detection of *Leishmania* antigen in urine is commercialized. Although the detection of antigens in urine has been exclusively performed on human VL, a large majority of studies have focused on the detection of DNA in human patients. For Chagas disease, the main area of investigation has been the detection of *T. cruzi* antigen in urine, and the search for *T. cruzi* DNA is reported in only one publication, but this discusses the detection of DNA in an experimental model of infection [179]. Surprisingly, we did not find any information on the detection of HAT in the urine of human patients or on AAT. Saliva is the second body secretion used to track *Leishmania* infection. In saliva, the search for the presence of antibodies directed against *T. cruzi* is reported for CD. Saliva is also the sole body secretion investigated for HAT diagnosis [235,236]. The third most investigated secretion is the lacrimal fluid that is collected at the same time as conjunctival cells by the use of conjunctival swabs. All these studies have been performed on dogs or cats or other mammals using PCR, qPCR, or PCR hybridization (kDNA). Finally, partial information on the presence of antibodies, antigens, DNA, or parasites is currently published for other materials (e.g., feces, hair/bristle, milk, cerumen, nasal secretion). For all these reasons, an exhaustive meta-analysis of all the alternative non-invasive biological samples for diagnosis of diseases caused by Trypanosomatidae cannot be performed. Genetic material detection has been tested in urine, saliva, nasal, cerumen, and conjunctival swab. Nevertheless, for conjunctival swabs, data are available only for canine visceral leishmaniasis. Other limitations are the wild diversity of methodological approaches used to diagnose the disease or to detect pathogens that have been employed. Immunological approaches included antigen and antibody detection. Within these, the agglutination test, ELISA, immunochromatography, or Western blot were used. For molecular methods, PCR, qPCR, and LAMP were employed. For parasitological methods, in vitro culture or biopsy staining were used. Lastly, some studies included HIV positive and negative patients. In this meta-analysis, we cannot perform subgroup analysis by technical approaches or HIV status because of the lack of data to perform it.

Overall data were extracted from 77 papers (see Table 2). A majority of papers (88%) focused on *Leishmania* infection, 9.6% on Chagas disease, and 2.4% on HAT. Strikingly, we did not find studies on AAT diagnosis with non-invasive biological sampling that can be included in this meta-analysis (Table S3).

Within papers dealing with leishmanioses, more than 50% focused on VL due to *L. infantum* (syn *L. chagasi*) to diagnose CVL. For CD, the majority of the studies discussed the use of urine to diagnose the disease with immunological approaches, mainly antigen capture. Only two papers investigated alternative biological sampling for sleeping sickness diagnosis and tested antibody-based detection in the saliva.

Random effect meta-analyses were carried out using the total sample size and the number of positive samples (effect size, standard error of effect size) to estimate the positivity of the non-invasive sampling methods of each methodological approach. We therefore analyzed pooled estimates by random effects analysis. The results of the heterogeneity test (Table 3) depict high heterogeneity in terms of the I^2^ statistic (100%).

#### 2.3.1. Urine

Urine is by far the most studied body secretion investigated for the presence of antibody, antigen, parasite, or genetic material, mainly in Leishmaniasis and Chagas disease, for antigen detection.

**Figure 3:**
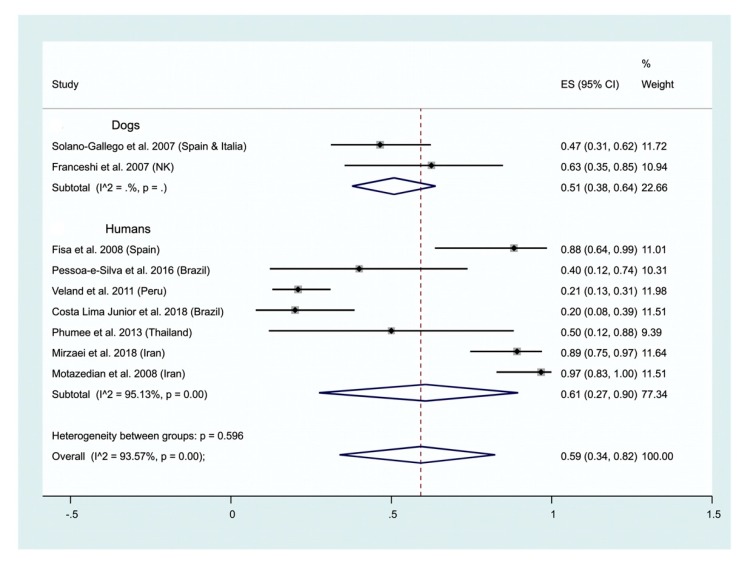
Forest plot representation of the extracted data for urine analysis using molecular methods on leishmaniasis with subgroup analysis on dogs and humans.

Human and animal leishmaniases. A positivity of 59% (95% CI; 34–82%) is recorded when human or dog urine are used to detected the genetic material in urine of human or dogs (Figure 3). The test of heterogeneity between studies depicts high a heterogeneity (I^2^ = 93.57%). Subgroup analysis depict a positivity rate of 61% (95% CI; 27–90%; I^2^ = 93.57%) for human urine, that is higher than those recorded for dogs, 51% (95% CI; 38–64%) (Figure 3).

**Figure 4:**
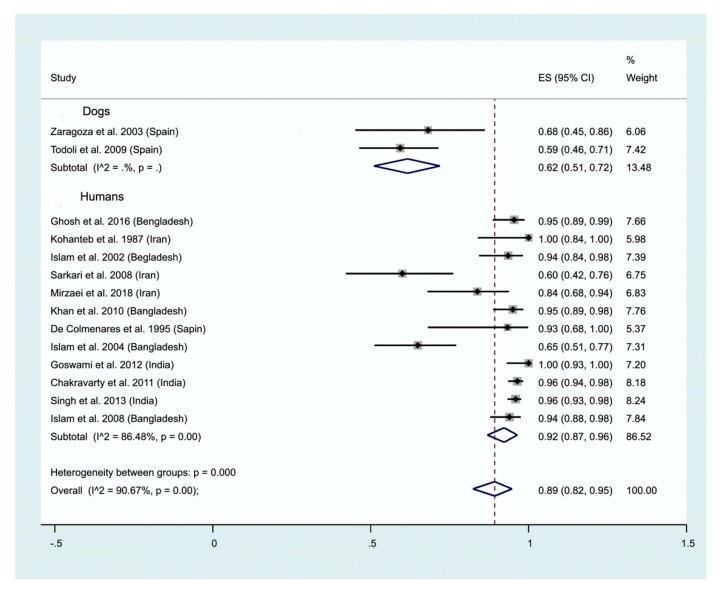
Forest plot representation of the extracted data for urine analysis using antibody detection methods on leishmaniasis with subgroup analysis on dogs and humans.

**Figure 6:**
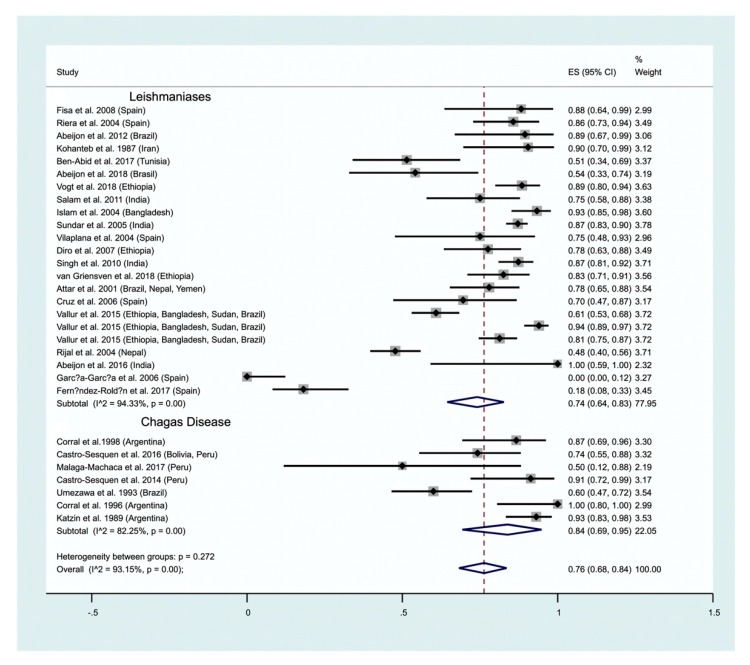
Forest plot representation of the extracted data for urine analysis using antigen detection methods with subgroup analysis on human leishmaniasis and Chagas disease.

Antibody detection has also been carried out using urine, mainly in humans but rarely in dogs. A high positivity of 92% (95% CI: 87–96) is recorded in humans (Figure 4, lower panel), which is lower in dogs (62%; 95% CI: 51–72; Figure 4, upper panel). Only two studies on dogs satisfy our inclusion criteria and were analyzed as contrasted to the 12 studies carried out on humans (Figure 4). The test of heterogeneity between studies on Ab detection in human urine depicts a rather high heterogeneity (I^2^ = 86.48%). A positivity of 74% (95% CI: 64–83) is recorded for antibody detection in humans. The parasitological method represents a simple way to detect leishmania infection, which has been applied to dogs suffering from CVL (1 study), and humans (4 studies). An overall positivity of 28% is recorded (95% CI: 0–76; Figure 5). Antigen detection in urine is used for the diagnosis of visceral leishmaniasis (see Section 2.2.1). Our meta-analysis highlights a 74% (95% CI: 64–83) positivity for the detection of antibodies in urine. We have not performed subgroup analysis according to the methodology used (ELISA, other) or the infecting leishmania species that is reflected by the high heterogeneity (I^2^ = 94.33%) (Figure 6).

Human African Trypanosomiasis and Animal Trypanosomiases. Analysis cannot be performed due to a lack of quantitative data.

Chagas Disease. Ag detection has been carried out several times on patients’ urine and 7 studies satisfying our inclusion criteria were gathered (Figure 6). We recorded a higher positivity for antigen detection in CD (84%; 95% CI: 69–95) than for Leishmaniasis (74%), see above. Our analysis further confirms that Ag detection in a patient’s urine represents an interesting fluid for CD and Leishmanioses diagnosis.

#### 2.3.2. Feces

No quantitative data available.

#### 2.3.3. Saliva/Oral Swab/Sputum

**Figure 7:**
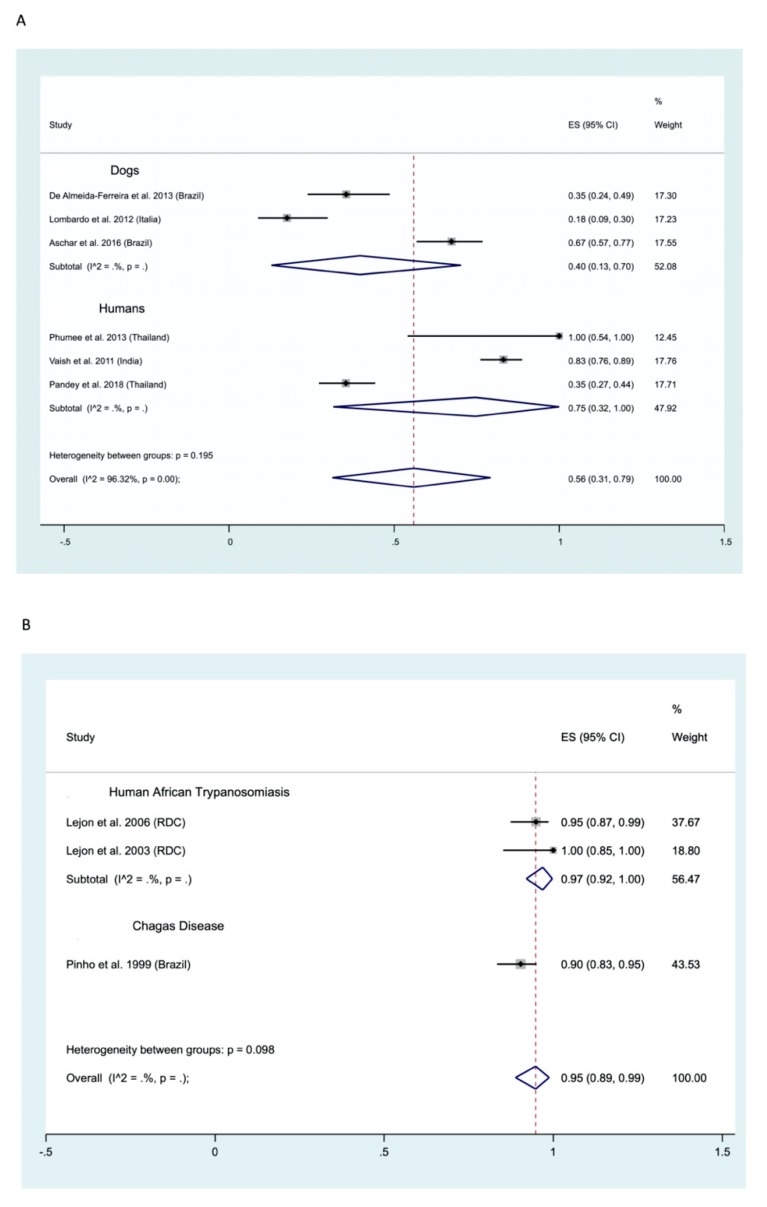
Forest plot representation of the extracted data for saliva analysis using molecular detection methods for human leishmaniases (**A**) and antibody detection methods with subgroup analysis for human African trypanosomiasis and Chagas disease (**B**).

Human and animal leishmaniases. Molecular methods were used to diagnose leishmania infection using the saliva of humans and dogs. Six studies were collected and data were extracted (3 for dogs and 3 for humans) (Figure 7). From these data, we recorded an overall positivity of 56% (CI 95%: 31–79), positivity being higher in humans, 75% (CI 95%: 32–100), than in dogs where it is of 40% (CI 95%: 13–70). Additional data is required to more firmly address the interest of using molecular methods to detect leishmania parasites using saliva. Data on the detection of antigen in patients suffering leishmaniasis has been extracted from only one study with a positivity of 74% [129].

Human African Trypanosomiasis and Animal Trypanosomiases. The two studies on HAT disclosed a positivity of 97% (95% CI: 92–100) for antibody detection in the saliva.

Chagas Disease. Only one study that respected our inclusion criteria was collected. In this study, a high positivity was recorded, 90%, that points to the need for additional experiments.

#### 2.3.4. Conjunctival Swab/Lacrimal Fluid

**Figure 8:**
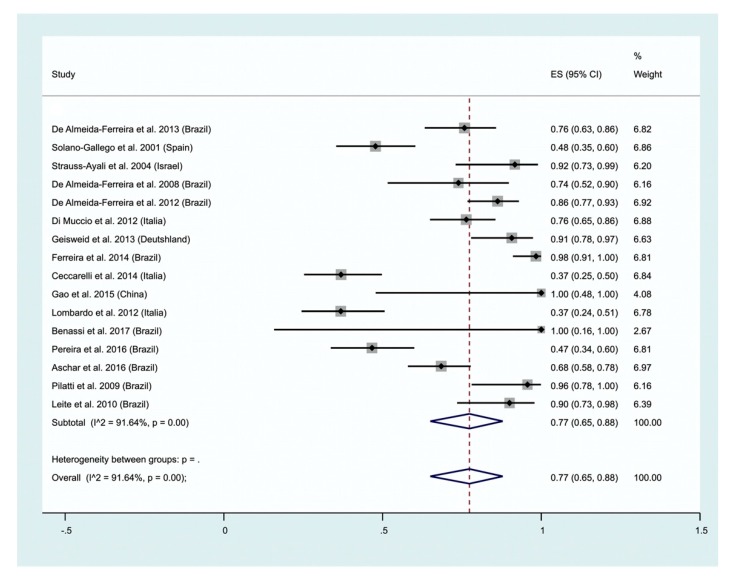
Forest plot representation of the extracted data for conjunctival swab/lacrimal fluid using molecular detection methods on canine visceral leishmaniasis.

Human and animal leishmaniases. Only data on CVL using molecular methods were gathered during the survey (Figure 8). An overall positivity of 77% was recorded (95% CI; 65–88) and a high heterogeneity was recorded (I^2^ 91.64%). Data on the use of parasitological (histology) methods was extracted from one study performed on CVL, with a positivity of 53% (CI 95%: 40–66) [25].

Human African Trypanosomiasis and Animal Trypanosomiases. Analysis cannot be performed due to a lack of quantitative data.

Chagas Disease. Analysis cannot be performed due to a lack of quantitative data.

#### 2.3.5. Genital Organs: Semen/Vulvular Secretions

No quantitative data available to perform a meta-analysis on the positivity.

#### 2.3.6. Milk

No quantitative data available to perform a meta-analysis on the positivity.

#### 2.3.7. Nasal Secretion

**Figure 9:**
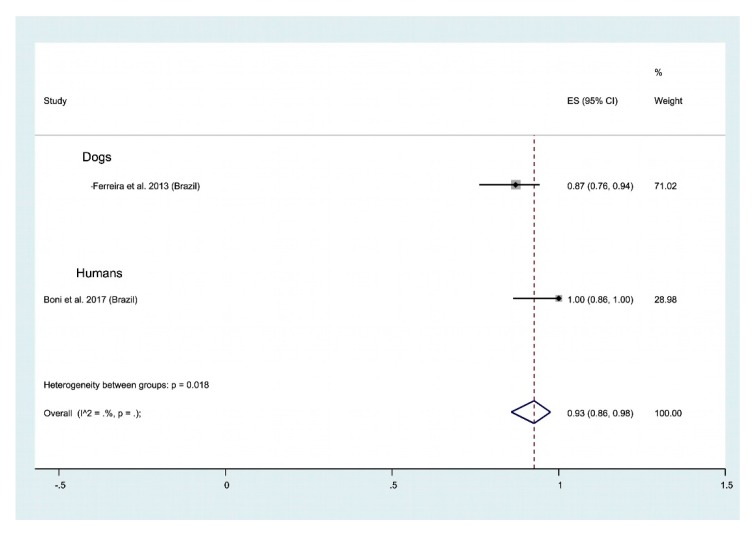
Forest plot representation of the extracted data for nasal secretion using molecular detection methods on dogs and human suffering leishmaniasis.

Human and animal leishmaniases. Molecular methods applied for the detection of Leishmania genetic material was used on dogs (1 publication) and on humans (1 publication). From these publications, we recorded a very high overall positivity of 93% (CI 95%; 86–96) (Figure 9). Additional data are required to further explore the suitability of this fluid for DNA detection and leishmaniasis diagnosis. In addition, one study reported the isolation of leishmania parasites in nasal secretion using parasitological methods, with a positivity of 36% (95% CI: 24–49) [119].

Human African Trypanosomiasis and Animal Trypanosomiases. Analysis cannot be performed due to a lack of quantitative data.

Chagas Disease. Analysis cannot be performed due to a lack of quantitative data.

#### 2.3.8. Hair/bristles

**Figure 10:**
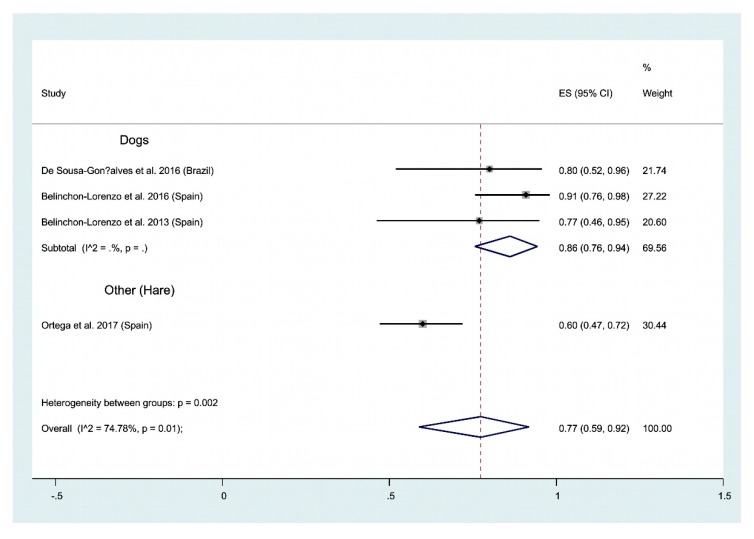
Forest plot representation of the extracted data for hair/bristles using molecular detection methods.

Human and animal leishmaniases. During the past 10 years, molecular methods has been applied to investigate the suitability of hair or bristle to detect Leishmania genetic parasite material. These experiments were performed on the domestic reservoir of Leishmania (dogs), but also on potential wild reservoir (hare). In all cases, positivity greater than 60% were recorded. Altogether, our meta-analysis points to an an overall positivity of 77% (CI 95%: 59–92) (Figure 10). To further investigate the potential of hair/bristles to be used to detect leishmania infection, additional experiments are required.

Human African Trypanosomiasis and Animal Trypanosomiases. Analysis cannot be performed due to a lack of quantitative data.

Chagas Disease. Analysis cannot be performed due to a lack of quantitative data.

## 3. Materials and Methods

### 3.1. Protocol and Registration

The current study was conducted taking into account the recommendation of the PRISMA statement [377]. The protocol was neither registered nor published. The checklist for meta-analysis is provided as Supplementary Material (Check list S1).

### 3.2. Information Source

The selection of studies was based on searches (performed in November 2018) in PubMed, Web of Science, WorldWideScience, SciELO, Embase, and Google, with no specific year range and language limitation.

### 3.3. Search

The search was performed using the subject headings “Leishmania”, “Leishmaniasis”, “Trypanosoma”, “Trypanosomiasis”, and “Chagas disease” combined with several keywords, including “urine”, “lacrimal”, “conjunctival”, “ocular”, “eyes”, “saliva”, “sputum”, “oral swab”, “ear swab”, “buccal swab”, “cerumen”, “semen”, “vulvular”, “feces”, “fecal”, “hair”, “skin”, “nasal swab”, “nostril”, “pharyngeal”, “mucosal”, “milk”, and “sweat”.

An example of the search strategy in PubMed is below:

1. “Leishmania”,
2. “Leishmaniasis”,
3. “Trypanosoma”,
4. “Trypanosomiasis”, and
5. “Chagas disease”,

combined with several key words

6. “urine”,
7. “lacrimal”,
8. “conjunctival”,
9. “ocular”,
10. “eyes”,
11. “saliva”,
12. “sputum”,
13. “oral swab”,
14. “ear swab”,
15. “buccal swab”,
16. “cerumen”,
17. “semen”,
18. “vulvular”,
19. “feces”, “fecal”,
20. “hair”,
21. “skin”,
22. “nasal swab”,
23. “nostril”,
24. “pharyngeal”,
25. “mucosal”,
26. “milk”, and
27. “sweat”.

### 3.4. Study Selection

We undertook the review with current recommendations reported in 2015 and reported our findings as per the PRISMA guidelines, taking into account the remarks for “biological” meta-analyses, which deal with non-human species [378]. We selected studies for inclusion in two stages. In the first stage, we screened the titles and abstracts of all citations for potentially relevant papers. In the second, we examined the full texts of these papers. Two independent reviewers (Denis SERENO and Mohammad AKHOUNDI) performed the screening and the full text and data extraction. For each record, to ensure an objective assessment of all the included records, the judgements about eligibility, bias, and applicability were entirely based on the published documents and not on unpublished background information. In case of discrepent judgement, Denis SERENO took the final decision. Studies that involved the following topics were eligible for selection:

1- Leishmaniases, Chagas disease, or trypanosomiases
2- Identification and/or diagnosis with molecular, immunological, or parasitological methods
3- Experimentally or naturally acquired infection.

Duplicates and studies that did not include trypanosomatid detection, disease diagnosis, or the detection of antibodies, antigens, DNA, RNA, or other molecules were ruled out of the systematic review.

For meta-analysis, data were extracted according to the following criteria: (1) the use of non-invasive biological sampling methods, (2) diagnosis made on the basis of clinical symptoms and confirmed by various methodologies (PCR, polymerase chain reaction; ELISA, enzyme linked immnosorbent assay; IFI, indirect mmmunofluorescence; or parasitology), (3) confirmation performed using biopsy (blood, lymph node, or spleen aspirate); and (4) data available for individual subjects or grounded according to clinical symptoms and diagnosis confirmation.

### 3.5. Data Collection Process and Items

We developed a data collection sheet to gather data items from studies. The data collection sheets included the following: first author name, the title and article year of publication, country, methodologies used to diagnose infection, the name of the alternative biological sampling performed, the total number of samples processed, the total number of positive samples, the total number of positive samples for alternative biopsies, and the methods of detection used in the non-invasive biological sampling. The databases containing the data collection sheet are shown as Supplementary Material (Datacollection S2).

### 3.6. Statistical Analysis

Effect size in this study was the prevalence and percentage; the variance of the effect size was not stable because some percentages were near or equal to 100 and some were near zero; therefore, a Freeman–Tukey double arcsine transformation was used to stabilize the variances [379,380]. We checked the heterogeneity between studies by the I^2^ statistic. The random effects model was used to combine effect sizes among studies. A meta-regression was performed to investigate the diversity of pooled effect size according to year of publication. Begg and Egger’s tests were used to assess publication bias in the included studies [381]. Subgroup analysis was performed based on biological sample origin and methodology. Data were analyzed using Stata computer software version 11.2 (StataCorp, College Station, TX, USA).

## 4. Conclusions

Literature analysis reveals striking facts and gaps in the usefulness of body fluid secretions and/or appendages for the diagnosis of infection caused by Trypanosomatidae parasites. Strikingly, we did not find data on the presence of parasites, DNA, antibodies, or antigens in the sweat of infected patients. We confirm the interest of antibody and antigen detection methods in urine to diagnose leishmaniases, and of Antigen detection in the urine of patients suffering CD. Surprisingly a high positivity was recorded for *Leishmania* detection when hair was used in combination with molecular methods. This observation, which relies on a limited number of data, has now to be thoroughly investigated. With some exceptions, we recorded a serious lack of experimental and clinical investigations on the appropriateness of alternative biological sampling methodologies for diagnostic purposes. These data are important to set up new non-invasive diagnosis protocols to track disease evolution and clinical and/or chemotherapy success. This is surprising because these alternative sampling strategies present a major advantage of being based on the collection of daily produced biological materials.

## Figures and Tables

**Figure 5:**
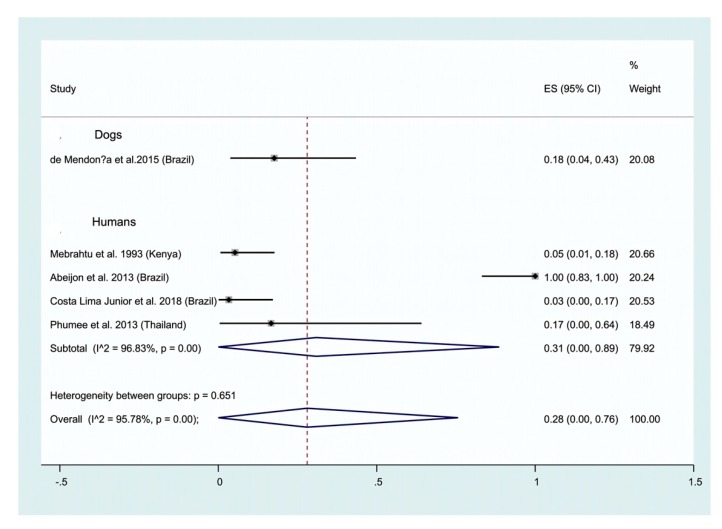
Forest plot representation of the extracted data for urine analysis using parasitological methods on leishmaniasis with subgroup analysis on dogs and humans.

**Table 1:** Methodologies to diagnose Chagas disease (CD), animal trypanosomiases (AT), human African trypanosomiasis (HAT), and leishmaniosis, and/or to detect their respective causative agents.

Methodologies			Quantification	Culture	CD Detection and Identification	AT Detection and Identification	HAT Detection and Identification	*Leishmania* Detection and Identification	Ref
DNA/RNA-based Methods	PCR	PCR/qPCR/Multiplex	yes	no	yes/DTU	yes/sp	yes/sp	yes/sp	[77,78,79]
		PCR–OC	no	no	NA/NA	yes/sp	yes/sp	yes/sp	[80,81]
		PCR–ELISA	yes	no	NA/NA	yes/sp	yes/sp	yes/sp	[82,83,84,85]
		PCR–HRM	no	no	yes/DTU	NA/NA	NA/NA	yes/sp	[63,86]
		PCR–RFLP	no	no	yes/DTU	yes/sp	yes/sp	yes/sp	[87,88,89]
		PCR-sequencing	no	no	yes/DTU	yes/sp	yes/sp	yes/sp	[78,88]
	Other	PFGE	no	yes	yes/NA	yes/sp	yes/sp	no/sp	[88,90,91]
		NASBA	no	no	NA/NA	NA/NA	yes/sp	yes/sp	[81]
		LAMP	poss	no	yes/no	yes/sp	yes/sp	yes/sp	[69,92]
Non DNA-based Methods	Parasitology	Microscopic examination	yes	no	yes/no	yes/no	yes/no	yes/no	[2,19,27,93]
		In vitro parasite culture	no	yes	yes/no	yes/no	yes/no	yes/no	/
		Isolation in experimental animals	no	no	yes/no	NA/NA	yes/no	yes/no	/
		Xenodiagnosis	no	no	yes/no	NA/NA	yes/no	yes/no	[94,95,96,97]
		Dermal diagnostic tests	no	no	NA/NA	NA/NA	NA/NA	yes/no	[98]
	Immunology/Serology	ELISA Ab	no	no	yes/no	yes/no	yes/no	yes/no	[99,100,101]
		ELISA Ag	no	no	yes/no	yes/no	NA/NA	yes/no	[102]
		IFAT	no	no	yes/no	yes/no	yes/no	yes/gen	[71,72,73,103,104]
		ICT Ag	no	no	NA/NA	NA/NA	NA/NA	yes/gen	[105]
		ICT Ab	no	no	NA/NA	NA/NA	yes/no	yes/no	[106,107]
		DAT/CATT	no	no	yes/yes	yes/yes	yes/yes	yes/yes	[74,99,100,108]
		Western blot	no	no	yes/no	NA/NA	NA/NA	yes/sp	[109,110]
	Protein-based methods	MLEE	no	yes	no/DTU	no/sp	no/sp	no/sp	/
		MALDI-TOF	no	yes	no/DTU	NA/NA	no/sp	no/sp	[111,112]

gen: genera. sp: species. DTU: discrete typing unit. NA: not available. DAT: direct agglutination test. CATT: card agglutination test for trypanosomiasis. MLEE: multilocus enzymatic electrophoresis. MALDI–TOF: matrix-assisted laser desorption ionization–time of flight. ICT: immunochromatographic test. ELISA: enzyme-linked immunosorbent assay. PCR–OC: polymerase chain reaction with oligochromatography. LAMP: loop-mediated isothermal amplification. Ab: antibody. Ag: antigen. PFGE: pulse field gel electrophoresis. NASBA: nucleic acid sequence-based amplification. PCR: polymerase chain reaction. HRM: high melting resolution. RFLP: restriction fragment length polymorphism.

**Table 2:** References and characteristics of the studies from which data can be extracted.

Ref	Author/year	Country	Pathogen	Host	Clinic	HIV	Methodology	Sample	Sa Size
[119]	Mebrahtu 1993	Kenya	*L. donovani*	Human	VL	No	Parasitology	U	64
[120]	da Costa Lima 2018	Brazil	*L. infantum*	Human	VL	No	Parasitology and genetic material	U	30
[133]	Pessoa-E-Silva 2016	Brazil	*L. infantum*	Human	VL	Yes	Parasitology	U	18
[354]	de Mendonça 2015	Brazil	*L. infantum*	Dog	CVL	NA	Parasitology	U	17
[130]	Veland 2011	Peru	*Leishmania* spp.	Human	CL-MCL	No	Genetic material	U	86
[131]	Solano-Gallego 2007	Spain and Italy	*L. infantum*	Dog	CVL	NA	Genetic material	U	43
[109]	Mirzaei 2018	Iran	*Leishmania* spp.	Human	VL-CL	No	Genetic material-Immunology/Ab	U	37
[168]	Motazedian 2008	Iran	*L. infantum*	Human	VL	No	Genetic material	U	30
[121]	Fisa 2008	Spain	*L. infantum*	Human	VL	Yes	Genetic material	U	17
[135]	Franceschi 2007	NK	*Leishmania* spp.	Dog	CVL	NA	Genetic material	U	16
[167]	Sundar 2005	India	*L. donovani*	Human	VL	No	Immunology/Ag	U	382
[144]	Singh 2013	India	*L. donovani*	Human	VL	No	Immunology/Ab-Ag	U	365
[145]	Chakravarty 2011	India	*L. donovani*	Human	VL	No	Immunology/Ab	U	280
[163]	Vallur 2015	Ethiopia, Bangladesh, Sudan, Brazil	*Leishmania* spp.	Human	VL	No	Immunology/Ag	U	166
[165]	Rijal 2004	Nepal	*L. donovani*	Human	VL	No	Immunology/Ab	U	155
[105]	Singh 2010	India	*L. donovani*	Human	VL	No	Immunology/Ag-Ab	U	150
[141]	Islam 2008	Bangladesh	*L. donovani*	Human	VL	No	Immunology/Ab	U	115
[143]	Khan 2010	Bangladesh	*L. donovani*	Human	VL	No	Immunology/Ab	U	100
[355]	Ghosh 2016	Bangladesh	*L. donovani*	Human	VL	No	Immunology/Ab	U	87
[148]	Diro 2007	Ethiopia	*L. donovani*	Human	VL	No	Immunology/Ag-Ab	U	87
[356]	Vogt 2018	Ethiopia	*L. donovani*	Human	VL	No	Immunology/Ag	U	87
[140]	Islam 2004	Bangladesh	*L. donovani*	Human	VL	No	Immunology/Ab	U	75
[156]	Todolí 2009	Spain	*L. infantum*	Dog	CVL	NA	Immunology/Ab	U	64
[171]	van Griensven 2018	Ethiopia	*L. donovani*	Human	VL	No	Immunology/Ag	U	63
[139]	Islam 2002	Bangladesh	*L. donovani*	Human	VL	No	Immunology/Ab	U	62
[194]	Umezawa 1993	Brazil	*T. cruzi*	Human	CD	No	Immunology/Ag	U	60
[76]	Attar 2001	Brazil, Nepal, Yemen	*Leishmania* spp.	Human	VL	No	Immunology/Ag	U	59
[193]	Katzin 1989	Argentina	*T. cruzi*	Human	CD	No	Immunology/Ag	U	58
[169]	Riera 2004	Spain	*L. infantum*	Human	VL	Yes	Immunology/Ag	U	49
[357]	Fernández-Roldán 2017	Spain	*L. infantum*	Human	VL	No	Immunology/Ag	U	44
[146]	Goswami 2012	India	*L. donovani*	Human	VL-PKDL	yes	Immunology/Ab	U	42
[358]	Salam 2011	India	*L. donovani*	Human	VL	No	Immunology/Ag	U	36
[99]	Sarkari 2008	Iran?	*Leishmania* spp.	Human	VL	No	Immunology/Ag-Ab	U	35
[129]	Ben-Abid 2017	Tunisia	*L. infantum*	Human	VL	No	Immunology/Ag	U-S	35
[201]	Castro-Sesquen 2016	Bolivia, Peru	*T. cruzi*	Human	CD	No	Immunology/Ag	U	31
[359]	Corral 1998	Argentina, Paraguay	*T. cruzi*	Human	CD	No	Immunology/Ab	U	30
[360]	García-García 2006	Spain	*L. infantum*	Human	VL	Yes	Immunology/Ab	U	28
[361]	Abeijon 2018	Brazil	*L. infantum*	Human	VL	No	Immunology/Ag	U	24
[362]	Cruz 2006	Spain	*L. infantum*	Human	VL	No	Immunology/Ag	U	23
[200]	Castro-Sesquen 2014	Bolivia, Peru	*T. cruzi*	Human	CD	No	Immunology/Ag	U	23
[153]	Zaragoza 2003	Spain	*L. infantum*	Dog	CVL	NA	Immunology/Ag	U	22
[363]	Abeijon 2013	Brazil	*L. infantum*	Human	VL	No	Immunology/Ag	U	20
[138]	Kohanteb 1987	Iran	*L. infantum?*	Human	VL	No	Immunology/Ag-Ab	U	21
[159]	Abeijon 2012	Brazil	*L. infantum*	Human	VL	No	Immunology/Ag	U	19
[192]	Corral 1996	Argentina	*T. cruzi*	Human	CD	No	Immunology/Ag	U	17
[166]	Vilaplana 2004	Spain	*L. infantum*	Human	VL	yes	Immunology/Ag	U	16
[152]	De Colmenares 1995	Spain	*L. infantum*	Human	VL	No	Immunology/Ag	U	15
[364]	Abeijon 2016	India	*L. donovani*	Human	VL	No	Immunology/Ag	U	7
[197]	Málaga-Machaca 2017	Peru	*T. cruzi*	Human	CD	No	Immunology/Ag	U	6
[227]	Vaish 2011	India	*L. donovani*	Human	VL	No	Genetic material	S	148
[225]	Pandey 2018	Thailand	*L. martiniquensis?*	Human	VL	Yes	Genetic material	S	130
[365]	Boni 2017	Brazil	*Leishmania* spp.	Human	CL-MCL	No	Genetic material	S-N	15
[134]	Phumee 2013	Thailand	*L. martiniquensis*	Human	VL-CL	No	Genetic material and Parasitology	S-U	6
[238]	Pinho 1999	Brazil	*T. cruzi*	Human	CD	No	Immunology/Ab	S	114
[235]	Lejon 2003	DRC	*T. b. brucei*	Human	SleepS	No	Immunology/Ab	S	78
[236]	Lejon 2006	DRC, Benin	*T. b. brucei*	Human	SleepS	No	Immunology/Ab	S	23
[25]	Peña 2008	Spain	*L. infantum*	Dog	CVL	NA	Parasitology	O	60
[366]	Aschar 2016	Brazil	*L. infantum*	Dog	CVL	NA	Genetic material	O-S	92
[367]	de Almeida Ferreira 2012	Brazil	*L. infantum*	Dog	CVL	NA	Genetic material	O	80
[368]	Di Muccio 2012	Italy	*L. infantum*	Dog	CVL	NA	Genetic material	O	72
[369]	Solano-Gallego 2001	Spain	*L. infantum*	Dog	CVL	NA	Genetic material	O	67
[370]	Ceccarelli 2014	Italy	*L. infantum*	Dog	CVL	NA	Genetic material	O	65
[230]	Ferreira 2013	Brazil	*L. infantum*	Dog	CVL	NA	Genetic material	O-S-N	62
[266]	Pereira 2016	Brazil	*L. infantum*	Dog	CVL	NA	Genetic material	O	60
[371]	Carvalho Ferreira 2014	Brazil	*L. infantum*	Dog	CVL	NA	Genetic material	O	60
[229]	Lombardo 2012	Italy	*L. infantum*	Dog	CVL	NA	Genetic material	O-S	57
[265]	Ferreira 2008	Brazil	*L. infantum*	Dog	CVL	NA	Genetic material	O	46
[372]	Geisweid 2013	Germany	*L. infantum*	Dog	CVL	NA	Genetic material	O	43
[268]	Leite 2010	Brazil	*L. infantum*	Dog	CVL	NA	Genetic material	O	30
[264]	Strauss-Ayali 2004	Israel	*L. infantum*	Dog	CVL	NA	Genetic material	O	24
[373]	Pilatti 2009	Brazil	*L. infantum*	Dog	CVL	NA	Genetic material	O	23
[374]	Gao 2015	China	*L. infantum*	Dog	CVL	NA	Genetic material	O	5
[269]	Benassi 2017	Brazil	*Leishmania*	Cat	?	NA	Genetic material	O	2
[350]	Ortega 2017	Spain	*Leishmania*	*Leporidae*	?	NA	Genetic material	H	65
[375]	Belinchón-Lorenzo 2016	Brazil	*L. infantum*	Dog	CVL	NA	Genetic material	H	30
[376]	Belinchón-Lorenzo 2013	Spain	*L. infantum*	Dog	CVL	NA	Genetic material	H	28
[351]	de Sousa Gonçalves 2016	Brazil	*L. infantum*	Dog	CVL	NA	Genetic material	H	15

Genetic material: refers to all molecular methodologies that amplify/quantify DNA or RNA independent of the molecular method of amplification. Immunology: refers to all methodologies that use antibodies to detect/quantify antigen and vice versa. Parasitology: refers to methodologies involving parasite observation after in vitro culture, sedimentation, or staining of a smear. CVL: canine visceral leishmaniasis. VL: visceral leishmaniasis. CL: cutaneous leishmaniasis. MCL: mucocutaneous leishmaniasis. CD: Chagas disease. SleepS: sleeping sickness. NK: not known. NA: not applicable. U: urine. O: ocular. S: saliva. N: nasal. H: hair. C: cerumen/ear0. The only two studies gathered on *T. brucei*, in bold, were excluded from the subgroup analysis.

**Table 3:** Subgroup analysis and test for effect size heterogeneity.

	Positivity (95% CI)	Heterogeneity			
		df	*p*	I^2^ (%)	Tau^2^
Positive PCR in Urine	0.73(0.63–0.84)	8	<0.0001	100	0.250
Positive Ab in Urine	0.57(0.37–0.76)	13	<0.0001	100	0.139
Positive Ag in Urine	0.59(0.47–0.72)	28	<0.0001	100	0.117
Positive Parasite in Urine	0.85(0.80–0.91)	5	<0.0001	100	0.004
Positive PCR in Conjunctival Swab	0.56(0.30–0.81)	17	<0.0001	100	0.306
Positive PCR in Oral Saliva	0.57(0.28–0.85)	6	<0.0001	100	0.148
Positive PCR in Nasal	0.73(0.48–0.98)	1	<0.0001	100	0.033

## Supplementary Materials

The following are available online at https://www.mdpi.com/1422-0067/21/5/1684/s1, Check list of the Meta analysis and systematic review: ChecklistS1; Data collection sheet for meta analysis: DatacollectionS2, Table of the collected data for meta analysis: TablerecapS3.

## Abbreviations

HAT	Human African Trypanosomiasis
CD	Chagas disease
AT	Animal Trypanosomiasis
AAT	African Animal Trypanosomiasis
CVL	Canine visceral leihmaniasis

## References

[1] Simpson A.G.B., Stevens J.R., Lukeš J. (2006). The evolution and diversity of kinetoplastid flagellates. Trends Parasitol..

[2] Brun R., Blum J., Chappuis F., Burri C. (2010). Human African trypanosomiasis. Lancet.

[3] Wang Y., Utzinger J., Saric J., Li J.V., Burckhardt J., Dirnhofer S., Nicholson J.K., Singer B.H., Brun R., Holmes E. (2008). Global metabolic responses of mice to Trypanosoma brucei brucei infection. Proc. Natl. Acad. Sci. USA.

[4] Akhoundi M., Kuhls K., Cannet A., Votýpka J., Marty P., Delaunay P., Sereno D. (2016). A Historical Overview of the Classification, Evolution, and Dispersion of Leishmania Parasites and Sandflies. PLoS Negl. Trop. Dis..

[5] Sereno D. (2019). Leishmania (Mundinia) spp.: From description to emergence as new human and animal Leishmania pathogens. New Microbes New Infect..

[6] Truc P., Büscher P., Cuny G., Gonzatti M.I., Jannin J., Joshi P., Juyal P., Lun Z.-R., Mattioli R., Pays E. (2013). Atypical human infections by animal trypanosomes. PLoS Negl. Trop. Dis..

[7] Ebhodaghe F., Ohiolei J.A., Isaac C. (2018). A systematic review and meta-analysis of small ruminant and porcine trypanosomiasis prevalence in sub-Saharan Africa (1986 to 2018). Acta Trop..

[8] Ebhodaghe F., Isaac C., Ohiolei J.A. (2018). A meta-analysis of the prevalence of bovine trypanosomiasis in some African countries from 2000 to 2018. Prev. Vet. Med..

[9] Stuart K., Brun R., Croft S., Fairlamb A., Gürtler R.E., McKerrow J., Reed S., Tarleton R. (2008). Kinetoplastids: Related protozoan pathogens, different diseases. J. Clin. Investig..

[10] Silva F.L., Oliveira R.G., Silva T.M.A., Xavier M.N., Nascimento E.F., Santos R.L. (2009). Venereal transmission of canine visceral leishmaniasis. Vet. Parasitol..

[11] de Oliveira V.V.G., Alves L.C., da Silva Junior V.A. (2015). Transmission routes of visceral leishmaniasis in mammals. Ciênc. Rural.

[12] Turchetti A.P., Souza T.D., Paixão T.A., Santos R.L. (2014). Sexual and vertical transmission of visceral leishmaniasis. J. Infect. Dev. Ctries..

[13] SYMMERS W.S. (1960). Leishmaniasis acquired by contagion: A case of marital infection in Britain. Lancet.

[14] Bosch R.J., Rodrigo A.B., Sánchez P., de Gálvez M.V., Herrera E. (2002). Presence of Leishmania organisms in specific and non-specific skin lesions in HIV-infected individuals with visceral leishmaniasis. Int. J. Dermatol..

[15] Owens S.D., Oakley D.A., Marryott K., Hatchett W., Walton R., Nolan T.J., Newton A., Steurer F., Schantz P., Giger U. (2001). Transmission of visceral leishmaniasis through blood transfusions from infected English foxhounds to anemic dogs. J. Am. Vet. Med. Assoc..

[16] Boehme C.C., Hain U., Novosel A., Eichenlaub S., Fleischmann E., Löscher T. (2006). Congenital visceral leishmaniasis. Emerg. Infect. Dis..

[17] Low G., Edin M., Lond F., Cooke W., Irel F. (1926). A congenital case of Kala-azar. Lancet.

[18] Alvar J., Vélez I.D., Bern C., Herrero M., Desjeux P., Cano J., Jannin J., den Boer M. (2012). WHO Leishmaniasis Control Team Leishmaniasis worldwide and global estimates of its incidence. PLoS ONE.

[19] Burza S., Croft S.L., Boelaert M. (2018). Leishmaniasis. Lancet.

[20] Eddaikra N., Kherachi Djenad I., Benbetka S., Benikhlef R., Aït-Oudhia K., Moulti-Mati F., Oury B., Sereno D., Harrat Z. (2016). Development of a Murine Infection Model with Leishmania killicki, Responsible for Cutaneous Leishmaniosis in Algeria: Application in Pharmacology. BioMed Res. Int..

[21] Ostyn B., Gidwani K., Khanal B., Picado A., Chappuis F., Singh S.P., Rijal S., Sundar S., Boelaert M. (2011). Incidence of symptomatic and asymptomatic Leishmania donovani infections in high-endemic foci in India and Nepal: A prospective study. PLoS Negl. Trop. Dis..

[22] Silveira F.T., Lainson R., Crescente J.A., de Souza A.A.A., Campos M.B., Gomes C.M.C., Laurenti M.D., Corbett C.E.P. (2010). A prospective study on the dynamics of the clinical and immunological evolution of human Leishmania (L.) infantum chagasi infection in the Brazilian Amazon region. Trans. R. Soc. Trop. Med. Hyg..

[23] Travi B.L., Cordeiro-da-Silva A., Dantas-Torres F., Miró G. (2018). Canine visceral leishmaniasis: Diagnosis and management of the reservoir living among us. PLoS Negl. Trop. Dis..

[24] Roura X., Fondati A., Lubas G., Gradoni L., Maroli M., Oliva G., Paltrinieri S., Zatelli A., Zini E. (2013). Prognosis and monitoring of leishmaniasis in dogs: A working group report. Vet. J..

[25] Peña M.T., Naranjo C., Klauss G., Fondevila D., Leiva M., Roura X., Davidson M.G., Dubielzig R.R. (2008). Histopathological features of ocular leishmaniosis in the dog. J. Comp. Pathol..

[26] Pérez-Molina J.A., Norman F., López-Vélez R. (2012). Chagas disease in non-endemic countries: epidemiology, clinical presentation and treatment. Curr. Infect. Dis. Rep..

[27] Pérez-Molina J.A., Molina I. (2018). Chagas disease. Lancet (Lond. Engl.).

[28] Arnal A., Waleckx E., Rico-Chávez O., Herrera C., Dumonteil E. (2019). Estimating the current burden of Chagas disease in Mexico: A systematic review and meta-analysis of epidemiological surveys from 2006 to 2017. PLoS Negl. Trop. Dis..

[29] Silva-Dos-Santos D., Barreto-de-Albuquerque J., Guerra B., Moreira O.C., Berbert L.R., Ramos M.T., Mascarenhas B.A.S., Britto C., Morrot A., Serra Villa-Verde D.M. (2017). Unraveling Chagas disease transmission through the oral route: Gateways to Trypanosoma cruzi infection and target tissues. PLoS Negl. Trop. Dis..

[30] Breniere S.-F., Waleckx E., Aznar C., Telleria J., Tibayrenc M. (2017). American Trypanosomiasis (Chagas Disease), One Hundred Years of Research.

[31] Jardim E., Takayanagui O.M. (1994). Chagasic meningoencephalitis with detection of Trypanosoma cruzi in the cerebrospinal fluid of an immunodepressed patient. J. Trop. Med. Hyg..

[32] Diazgranados C.A., Saavedra-Trujillo C.H., Mantilla M., Valderrama S.L., Alquichire C., Franco-Paredes C. (2009). Chagasic encephalitis in HIV patients: Common presentation of an evolving epidemiological and clinical association. Lancet. Infect. Dis..

[33] Junqueira C., Caetano B., Bartholomeu D.C., Melo M.B., Ropert C., Rodrigues M.M., Gazzinelli R.T. (2010). The endless race between Trypanosoma cruzi and host immunity: Lessons for and beyond Chagas disease. Expert. Rev. Mol. Med..

[34] Teixeira A.R., Figueiredo F., Rezende Filho J., Macêdo V. (1983). Chagas’ disease: A clinical, parasitological, immunological, and pathological study in rabbits. Am. J. Trop. Med. Hyg..

[35] Bey E., Paucara Condori M.B., Gaget O., Solano P., Revollo S., Saussine C., Brenière S.F. (2018). Lower urinary tract dysfunction in chronic Chagas disease: Clinical and urodynamic presentation. World J. Urol..

[36] da Silva Junior G.B., Amélia Reis Jereissati A., Karoline Medina Neri A., da Costa Lino D.O., de Oliveira J.G.R., De Francesco Daher E. (2018). Neglected Tropical Diseases with an Impact on Kidney Function. IntechOpen.

[37] da Silva Junior G.B., Antunes V.V.H., Motta M., Barros E.J.G., Daher E.D.F. (2017). Chagas disease-associated kidney injury – A review. Nefrol. Latinoam..

[38] Riganti J., Maqueda M.G., Piñero M.C.B., Volonteri V.I., Galimberti R.L. (2012). Reactivation of Chagas’ disease: cutaneous manifestations in two immunosuppressed patients. Int. J. Dermatol..

[39] Franco J.R., Simarro P.P., Diarra A., Jannin J.G. (2014). Epidemiology of human African trypanosomiasis. Clin. Epidemiol..

[40] Dumas M., Bouteille B. (2002). Human African trypanosomiasis: Present and future treatment. Bull. Soc. Pathol. Exot..

[41] Dumas M., Bouteille B. (1997). Current status of trypanosomiasis. Med. Trop..

[42] Atouguia J.L.M., Kennedy P., Le D., Pge K. (2000). Neurological aspects of human African trypanosomiasis. Infectious Diseases of the Nervous System.

[43] Duggan A.J., Hutchinson M.P. (1966). Sleeping sickness in Europeans: A review of 109 cases. J. Trop. Med. Hyg..

[44] Collomb H., Bartoli D. (1967). The heart in human African trypanosomiasis caused by Trypanosoma gambiense. Bull. Soc. Pathol. Exot. Filiales.

[45] Collomb H., Bartoli D., Ayats H., Koate P. (1968). Cardiac disorders in human African trypanosomiasis due to Trypanosoma gambiense. Bull. Soc. Med. Afr. Noire Lang. Fr..

[46] Poltera A.A., Cox J.N., Owor R. (1977). Cardiac valvulitis in human African trypanosomiasis. East Afr. Med. J..

[47] Poltera A.A., Cox J.N., Owor R. (1975). African human trypanosomal pancarditis involving the conduting system and all valves. Pathol. Microbiol..

[48] Poltera A.A., Cox J.N. (1977). Pancarditis with valvulitis in endomyocardial fibrosis (=emf) and in human African trypanosomiasis (= hat). A comparative histological study of four Ugandan cases. Virchows Arch. A.

[49] Dwinger R.H., Rudin W., Moloo S.K., Murray M. (1988). Development of Trypanosoma congolense, T vivax and T brucei in the skin reaction induced in goats by infected Glossina morsitans centralis: A light and electron microscopical study. Res. Vet. Sci..

[50] McGovern T.W., Williams W., Fitzpatrick J.E., Cetron M.S., Hepburn B.C., Gentry R.H. (1995). Cutaneous manifestations of African trypanosomiasis. Arch. Dermatol..

[51] Tatibouet M.H., Gentilini M., Brucker G. (1982). Cutaneous lesions in human African trypanosomiasis. Sem. Hop..

[52] Capewell P., Cren-Travaillé C., Marchesi F., Johnston P., Clucas C., Benson R.A., Gorman T.-A., Calvo-Alvarez E., Crouzols A., Jouvion G. (2016). The skin is a significant but overlooked anatomical reservoir for vector-borne African trypanosomes. Elife.

[53] Caljon G., Van Reet N., De Trez C., Vermeersch M., Pérez-Morga D., Van Den Abbeele J. (2016). The Dermis as a Delivery Site of Trypanosoma brucei for Tsetse Flies. PLoS Pathog..

[54] Büscher P., Gonzatti M.I., Hébert L., Inoue N., Pascucci I., Schnaufer A., Suganuma K., Touratier L., Van Reet N. (2019). Equine trypanosomosis: Enigmas and diagnostic challenges. Parasit. Vectors.

[55] Raina A.K., Kumar R., Sridhar V.R., Singh R.P. (1985). Oral transmission of Trypanosoma evansi infection in dogs and mice. Vet. Parasitol..

[56] Sinha P.K., Mukherjee G.S., Das M.S., Lahiri R.K. (1971). Outbreak of trypanosomiasis evansi amongst tigers and jaguars in the zoological garden, Calcutta. Indian Vet. J..

[57] Gibson W. (2008). Molecular epidemiology of African trypanosomiasis: The contributions of David George Godfrey OBE to the biochemical characterization of trypanosomes. Parasite.

[58] Auty H., Torr S.J., Michoel T., Jayaraman S., Morrison L.J. (2015). Cattle trypanosomosis: The diversity of trypanosomes and implications for disease epidemiology and control. Rev. Sci. Tech..

[59] Desquesnes M., Dia M.L. (2003). Mechanical transmission of Trypanosoma congolense in cattle by the African tabanid Atylotus agrestis. Exp. Parasitol..

[60] Morrison L.J., Vezza L., Rowan T., Hope J.C. (2016). Animal African Trypanosomiasis: Time to Increase Focus on Clinically Relevant Parasite and Host Species. Trends Parasitol..

[61] Cox F.E.G. (1979). Pathogenesis of animal trypanosomiasis. Nature.

[62] Taylor K., Authié E.M.L. (2004). Pathogenesis of animal trypanosomiasis. The Trypanosomiases.

[63] Nasereddin A., Jaffe C.L. (2010). Rapid diagnosis of Old World Leishmaniasis by high-resolution melting analysis of the 7SL RNA gene. J. Clin. Microbiol..

[64] Higuera S.L., Guhl F., Ramírez J.D. (2013). Identification of Trypanosoma cruzi discrete typing units (DTUs) through the implementation of a high-resolution melting (HRM) genotyping assay. Parasit. Vectors.

[65] Mugasa C.M., Laurent T., Schoone G.J., Basiye F.L., Saad A.A., El Safi S., Kager P.A., Schallig H.D. (2010). Simplified molecular detection of Leishmania parasites in various clinical samples from patients with leishmaniasis. Parasit. Vectors.

[66] Deborggraeve S., Laurent T., Espinosa D., Van der Auwera G., Mbuchi M., Wasunna M., El-Safi S., Al-Basheer A.A., Arévalo J., Miranda-Verástegui C. (2008). A simplified and standardized polymerase chain reaction format for the diagnosis of leishmaniasis. J. Infect. Dis..

[67] Notomi T., Okayama H., Masubuchi H., Yonekawa T., Watanabe K., Amino N., Hase T. (2000). Loop-mediated isothermal amplification of DNA. Nucleic Acids Res..

[68] Khan M.G.M., Bhaskar K.R.H., Salam M.A., Akther T., Pluschke G., Mondal D. (2012). Diagnostic accuracy of loop-mediated isothermal amplification (LAMP) for detection of Leishmania DNA in buffy coat from visceral leishmaniasis patients. Parasit. Vectors.

[69] Kuboki N., Inoue N., Sakurai T., Di Cello F., Grab D.J., Suzuki H., Sugimoto C., Igarashi I. (2003). Loop-mediated isothermal amplification for detection of African trypanosomes. J. Clin. Microbiol..

[70] Fife E.H., Muschel L.H. (1959). Fluorescent-antibody technic for serodiagnosis of Trypanosoma cruzi infection. Proc. Soc. Exp. Biol. Med..

[71] Suter-Kopp V., Fricker F. (1972). Indirect immunofluorescence method in sleeping sickness (T. rhodesiense) with Trypanosoma brucei as antigen. Acta Trop..

[72] Lanotte G., Rioux J.A., Croset H., Vollhardt Y. (1975). Ecology of leishmaniasis in the south of France. 8. Complement to the epidemiological application of the immunofluorescence technic: Geometric and arithmetic mean titers in canine leighmaniasis. Ann. Parasitol. Hum. Comp..

[73] Connor R. The diagnosis, treatment and prevention of animal trypanosomiasis under field conditions. Proceedings of the FAO Panel of Experts.

[74] Magnus E., Vervoort T., Van Meirvenne N. (1978). A card-agglutination test with stained trypanosomes (C.A.T.T.) for the serological diagnosis of T. B. gambiense trypanosomiasis. Ann. Soc. Belg. Med. Trop..

[75] Da Silva A.S., da Krawczak F.S., Soares J.F., Klauck V., Pazinato R., Marcili A., Labruna M.B. (2016). Seroprevalence of Trypanosoma evansi infection in capybaras (Hydrochoerus hydrochaeris) from a nonendemic area in Brazil. J. Vet. Diagn. Investig..

[76] Attar Z.J., Chance M.L., El-Safi S., Carney J., Azazy A., El-Hadi M., Dourado C., Hommel M. (2001). Latex agglutination test for the detection of urinary antigens in visceral leishmaniasis. Acta Trop..

[77] Noyes H.A., Reyburn H., Bailey J.W., Smith D. (1998). A nested-PCR-based schizodeme method for identifying Leishmania kinetoplast minicircle classes directly from clinical samples and its application to the study of the epidemiology of Leishmania tropica in Pakistan. J. Clin. Microbiol..

[78] Desquesnes M., Dávila A.M.R. (2002). Applications of PCR-based tools for detection and identification of animal trypanosomes: A review and perspectives. Vet. Parasitol..

[79] Gibson W. (2009). Species-specific probes for the identification of the African tsetse-transmitted trypanosomes. Parasitology.

[80] Mugasa C.M., Laurent T., Schoone G.J., Kager P.A., Lubega G.W., Schallig H.D.F.H. (2009). Nucleic acid sequence-based amplification with oligochromatography for detection of Trypanosoma brucei in clinical samples. J. Clin. Microbiol..

[81] Mugasa C.M., Deborggraeve S., Schoone G.J., Laurent T., Leeflang M.M., Ekangu R.A., El Safi S., Saad A.A., Basiye F.L., De Doncker S. (2010). Accordance and concordance of PCR and NASBA followed by oligochromatography for the molecular diagnosis of Trypanosoma brucei and Leishmania. Trop. Med. Int. Health.

[82] Masake R.A., Njuguna J.T., Brown C.C., Majiwa P.A.O. (2002). The application of PCR-ELISA to the detection of Trypanosoma brucei and T. vivax infections in livestock. Vet. Parasitol..

[83] De Doncker S., Hutse V., Abdellati S., Rijal S., Singh Karki B.M., Decuypere S., Jacquet D., Le Ray D., Boelaert M., Koirala S. (2005). A new PCR-ELISA for diagnosis of visceral leishmaniasis in blood of HIV-negative subjects. Trans. R. Soc. Trop. Med. Hyg..

[84] Cabrera L., De Witte J., Victor B., Vermeiren L., Zimic M., Brandt J., Geysen D. (2009). Specific detection and identification of African trypanosomes in bovine peripheral blood by means of a PCR-ELISA assay. Vet. Parasitol..

[85] Delespaux V., Ayral F., Geysen D., Geerts S. (2003). PCR-RFLP using Ssu-rDNA amplification: Applicability for the diagnosis of mixed infections with different trypanosome species in cattle. Vet. Parasitol..

[86] Freitas J.M., Lages-Silva E., Crema E., Pena S.D.J., Macedo A.M. (2005). Real time PCR strategy for the identification of major lineages of Trypanosoma cruzi directly in chronically infected human tissues. Int. J. Parasitol..

[87] Sá A.R.N., Kimoto K.Y., Steindel M., Grisard E.C., Gomes M.L. (2018). Limit of detection of PCR/RFLP analysis of cytochrome oxidase II for the identification of genetic groups of Trypanosoma cruzi and Trypanosoma rangeli in biological material from vertebrate hosts. Parasitol. Res..

[88] Akhoundi M., Downing T., Votýpka J., Kuhls K., Lukeš J., Cannet A., Ravel C., Marty P., Delaunay P., Kasbari M. (2017). Leishmania infections: Molecular targets and diagnosis. Mol. Aspects Med..

[89] Tilley A., Hide G. (2001). Characterization of Trypanosoma brucei stocks using PCR-RFLP analysis of ribosomal internal transcribed spacers (IRT). Ann. Trop. Med. Parasitol..

[90] Melville S.E. (1997). Parasite genome analysis. Genome research in Trypanosoma brucei: Chromosome size polymorphism and its relevance to genome mapping and analysis. Trans. R. Soc. Trop. Med. Hyg..

[91] Henriksson J., Aslund L., Macina R.A., de Cazzulo B.M.F., Cazzulo J.J., Frasch A.C., Pettersson U. (1990). Chromosomal localization of seven cloned antigen genes provides evidence of diploidy and further demonstration of karyotype variability in Trypanosoma cruzi. Mol. Biochem. Parasitol..

[92] Besuschio S.A., Llano Murcia M., Benatar A.F., Monnerat S., Cruz I., Picado A., Curto M.d.L.Á., Kubota Y., Wehrendt D.P., Pavia P. (2017). Analytical sensitivity and specificity of a loop-mediated isothermal amplification (LAMP) kit prototype for detection of Trypanosoma cruzi DNA in human blood samples. PLoS Negl. Trop. Dis..

[93] Frean J., Sieling W., Pahad H., Shoul E., Blumberg L. (2018). Clinical management of East African trypanosomiasis in South Africa: Lessons learned. Int. J. Infect. Dis..

[94] Brumpt E. (1914). Le xénodiagnostic. Application au diagnostic de quelques infections parasitaires et en particulier à la Trypanosomose de Chagas. Bull. Soc. Pathol. Exot..

[95] Sadlova J., Seblova V., Votypka J., Warburg A., Volf P. (2015). Xenodiagnosis of Leishmania donovani in BALB/c mice using Phlebotomus orientalis: A new laboratory model. Parasit. Vectors.

[96] Saavedra M., Zulantay I., Apt W., Castillo J., Araya E., Martínez G., Rodríguez J. (2016). Quantification by real-time PCR of Trypanosoma cruzi DNA in samples of Triatoma infestans used in xenodiagnosis of chronic Chagas disease patients. Parasit. Vectors.

[97] Frezil J.L. (1971). Application of xenodiagnosis in the detection of T. gambiense trypanosomiasis in immunologically suspect patients. Bull. Soc. Pathol. Exot. Filiales.

[98] Guedes D.C., Minozzo J.C., Pasquali A.K.S., Faulds C., Soccol C.R., Thomaz-Soccol V. (2017). New strategy to improve quality control of Montenegro skin test at the production level. Rev. Soc. Bras. Med. Trop..

[99] Sarkari B., Hatam G.R., Mikaeili F., Sadeghi H., Ebrahimi S. (2008). A comparative study of antigen and antibody detection in visceral leishmaniasis using serum and urine-based ELISA. Trop. Biomed..

[100] Sguassero Y., Roberts K.N., Harvey G.B., Comandé D., Ciapponi A., Cuesta C.B., Aguiar C., de Castro A.M., Danesi E., de Andrade A.L. (2018). Course of serological tests in treated subjects with chronic Trypanosoma cruzi infection: A systematic review and meta-analysis of individual participant data. Int. J. Infect. Dis..

[101] Berrizbeitia M., Ndao M., Bubis J., Gottschalk M., Ache A., Lacouture S., Medina M., Ward B.J. (2006). Purified Excreted-Secreted Antigens from Trypanosoma cruzi Trypomastigotes as Tools for Diagnosis of Chagas’ Disease. J. Clin. Microbiol..

[102] Nantulya V.M., Lindqvist K.J. (1989). Antigen-detection enzyme immunoassays for the diagnosis of Trypanosoma vivax, T. congolense and T. brucei infections in cattle. Trop. Med. Parasitol..

[103] Nozais J.P., Giordano C., Doucet J., Bertrand E. (1975). Value of indirect immunofluorescence in the diagnosis of “Trypanosoma gambiense” trypanosomiasis. 46 cases (author’s translation). Bull. Soc. Pathol. Exot. Filiales.

[104] Frezil J., Louembet M., Alary J. (1978). L’antigène “Trypanosoma gambiense” dans la réaction d’immunofluorescence indirecte. Cah. ORSTOM.

[105] Singh D.P., Goyal R.K., Singh R.K., Sundar S., Mohapatra T.M. (2010). In search of an ideal test for diagnosis and prognosis of kala-azar. J. Health Popul. Nutr..

[106] Chappuis F., Rijal S., Soto A., Menten J., Boelaert M. (2006). A meta-analysis of the diagnostic performance of the direct agglutination test and rK39 dipstick for visceral leishmaniasis. BMJ.

[107] Büscher P., Gilleman Q., Lejon V. (2013). Rapid diagnostic test for sleeping sickness. N. Engl. J. Med..

[108] Lejon V., Büscher P., Nzoumbou-Boko R., Bossard G., Jamonneau V., Bucheton B., Truc P., Lemesre J.-L., Solano P., Vincendeau P. (2019). The separation of trypanosomes from blood by anion exchange chromatography: From Sheila Lanham’s discovery 50 years ago to a gold standard for sleeping sickness diagnosis. PLoS Negl. Trop. Dis..

[109] Mirzaei A., Ahmadipour F., Cannet A., Marty P., Delaunay P., Perrin P., Dorkeld F., Sereno D., Akhoundi M. (2018). Immunodetection and molecular determination of visceral and cutaneous Leishmania infection using patients’ urine. Infect. Genet. Evol..

[110] Riera C., Verges M., Iniesta L., Fisa R., Gállego M., Tebar S., Portús M. (2012). Identification of a Western blot pattern for the specific diagnosis of Trypanosoma cruzi infection in human sera. Am. J. Trop. Med. Hyg..

[111] Lachaud L., Fernández-Arévalo A., Normand A.-C., Lami P., Nabet C., Donnadieu J.L., Piarroux M., Djenad F., Cassagne C., Ravel C. (2017). Identification of Leishmania by Matrix-Assisted Laser Desorption Ionization-Time of Flight (MALDI-TOF) Mass Spectrometry Using a Free Web-Based Application and a Dedicated Mass-Spectral Library. J. Clin. Microbiol..

[112] Avila C.C., Almeida F.G., Palmisano G. (2016). Direct identification of trypanosomatids by matrix-assisted laser desorption ionization-time of flight mass spectrometry (DIT MALDI-TOF MS). J. Mass Spectrom..

[113] Sakthianandeswaren A., Foote S.J., Handman E. (2009). The role of host genetics in leishmaniasis. Trends Parasitol..

[114] Geerts S., Osaer S., Goossens B., Faye D. (2009). Trypanotolerance in small ruminants of sub-Saharan Africa. Trends Parasitol..

[115] Sternberg J.M., Maclean L. (2010). A spectrum of disease in human African trypanosomiasis: The host and parasite genetics of virulence. Parasitology.

[116] Mangano V.D., Modiano D. (2014). Host genetics and parasitic infections. Clin. Microbiol. Infect..

[117] Marsden P. (1966). Survival of trypanosoma cruzi in human saliva and urine. Trans. R. Soc. Trop. Med. Hyg..

[118] Howard M.K., Pharoah M.M., Ashall F., Miles M.A. (1991). Human urine stimulates growth of Leishmania in vitro. Trans. R. Soc. Trop. Med. Hyg..

[119] Mebrahtu Y.B., Hendricks L.D., Oster C.N., Lawyer P.G., Perkins P.V., Pamba H., Koech D., Roberts C.R. (1993). Leishmania donovani parasites in the nasal secretions, tonsillopharyngeal mucosa, and urine centrifugates of visceral leishmaniasis patients in Kenya. Am. J. Trop. Med. Hyg..

[120] da Costa Lima M.S., Hartkopf A.C.L., de Souza Tsujisaki R.A., Oshiro E.T., Shapiro J.T., de Fatima Cepa Matos M., Cavalheiros Dorval M.E. (2018). Isolation and molecular characterization of Leishmania infantum in urine from patients with visceral leishmaniasis in Brazil. Acta Trop..

[121] Fisa R., Riera C., López-Chejade P., Molina I., Gállego M., Falcó V., Ribera E., Portús M. (2008). Leishmania infantum DNA detection in urine from patients with visceral leishmaniasis and after treatment control. Am. J. Trop. Med. Hyg..

[122] Riera C., Valladares J.E. (1996). Viable Leishmania infantum in urine and semen in experimentally infected dogs. Parasitol. Today.

[123] Caravaca F., Muñoz A., Pizarro J.L., de Santamaría J.S., Fernandez-Alonso J. (1991). Acute renal failure in visceral leishmaniasis. Am. J. Nephrol..

[124] de Alcântara C.C.S., Santana L.R.L., Evangelista P.D., Teixeira A.C., da Silva Junior G.B., Daher E.D.F. (2018). Renal dysfunction in Leishmaniasis and Chagas disease coinfection: A case report. Rev. Inst. Med. Trop. Sao Paulo.

[125] Koutinas A.F., Koutinas C.K. (2014). Pathologic mechanisms underlying the clinical findings in canine leishmaniasis due to Leishmania infantum/chagasi. Vet. Pathol..

[126] Pennisi M.-G., Cardoso L., Baneth G., Bourdeau P., Koutinas A., Miró G., Oliva G., Solano-Gallego L. (2015). LeishVet update and recommendations on feline leishmaniosis. Parasit. Vectors.

[127] Clementi A., Battaglia G., Floris M., Castellino P., Ronco C., Cruz D.N. (2011). Renal involvement in leishmaniasis: A review of the literature. NDT Plus.

[128] Bezerra G.S.N., Barbosa W.L., da Silva E.D., Leal N.C., de Medeiros Z.M. (2019). Urine as a promising sample for Leishmania DNA extraction in the diagnosis of visceral leishmaniasis—A review. Braz. J. Infect. Dis..

[129] Ben-Abid M., Galaï Y., Habboul Z., Ben-Abdelaziz R., Ben-Sghaier I., Aoun K., Bouratbine A. (2017). Diagnosis of Mediterranean visceral leishmaniasis by detection of Leishmania -related antigen in urine and oral fluid samples. Acta Trop..

[130] Veland N., Espinosa D., Valencia B.M., Ramos A.P., Calderon F., Arevalo J., Low D.E., Llanos-Cuentas A., Boggild A.K. (2011). Polymerase chain reaction detection of Leishmania kDNA from the urine of Peruvian patients with cutaneous and mucocutaneous leishmaniasis. Am. J. Trop. Med. Hyg..

[131] Solano-Gallego L., Rodriguez-Cortes A., Trotta M., Zampieron C., Razia L., Furlanello T., Caldin M., Roura X., Alberola J. (2007). Detection of Leishmania infantum DNA by fret-based real-time PCR in urine from dogs with natural clinical leishmaniosis. Vet. Parasitol..

[132] Manna L., Reale S., Picillo E., Vitale F., Gravino A.E. (2008). Urine sampling for real-time polymerase chain reaction based diagnosis of canine leishmaniasis. J. Vet. Diagn. Investig..

[133] Pessoa-e-Silva R., Mendonça Trajano-Silva L.A., Lopes da Silva M.A., da Cunha Gonçalves-de-Albuquerque S., de Goes T.C., Silva de Morais R.C., Lopes de Melo F., de Paiva-Cavalcanti M. (2016). Evaluation of urine for Leishmania infantum DNA detection by real-time quantitative PCR. J. Microbiol. Methods.

[134] Phumee A., Kraivichian K., Chusri S., Noppakun N., Vibhagool A., Sanprasert V., Tampanya V., Wilde H., Siriyasatien P. (2013). Detection of Leishmania siamensis DNA in saliva by polymerase chain reaction. Am. J. Trop. Med. Hyg..

[135] Franceschi A., Merildi V., Guidi G., Mancianti F. (2007). Occurrence of Leishmania DNA in urines of dogs naturally infected with leishmaniasis. Vet. Res. Commun..

[136] Burrows W., Havens I. (1948). 1. Studies on immunity to Asiatic cholera; the absorption of immuneglobulin from the bowel and its excretion in the urine and feces of experimental animals and human volunteers. J. Infect. Dis..

[137] Hanson L., Tan E. (1965). Characterization of antibodies in human urine. J. Clin. Investig..

[138] Kohanteb J., Ardehali S.M., Rezai H.R. (1987). Detection of Leishmania donovani soluble antigen and antibody in the urine of visceral leishmaniasis patients. Trans. R. Soc. Trop. Med. Hyg..

[139] Islam M.Z., Itoh M., Shamsuzzaman S.M., Mirza R., Matin F., Ahmed I., Shamsuzzaman Choudhury A.K.M., Hossain M.A., Qiu X.-G., Begam N. (2002). Diagnosis of visceral leishmaniasis by enzyme-linked immunosorbent assay using urine samples. Clin. Diagn. Lab. Immunol..

[140] Islam M.Z., Itoh M., Mirza R., Ahmed I., Ekram A.R.M.S., Sarder A.H., Shamsuzzaman S.M., Hashiguchi Y., Kimura E. (2004). Direct agglutination test with urine samples for the diagnosis of visceral leishmaniasis. Am. J. Trop. Med. Hyg..

[141] Islam M.Z., Itoh M., Takagi H., Islam A.U., Ekram A.R.M.S., Rahman A., Takesue A., Hashiguchi Y., Kimura E. (2008). Enzyme-linked immunosorbent assay to detect urinary antibody against recombinant rKRP42 antigen made from Leishmania donovani for the diagnosis of visceral leishmaniasis. Am. J. Trop. Med. Hyg..

[142] Hatam G., Mikaeili F., Sadjjadi S., Sarkari B. (2007). Direct Agglutination Test and Enzyme Linked Immunosorbent Assay with Urine Samples for the Diagnosis of Visceral Leishmaniasis. Iran. J. Parasitol..

[143] Khan M.G.M., Alam M.S., Podder M.P., Itoh M., Jamil K.M., Haque R., Wagatsuma Y. (2010). Evaluation of rK-39 strip test using urine for diagnosis of visceral leishmaniasis in an endemic area in Bangladesh. Parasit. Vectors.

[144] Singh D., Pandey K., Das V.N.R., Das S., Verma N., Ranjan A., Lal S.C., Topno K.R., Singh S.K., Verma R.B. (2013). Evaluation of rK-39 strip test using urine for diagnosis of visceral leishmaniasis in an endemic region of India. Am. J. Trop. Med. Hyg..

[145] Chakravarty J., Kumar S., Kumar R., Gautam S., Rai M., Sundar S. (2011). Evaluation of rk39 immunochromatographic test with urine for diagnosis of visceral leishmaniasis. Trans. R. Soc. Trop. Med. Hyg..

[146] Goswami R.P., Goswami R.P., Das S., Ray Y., Rahman M. (2012). Testing urine samples with rK39 strip as the simplest non-invasive field diagnosis for visceral leishmaniasis: An early report from eastern India. J. Postgrad. Med..

[147] Ejazi S.A., Bhattacharya P., Bakhteyar M.A.K., Mumtaz A.A., Pandey K., Das V.N.R., Das P., Rahaman M., Goswami R.P., Ali N. (2016). Noninvasive Diagnosis of Visceral Leishmaniasis: Development and Evaluation of Two Urine-Based Immunoassays for Detection of Leishmania donovani Infection in India. PLoS Negl. Trop. Dis..

[148] Diro E., Techane Y., Tefera T., Assefa Y., Kebede T., Genetu A., Kebede Y., Tesfaye A., Ergicho B., Gebre-Yohannes A. (2007). Field evaluation of FD-DAT, rK39 dipstick and KATEX (urine latex agglutination) for diagnosis of visceral leishmaniasis in northwest Ethiopia. Trans. R. Soc. Trop. Med. Hyg..

[149] Singh D., Pandey K., Das V.N.R., Das S., Kumar S., Topno R.K., Das P. (2009). Novel noninvasive method for diagnosis of visceral leishmaniasis by rK39 testing of sputum samples. J. Clin. Microbiol..

[150] Mohapatra S., Samantaray J.C., Ghosh A. (2016). A Comparative Study of Serum, Urine and Saliva Using rk39 Strip for the Diagnosis of Visceral Leishmaniasis. J. Arthropod Borne. Dis..

[151] Islam M.Z., Itoh M., Ul Islam M.A., Saifuddin Ekram A.R.M., Rahman M.A., Takagi H., Takesue A., Hashiguchi Y., Kimura E. (2012). ELISA with recombinant rKRP42 antigen using urine samples: A tool for predicting clinical visceral leishmaniasis cases and its outbreak. Am. J. Trop. Med. Hyg..

[152] De Colmenares M., Portus M., Riera C., Gallego M., Aisa M.J., Torras S., Munoz C. (1995). Short report: Detection of 72-75-kD and 123-kD fractions of Leishmania antigen in urine of patients with visceral leishmaniasis. Am. J. Trop. Med. Hyg..

[153] Zaragoza C., Barrera R., Centeno F., Tapia J.A., Durán E., González M., Mañé M.C. (2003). SDS-PAGE and Western blot of urinary proteins in dogs with leishmaniasis. Vet. Res..

[154] de Lira N.M.S. (2008). Avaliação Física, Química, Microbiológica e Pesquisa de Anticorpos Anti-IgG em Urina de Cães (Canis familiaris) Linnaeus, 1785) Naturalmente Infectados por Leishmania (Leishmania) Chagasi (Cunha & Chagas, 1937). Master’s Thesis.

[155] Solano-Gallego L., Rodríguez A., Iniesta L., Arboix M., Portús M., Alberola J. (2003). Detection of anti-Leishmania immunoglobulin G antibodies in urine specimens of dogs with leishmaniasis. Clin. Diagn. Lab. Immunol..

[156] Todolí F., Solano-Gallego L., Ojeda A., Quintana J., Lloret A., Roura X., Alberola J., Rodríguez-Cortés A. (2009). Anti-Leishmania IgA in urine samples from dogs with clinical leishmaniasis. Vet. Parasitol..

[157] Azazy A.A., Chance M.L., Devaney E. (1997). A time-course study of circulating antigen and parasite-specific antibody in cotton rats infected with Leishmania donovani. Ann. Trop. Med. Parasitol..

[158] Kashino S.S., Abeijon C., Qin L., Kanunfre K.A., Kubrusly F.S., Silva F.O., Costa D.L., Campos D., Costa C.H.N., Raw I. (2012). Identification of Leishmania infantum chagasi proteins in urine of patients with visceral leishmaniasis: A promising antigen discovery approach of vaccine candidates. Parasite Immunol..

[159] Abeijon C., Kashino S.S., Silva F.O., Costa D.L., Fujiwara R.T., Costa C.H.N., Campos-Neto A. (2012). Identification and Diagnostic Utility of Leishmania infantum Proteins Found in Urine Samples from Patients with Visceral Leishmaniasis. Clin. Vaccine Immunol..

[160] Ferlizza E. (2015). Urine Proteome in Animals of Veterinary Interest: Species Comparison and New Biomarkers of Nephropathy. Master’s Thesis.

[161] Abeijon C., Alves F., Monnerat S., Wasunna M., Mbui J., Viana A.G., Bueno L.L., Siqueira W.F., Carvalho S.G., Agrawal N. (2019). Development of a multiplexed assay for the detection of Leishmania donovani/Leishmania infantum protein biomarkers in the urine of patients with visceral leishmaniasis. J. Clin. Microbiol..

[162] Sarkari B., Chance M., Hommel M. (2002). Antigenuria in visceral leishmaniasis: Detection and partial characterisation of a carbohydrate antigen. Acta Trop..

[163] Vallur A.C., Tutterrow Y.L., Mohamath R., Pattabhi S., Hailu A., Abdoun A.O., Ahmed A.E., Mukhtar M., Salam M.A., Almeida M.L. (2015). Development and comparative evaluation of two antigen detection tests for Visceral Leishmaniasis. BMC Infect. Dis..

[164] El-Safi S.H., Abdel-Haleem A., Hammad A., El-Basha I., Omer A., Kareem H.G., Boelaert M., Chance M., Hommel M. (2003). Field evaluation of latex agglutination test for detecting urinary antigens in visceral leishmaniasis in Sudan. East. Mediterr. Health J..

[165] Rijal S., Boelaert M., Regmi S., Karki B.M.S., Jacquet D., Singh R., Chance M.L., Chappuis F., Hommel M., Desjeux P. (2004). Evaluation of a urinary antigen-based latex agglutination test in the diagnosis of kala-azar in eastern Nepal. Trop. Med. Int. Health.

[166] Vilaplana C., Blanco S., Domínguez J., Giménez M., Ausina V., TUral C., Muñoz C. (2004). Noninvasive method for diagnosis of visceral leishmaniasis by a latex agglutination test for detection of antigens in urine samples. J. Clin. Microbiol..

[167] Sundar S., Agrawal S., Pai K., Chance M., Hommel M. (2005). Detection of leishmanial antigen in the urine of patients with visceral leishmaniasis by a latex agglutination test. Am. J. Trop. Med. Hyg..

[168] Motazedian M.H.M., Fakhar M., Motazedian M.H.M., Hatam G., Mikaeili F. (2008). A urine-based polymerase chain reaction method for the diagnosis of visceral leishmaniasis in immunocompetent patients. Diagn. Microbiol. Infect. Dis..

[169] Riera C., Fisa R., Lopez P., Ribera E., Carrió J., Falcó V., Molina I., Gállego M., Portús M. (2004). Evaluation of a latex agglutination test (KAtex) for detection of Leishmania antigen in urine of patients with HIV-Leishmania coinfection: Value in diagnosis and post-treatment follow-up. Eur. J. Clin. Microbiol. Infect. Dis..

[170] Boelaert M., Verdonck K., Menten J., Sunyoto T., van Griensven J., Chappuis F., Rijal S. (2014). Rapid tests for the diagnosis of visceral leishmaniasis in patients with suspected disease. Cochrane Database Syst. Rev..

[171] van Griensven J., Mengesha B., Mekonnen T., Fikre H., Takele Y., Adem E., Mohammed R., Ritmeijer K., Vogt F., Adriaensen W. (2018). Leishmania Antigenuria to Predict Initial Treatment Failure and Relapse in Visceral Leishmaniasis/HIV Coinfected Patients: An Exploratory Study Nested Within a Clinical Trial in Ethiopia. Front. Cell. Infect. Microbiol..

[172] Asfaram S., Hosseini Teshnizi S., Fakhar M., Banimostafavi E.S., Soosaraei M. (2018). Is urine a reliable clinical sample for the diagnosis of human visceral leishmaniasis? A systematic review and meta-analysis. Parasitol. Int..

[173] Ngotho M., Kagira J.M., Gachie B.M., Karanja S.M., Waema M.W., Maranga D.N., Maina N.W. (2015). Loop Mediated Isothermal Amplification for Detection of Trypanosoma brucei gambiense in Urine and Saliva Samples in Nonhuman Primate Model. BioMed Res. Int..

[174] Itazi O.K., Enyaru J.C. (1973). The nature of proteins excreted in the urine of rabbits infected with T. brucei subgroup organisms. Trans. R. Soc. Trop. Med. Hyg..

[175] Boreham P.F.L., Facer C.A. (1977). Fibrinogen and fibrinogen/fibrin degradation products in the urine of rabbits infected with Trypanosoma (Trypanozoon) brucei. Z. Parasitenkd..

[176] Hall J., Seed J. (1981). Quantification of aromatic amino acid catabolites in urine of mice acutely infected with Trypanosoma brucei gambiense. Comp. Biochem. Physiol..

[177] Seed J.R., Hall J.E., Sechelski J. (1982). Phenylalanine metabolism in Microtus montanus chronically infected with Trypanosoma brucei gambiense. Comp. Biochem. Physiol..

[178] Hall J.E., Seed J.R., Sechelski J.B. (1985). Multiple alpha-keto aciduria in Microtus montanus chronically infected with Trypanosoma brucei gambiense. Comp. Biochem. Physiol..

[179] Hall J.E., Seed J.R. (1984). Increased urinary excretion of aromatic amino acid catabolites by Microtus montanus chronically infected with Trypanosoma brucei gambiense. Comp. Biochem. Physiol..

[180] El Sawalhy A., Seed J.R., Hall J.E., El Attar H. (1998). Increased excretion of aromatic amino acid catabolites in animals infected with Trypanosoma brucei evansi. J. Parasitol..

[181] Nowicki C., Cazzulo J.J. (2008). Aromatic amino acid catabolism in trypanosomatids. Comp. Biochem. Physiol. Part A Mol. Integr. Physiol..

[182] Bonnet J., Garcia C., Leger T., Couquet M.-P., Vignoles P., Vatunga G., Ndung’u J., Boudot C., Bisser S., Courtioux B. (2019). Proteome characterization in various biological fluids of Trypanosoma brucei gambiense-infected subjects. J. Proteom..

[183] Simaren J., Ogunnaike M. (1989). Urinary biochemical changes, histopathologic effect of kidney damage observed in rats infected with Trypanosoma b. brucei. Trop. Med. Parasitol..

[184] Seed J.R. (1969). Trypanosoma gambiense and T. lewisi: Increased vascular permeability and skin lesions in rabbits. Exp. Parasitol..

[185] Monroy F.P., Dusanic D.G. (2000). The kidney form of Trypanosoma musculi: A distinct stage in the life cycle?. Parasitol. Today.

[186] Arias L.F., Duque E., Ocampo C., Henao J., Zuluaga G., Varela G., Carvajal J., Duque J., Robledo-Villegas M., Arbeláez M. (2006). Detection of amastigotes of Trypanosoma cruzi in a kidney graft with acute dysfunction. Transplant. Proc..

[187] González G., Sunnemark D., Orn A., Grönvik K.O. (1996). Detection of cruzipain, the major cysteine proteinase from Trypanosoma cruzi and its C-terminal extension in biological fluids during experimental infection in mice. Scand. J. Immunol..

[188] Yauri V., Castro-Sesquen Y.E., Verastegui M., Angulo N., Recuenco F., Cabello I., Malaga E., Bern C., Gavidia C.M., Gilman R.H. (2016). Domestic Pig (Sus scrofa) as an Animal Model for Experimental Trypanosoma cruzi Infection. Am. J. Trop. Med. Hyg..

[189] Castro-Sesquen Y.E., Gilman R.H., Yauri V., Cok J., Angulo N., Escalante H., Bern C. (2013). Detection of soluble antigen and DNA of Trypanosoma cruzi in urine is independent of renal injury in the guinea pig model. PLoS ONE.

[190] Freilij H.L., Corral R.S., Katzin A.M., Grinstein S. (1987). Antigenuria in infants with acute and congenital Chagas’ disease. J. Clin. Microbiol..

[191] Corral R., Freilij H., Montemayor A., Grinstein S. (1984). Trypanosoma-cruzi antigens in urine from patients with chagas-disease. IRCS Med. Sci..

[192] Corral R.S., Altcheh J., Alexandre S.R., Grinstein S., Freilij H., Katzin A.M. (1996). Detection and characterization of antigens in urine of patients with acute, congenital, and chronic Chagas’ disease. J. Clin. Microbiol..

[193] Katzin A.M., Marcipar A., Freilij H., Corral R., Yanovsky J.F. (1989). Rapid determination of Trypanosoma cruzi urinary antigens in human chronic Chagas disease by agglutination test. Exp. Parasitol..

[194] Umezawa E.S., Shikanai-Yasuda M.A., da Silveira J.F., Cotrim P.C., Paranhos G., Katzin A.M. (1993). Trypanosoma cruzi: Detection of a circulating antigen in urine of chagasic patients sharing common epitopes with an immunodominant repetitive antigen. Exp. Parasitol..

[195] Corral R.S., Orn A., Freilij H.L., Bergman T., Grinstein S. (1989). Purification and characterization of an 80-kilodalton Trypanosoma cruzi urinary antigen. J. Clin. Microbiol..

[196] Corral R.S., Bertot G.M., Petray P.B., Altcheh J.M., Singh M., Orn A., Rapoport M.F., Grinstein S. (1995). An iron-binding Trypanosoma cruzi urinary antigen. Parasite.

[197] Málaga-Machaca E.S., Romero-Ramirez A., Gilman R.H., Astupiña-Figueroa S., Angulo N., Florentini A., Lovon-Luque C.J., Gonza R.A., Del Carpio-Sanz A., Cabello I. (2017). Polyclonal antibodies for the detection of Trypanosoma cruzi circulating antigens. PLoS Negl. Trop. Dis..

[198] Luchini A., Geho D.H., Bishop B., Tran D., Xia C., Dufour R.L., Jones C.D., Espina V., Patanarut A., Zhou W. (2008). Smart hydrogel particles: Biomarker harvesting: One-step affinity purification, size exclusion, and protection against degradation. Nano Lett..

[199] Douglas T.A., Tamburro D., Fredolini C., Espina B.H., Lepene B.S., Ilag L., Espina V., Petricoin E.F., Liotta L.A., Luchini A. (2011). The use of hydrogel microparticles to sequester and concentrate bacterial antigens in a urine test for Lyme disease. Biomaterials.

[200] Castro-Sesquen Y.E., Gilman R.H., Galdos-Cardenas G., Ferrufino L., Sánchez G., Valencia Ayala E., Liotta L., Bern C., Luchini A. (2014). Working Group on Chagas Disease in Bolivia and Peru Use of a novel chagas urine nanoparticle test (chunap) for diagnosis of congenital chagas disease. PLoS Negl. Trop. Dis..

[201] Castro-Sesquen Y.E., Gilman R.H., Mejia C., Clark D.E., Choi J., Reimer-McAtee M.J., Castro R., Valencia-Ayala E., Flores J., Bowman N. (2016). Use of a Chagas Urine Nanoparticle Test (Chunap) to Correlate with Parasitemia Levels in T. cruzi/HIV Co-infected Patients. PLoS Negl. Trop. Dis..

[202] Mackie F. (1914). Note on some bodies of unknown nature found in faeces of kala-azar patients. Indian J. Med. Res..

[203] Shortt H., Smith R., D’Silva M., Swaminath C. (1929). Leishmania donovani in human faeces in indian kala-azar. Indian J. Med. Res..

[204] Dollahon N.R., Janovy J. (1971). Insect flagellates from feces and gut contents of four genera of lizards. J. Parasitol..

[205] Nery G., Meneses I.D.S., Trueb I., Larangeira D.F., Barrouin-Melo S.M. (2015). Ocorrência de Leishmania infantum em fezes de cão. Arq. Bras. Med. Vet. Zootec..

[206] Hamad I., Forestier C.-L., Peeters M., Delaporte E., Raoult D., Bittar F. (2015). Wild gorillas as a potential reservoir of Leishmania major. J. Infect. Dis..

[207] Hamad I., Forestier C.-L., Greub G., Jaton K., Raoult D., Bittar F. (2015). Reply to Bastien et al. J. Infect. Dis..

[208] Bastien P., Volf P., Depaquit J., Dondji B., Gallego M., Gangneux J.-P., Izri A., Marty P., Piarroux R., Pratlong F. (2015). Comments on Leishmania major in Gorilla Feces. J. Infect. Dis..

[209] Votýpka J., Pafčo B., Modrý D., Mbohli D., Tagg N., Petrželková K.J. (2018). An unexpected diversity of trypanosomatids in fecal samples of great apes. Int. J. Parasitol. Parasites Wildl..

[210] Santiago-Rodriguez T.M., Fornaciari G., Luciani S., Dowd S.E., Toranzos G.A., Marota I., Cano R.J. (2016). Taxonomic and predicted metabolic profiles of the human gut microbiome in pre-Columbian mummies. FEMS Microbiol. Ecol..

[211] Sereno D., Dorkeld F., Akhoundi M., Perrin P. (2018). Pathogen Species Identification from Metagenomes in Ancient Remains: The Challenge of Identifying Human Pathogenic Species of Trypanosomatidae via Bioinformatic Tools. Genes.

[212] Sereno D., Akhoundi M., Dorkeld F., Oury B., Momen H., Perrin P. (2017). What pre-Columbian mummies could teach us about South American leishmaniases?. Pathog. Dis..

[213] Jirků M., Votýpka J., Petrželková K.J., Jirků-Pomajbíková K., Kriegová E., Vodička R., Lankester F., Leendertz S.A.J., Wittig R.M., Boesch C. (2015). Wild chimpanzees are infected by Trypanosoma brucei. Int. J. Parasitol. Parasites Wildl..

[214] Fernandes A., Iñiguez A.M., Lima V.S., de Souza S.M.F.M., Ferreira L.F., Vicente A.C.P., Jansen A.M. (2008). Pre-Columbian Chagas disease in Brazil: Trypanosoma cruzi I in the archaeological remains of a human in Peruaçu Valley, Minas Gerais, Brazil. Mem. Inst. Oswaldo Cruz.

[215] Araújo A., Jansen A.M., Reinhard K., Ferreira L.F. (2009). Paleoparasitology of Chagas disease—A review. Mem. Inst. Oswaldo Cruz.

[216] Franco D.J., Vago A.R., Chiari E., Meira F.C.A., Galvão L.M.C., Machado C.R.S. (2003). Trypanosoma cruzi: Mixture of two populations can modify virulence and tissue tropism in rat. Exp. Parasitol..

[217] Andrade L.O., Machado C.R., Chiari E., Pena S.D., Macedo A.M. (1999). Differential tissue distribution of diverse clones of Trypanosoma cruzi in infected mice. Mol. Biochem. Parasitol..

[218] Lewis M.D., Fortes Francisco A., Taylor M.C., Burrell-Saward H., McLatchie A.P., Miles M.A., Kelly J.M. (2014). Bioluminescence imaging of chronic Trypanosoma cruzi infections reveals tissue-specific parasite dynamics and heart disease in the absence of locally persistent infection. Cell. Microbiol..

[219] McCall L.-I., Tripathi A., Vargas F., Knight R., Dorrestein P.C., Siqueira-Neto J.L. (2018). Experimental Chagas disease-induced perturbations of the fecal microbiome and metabolome. PLoS Negl. Trop. Dis..

[220] Urdaneta-Morales S., Nironi I. (1996). Trypanosoma cruzi in the anal glands of urban opossums. I—Isolation and experimental infections. Mem. Inst. Oswaldo Cruz.

[221] Forkner C.E., Zia L.S. (1934). Viable leishmania donovani in nasal and oral secretions of patients with kala-azar and the bearing of this finding on the transmission of the disease. J. Exp. Med..

[222] de Brito M.E.F., Almeida E.L., Medeiros A.C.R., Werkhäuser R.P., de Alexandre J.L.A., Sá B.S.L.F., Rodrigues E.H.G., Brandão-Filho S.P. (2018). Leishmania (Viannia) braziliensis isolated from the saliva of patients in a cutaneous leishmaniasis-endemic area of northeastern Brazil. Mem. Inst. Oswaldo Cruz.

[223] Siriyasatien P., Chusri S., Kraivichian K., Jariyapan N., Hortiwakul T., Silpapojakul K., Pym A.M., Phumee A. (2016). Early detection of novel Leishmania species DNA in the saliva of two HIV-infected patients. BMC Infect. Dis..

[224] Chusri S., Hortiwakul T., Silpapojakul K., Siriyasatien P. (2012). Consecutive cutaneous and visceral leishmaniasis manifestations involving a novel Leishmania species in two HIV patients in Thailand. Am. J. Trop. Med. Hyg..

[225] Pandey N., Siripattanapipong S., Leelayoova S., Manomat J., Mungthin M., Tan-ariya P., Bualert L., Naaglor T., Siriyasatien P., Phumee A. (2018). Detection of Leishmania DNA in saliva among patients with HIV/AIDS in Trang Province, southern Thailand. Acta Trop..

[226] Sriworarat C., Phumee A., Mungthin M., Leelayoova S., Siriyasatien P. (2015). Development of loop-mediated isothermal amplification (LAMP) for simple detection of Leishmania infection. Parasit. Vectors.

[227] Vaish M., Mehrotra S., Chakravarty J., Sundar S. (2011). Noninvasive molecular diagnosis of human visceral leishmaniasis. J. Clin. Microbiol..

[228] Das S., Halder A., Rabidas V.N., Mandal A., Das P. (2014). Specific noninvasive detection of Leishmania donovani in desquamated buccal cell swab samples from human visceral Leishmaniasis-HIV coinfected patients. J. Clin. Microbiol..

[229] Lombardo G., Pennisi M.G., Lupo T., Migliazzo A., Caprì A., Solano-Gallego L. (2012). Detection of Leishmania infantum DNA by real-time PCR in canine oral and conjunctival swabs and comparison with other diagnostic techniques. Vet. Parasitol..

[230] de Ferreira S.A., Almeida G.G., de Silva S.O., Vogas G.P., Fujiwara R.T., de Andrade A.S.R., Melo M.N. (2013). Nasal, oral and ear swabs for canine visceral leishmaniasis diagnosis: New practical approaches for detection of Leishmania infantum DNA. PLoS Negl. Trop. Dis..

[231] Masum M.A., Evans D.A. (1994). Agglutinating anti-leishmanial antibodies in the saliva of kala-azar patients. Trans. R. Soc. Trop. Med. Hyg..

[232] Cantos-Barreda A., Escribano D., Bernal L.J., Cerón J.J., Martínez-Subiela S. (2017). Quantification of anti-Leishmania antibodies in saliva of dogs. Vet. Parasitol..

[233] Rosenthal E. (1991). Leishmania in Bronchoalveolar Lavage. Ann. Intern. Med..

[234] Jokipii L., Salmela K., Saha H., Kyrönseppä H., Eklund B., Evans D., Von Willebrand E., Jokipii A.M.M. (1992). Leishmaniasis Diagnosed from Bronchoalveolar Lavage. Scand. J. Infect. Dis..

[235] Lejon V., Kwete J., Büscher P. (2003). Towards saliva-based screening for sleeping sickness?. Trop. Med. Int. Health.

[236] Lejon V., Jamonneau V., Solano P., Atchade P., Mumba D., Nkoy N., Bébronne N., Kibonja T., Balharbi F., Wierckx A. (2006). Detection of trypanosome-specific antibodies in saliva, towards non-invasive serological diagnosis of sleeping sickness. Trop. Med. Int. Health.

[237] Marsden P.D., Hagstrom J.W. (1968). Experimental Trypanosoma cruzi infection in beagle puppies. The effect of variations in the dose and source of infecting trypanosomes and the route of inoculation on the course of the infection. Trans. R. Soc. Trop. Med. Hyg..

[238] Pinho R.T., Pedrosa R.C., Costa-Martins P., Castello-Branco L.R. (1999). Saliva ELISA: A method for the diagnosis of chronic Chagas disease in endemic areas. Acta Trop..

[239] Cortes-Serra N., Pinazo M.-J., de la Torre L., Galizzi M., Gascon J., Bustamante J.M. (2018). Diagnosis of Trypanosoma cruzi Infection Status using Saliva of Infected Subjects. Am. J. Trop. Med. Hyg..

[240] Lourenço J.L.M., Minuzzi-Souza T.T.C., Silva L.R., Oliveira A.C., Mendonça V.J., Nitz N., Aguiar L.M.S., Gurgel-Gonçalves R. (2018). High frequency of trypanosomatids in gallery forest bats of a Neotropical savanna. Acta Trop..

[241] el Hassan A.M., Khalil E.A., el Sheikh E.A., Zijlstra E.E., Osman A., Ibrahim M.E. (1998). Post kala-azar ocular leishmaniasis. Trans. R. Soc. Trop. Med. Hyg..

[242] Ferrari T.C., Guedes A.C., Oréfice F., Genaro O., Pinheiro S.R., Marra M.A., Silveira I.L., Miranda M.O. (1990). Isolation of Leishmania sp. from aqueous humor of a patient with cutaneous disseminated leishmaniasis and bilateral iridocyclitis (preliminary report). Rev. Inst. Med. Trop. Sao Paulo.

[243] Reinecke P., Gabbart H.E., Strunk W., Lösche C.C. (2001). Ocular scleromalacia caused by leishmaniasis: A rare cause of scleral perforation. Br. J. Ophthalmol..

[244] Doroodgar M., Doroodgar M., Doroodgar A. (2017). Unusual Presentation of Cutaneous Leishmaniasis: Ocular Leishmaniasis. Case Rep. Infect. Dis..

[245] Abrishami M., Soheilian M., Farahi A., Dowlati Y. (2002). Successful treatment of ocular leishmaniasis. Eur. J. Dermatol..

[246] Nikandish M., Goyonlo V.M., Taheri A.R., Kiafar B. (2016). Ocular Leishmaniasis Treated by Intralesional Amphotericin B. Middle East Afr. J. Ophthalmol..

[247] Mencía-Gutiérrez E., Gutiérrez-Díaz E., Rodríguez-Peralto J.L., Monsalve-Córdova J. (2005). Old World eyelid cutaneous leishmaniasis: A case report. Dermatol. Online J..

[248] Mohammadpour I., Motazedian M.H., Handjani F., Hatam G.R. (2016). Cutaneous Leishmaniasis of the Eyelids: A Case Series with Molecular Identification and Literature Review. Korean J. Parasitol..

[249] Doroodgar M., Doroodgar M., Doroodgar A. (2017). Eyelid Cutaneous Leishmaniasis: A Case Report. Iran. J. Public Health.

[250] Razeghinejad M.R., Monabati A., Kadivar M.R., Alborzi A. (2017). Conjunctival leishmaniasis in a case of disseminated cutaneous leishmaniasis. Trop. Dr..

[251] Perrin-Terrin A., Auriol S., Mahieu L., Debard A., Eden A., Cassagne M., Pagot-Mathis V., Malecaze F., Soler V. (2014). Recurrent bilateral anterior uveitis due to Leishmania infantum in a patient with immune deficiency related to HIV infection: A case report and literature review. J. Fr. Ophtalmol..

[252] Nandy A., Addy M., Chowdhury A.B. (1991). Leishmanial blepharo-conjunctivitis. Trop. Geogr. Med..

[253] Di Pietro S., Bosco V.R.F., Crinò C., Francaviglia F., Giudice E. (2016). Prevalence, type, and prognosis of ocular lesions in shelter and owned-client dogs naturally infected by Leishmania infantum. Vet. World.

[254] McConnell E.E., Chaffee E.F., Cashell I.G., Garner F.M. (1970). Visceral leishmaniasis with ocular involvement in a dog. J. Am. Vet. Med. Assoc..

[255] Richter M., Schaarschmidt-Kiener D., Krudewig C. (2014). Ocular signs, diagnosis and long-term treatment with allopurinol in a cat with leishmaniasis. Schweiz. Arch. Tierheilkd..

[256] Veraldi S., Bottini S., Currò N., Gianotti R. (2010). Leishmaniasis of the eyelid mimicking an infundibular cyst and review of the literature on ocular leishmaniasis. Int. J. Infect. Dis..

[257] Garcia-Alonso M., Nieto C.G., Blanco A., Requena J.M., Alonso C., Navarrete I. (1996). Presence of antibodies in the aqueous humour and cerebrospinal fluid during Leishmania infections in dogs. Pathological features at the central nervous system. Parasite Immunol..

[258] García-Alonso M., Blanco A., Reina D., Serrano F.J., Alonso C., Nieto C.G. (1996). Immunopathology of the uveitis in canine leishmaniasis. Parasite Immunol..

[259] Naranjo C., Fondevila D., Leiva M., Roura X., Peña T. (2010). Detection of Leishmania spp. and associated inflammation in ocular-associated smooth and striated muscles in dogs with patent leishmaniosis. Vet. Ophthalmol..

[260] Naranjo C., Fondevila D., Leiva M., Roura X., Peña T. (2005). Characterization of lacrimal gland lesions and possible pathogenic mechanisms of keratoconjunctivitis sicca in dogs with leishmaniosis. Vet. Parasitol..

[261] Barbosa V.T., Silva M.A.G., Sousa M.G., Gering A.P., Santos H.D., Laus J.L. (2012). Detecção de formas amastigotas em exame parasitológico de esfregaço obtido a partir de suabe conjuntival de cães com leishmaniose visceral. Arq. Bras. Med. Vet. Zootec..

[262] Bielory B.P., Lari H.B., Mirani N., Kapila R., Fitzhugh V.A., Turbin R.E. (2011). Conjunctival squamous cell carcinoma harboring Leishmania amastigotes in a human immunodeficiency virus-positive patient. Arch. Ophthalmol..

[263] Naranjo C., Fondevila D., Altet L., Francino O., Ríos J., Roura X., Peña T. (2012). Evaluation of the presence of Leishmania spp. by real-time PCR in the lacrimal glands of dogs with leishmaniosis. Vet. J..

[264] Strauss-Ayali D., Jaffe C.L., Burshtain O., Gonen L., Baneth G., Strauss-Ayali D., Jaffe C.L., Burshtain O., Gonen L., Baneth G. (2004). Polymerase chain reaction using noninvasively obtained samples, for the detection of Leishmania infantum DNA in dogs. J. Infect. Dis..

[265] de Almeida Ferreira S., Ituassu L.T., de Melo M.N., de Andrade A.S.R. (2008). Evaluation of the conjunctival swab for canine visceral leishmaniasis diagnosis by PCR-hybridization in Minas Gerais State, Brazil. Vet. Parasitol..

[266] Pereira V.F., Benassi J.C., Starke-Buzetti W.A., Silva D.T., Ferreira H.L., Keid L.B., Soares R.M., de Azevedo Ruiz V.L., de Sousa Oliveira T.M.F. (2016). Detection of canine visceral leishmaniasis by conjunctival swab PCR. Rev. Soc. Bras. Med. Trop..

[267] Otranto D., Napoli E., Latrofa M.S., Annoscia G., Tarallo V.D., Greco G., Lorusso E., Gulotta L., Falsone L., Basano F.S. (2017). Feline and canine leishmaniosis and other vector-borne diseases in the Aeolian Islands: Pathogen and vector circulation in a confined environment. Vet. Parasitol..

[268] Leite R.S., de Almeida Ferreira S., Ituassu L.T., de Melo M.N., de Andrade A.S.R. (2010). PCR diagnosis of visceral leishmaniasis in asymptomatic dogs using conjunctival swab samples. Vet. Parasitol..

[269] Benassi J.C., Benvenga G.U., Ferreira H.L., Pereira V.F., Keid L.B., Soares R., de Sousa Oliveira T.M.F. (2017). Detection of Leishmania infantum DNA in conjunctival swabs of cats by quantitative real-time PCR. Exp. Parasitol..

[270] de Sousa Oliveira T.M.F., Pereira V.F., Benvenga G.U., Martin M.F.A., Benassi J.C., da Silva D.T., Starke-Buzetti W.A. (2015). Conjunctival swab PCR to detect Leishmania spp. in cats. Rev. Bras. Parasitol. Vet..

[271] Karakuş M., Arserim S.K., Erişöz Kasap Ö., Pekağırbaş M., Aküzüm D., Alten B., Töz S., Özbel Y. (2019). Vector and reservoir surveillance study in a canine and human leishmaniasis endemic area in most western part of Turkey, Karaburun. Acta Trop..

[272] Ionică A.M., Deak G., Kalmár Z., Gherman C.M., Mihalca A.D., Dumitrache M.O. (2017). Molecular Survey on Leishmania Infantum Infection in Red Foxes (Vulpes Vulpes) From Romania. Bull. Univ. Agric. Sci. Vet. Med. ClujNapoca. Vet. Med..

[273] Nsiangani L.N., Kaimbo Wa Kaimbo D., Kazumba M.L. (2016). Anterior uveitis as the first sign of human African trypanosomiasis: A case report. Med. Sante Trop..

[274] Morrison W.I., Murray M., Sayer P.D., Preston J.M. (1981). The pathogenesis of experimentally induced Trypanosoma brucei infection in the dog. I. Tissue and organ damage. Am. J. Pathol..

[275] Panigrahi P.N., Mahendran K., Jena S.C., Behera P., Mahajan S., Arjun K., Dey S. (2015). Trypanosoma evansi infection in a German shepherd dog—Apparent successful treatment using serial low dose of diminazene aceturate. Vet. Parasitol. Reg. Stud. Rep..

[276] Mortelmans J., Neetens A. (1975). Ocular lesions in experimental Trypanosoma brucei infection in cats. Acta Zool. Pathol. Antverp..

[277] Da Silva A.S., Pierezan F., Wolkmer P., Costa M.M., Oliveiro C.B., Tonin A.A., Santurio J.M., Lopes S.T.A., Monteiro S.G. (2010). Pathological Findings Associated with Experimental Infection by Trypanosoma evansi in Cats. J. Comp. Pathol..

[278] Morales I., de León M., Morales M., Dalla F., Gutierrez C. (2006). Ocular lesions associated with Trypanosoma evansi in experimentally infected goats. Vet. Parasitol..

[279] Prata A. (1999). Evolution of the clinical and epidemiological knowledge about Chagas disease 90 years after its discovery. Mem. Inst. Oswaldo Cruz.

[280] Marsden P.D. (1967). Trypanosoma cruzi infections in CFI mice. II. Infections induced by different routes. Ann. Trop. Med. Parasitol..

[281] Giddings O.K., Eickhoff C.S., Smith T.J., Bryant L.A., Hoft D.F. (2006). Anatomical route of invasion and protective mucosal immunity in Trypanosoma cruzi conjunctival infection. Infect. Immun..

[282] Bahia M.T., Tafuri W.L., Caliari M.V., Veloso V.M., Carneiro C.M., Coelho G.L.L.M., Lana M. (2002). Comparison of Trypanosoma cruzi infection in dogs inoculated with blood or metacyclic trypomastigotes of Berenice-62 and Berenice-78 strains via intraperitoneal and conjunctival routes. Rev. Soc. Bras. Med. Trop..

[283] Conrady C.D., Hanson K.E., Mehra S., Carey A., Larochelle M., Shakoor A. (2018). The First Case of Trypanosoma cruzi-Associated Retinitis in an Immunocompromised Host Diagnosed With Pan-Organism Polymerase Chain Reaction. Clin. Infect. Dis..

[284] Herrera L., Martínez C., Carrasco H., Jansen A.M., Urdaneta-Morales S. (2007). Cornea as a tissue reservoir of Trypanosoma cruzi. Parasitol. Res..

[285] Shiadeh M.N., Niyyati M., Fallahi S., Rostami A. (2016). Human parasitic protozoan infection to infertility: A systematic review. Parasitol. Res..

[286] Teichmann C., Da Silva A., Monteiro S., Barbosa C., Barcelos R. (2011). Evidence of Venereal and Transplacental Transmission of Canine Visceral Leishmaniasis in Southern Brazil. ACTA Sci. Vet..

[287] Naucke T., Lorentz S. (2013). Non-sandfly transmission of canine leishmaniasis. Tieraerztl. Umsch..

[288] Karkamo V., Kaistinen A., Näreaho A., Dillard K., Vainio-Siukola K., Vidgrén G., Tuoresmäki N., Anttila M. (2014). The first report of autochthonous non-vector-borne transmission of canine leishmaniosis in the Nordic countries. Acta Vet. Scand..

[289] Schubach A., Cuzzi-Maya T., Gonçalves-Costa S.C., Pirmez C., Oliveira-Neto M.P. (1998). Leishmaniasis of glans penis. J. Eur. Acad. Dermatol. Venereol..

[290] Cabello I., Caraballo A., Millan Y. (2002). Leishmaniasis in the genital area. Rev. Inst. Med. Trop. Sao Paulo.

[291] Cain C., Stone M.S., Thieberg M., Wilson M.E. (1994). Nonhealing genital ulcers. Cutaneous leishmaniasis. Arch. Dermatol..

[292] Andrade Z.A., Andrade S.G. (1966). Some new aspects of the kala-azar pathology. (Morphologic study of 13 autopsy cases). Rev. Inst. Med. Trop. Sao Paulo.

[293] Martínez-García F., Regadera J., Mayer R., Sanchez S., Nistal M. (1996). Protozoan infections in the male genital tract. J. Urol..

[294] Diniz S.A., Melo M.S., Borges A.M., Bueno R., Reis B.P., Tafuri W.L., Nascimento E.F., Santos R.L. (2005). Genital lesions associated with visceral leishmaniasis and shedding of Leishmania sp. in the semen of naturally infected dogs. Vet. Pathol..

[295] Mir F., Fontaine E., Reyes-Gomez E., Carlus M., Fontbonne A. (2012). Subclinical leishmaniasis associated with infertility and chronic prostatitis in a dog. J. Small Anim. Pract..

[296] Boechat V.C., Mendes Junior A.A.V., de Madeira M.F., Ferreira L.C., Figueiredo F.B., Rodrigues F., Oliveira V., de Oliveira R.V.C., Menezes R.C. (2016). Occurrence of Leishmania infantum and associated histological alterations in the genital tract and mammary glands of naturally infected dogs. Parasitol. Res..

[297] Silva L.C., Assis V.P., Ribeiro V.M., Tafuri W.L., Toledo Júnior J.C., Silva S.O., Melo M.N., Rachid M.A., Valle G.R. (2014). Detection of Leishmania infantum in the smegma of infected dogs. Arq. Bras. Med. Vet. Zootec..

[298] Hernández L., Montoya A., Checa R., Dado D., Gálvez R., Otranto D., Latrofa M.S., Baneth G., Miró G. (2015). Course of experimental infection of canine leishmaniosis: Follow-up and utility of noninvasive diagnostic techniques. Vet. Parasitol..

[299] Silva F.L., Rodrigues A.A.M., Rego I.O.P., Santos R.L.R.L.H., Oliveira R.G., Silva T.M.A., Xavier M.N., Nascimento E.F., Santos R.L.R.L.H. (2008). Genital lesions and distribution of amastigotes in bitches naturally infected with Leishmania chagasi. Vet. Parasitol..

[300] Bouteille B., Buguet A. (2012). The detection and treatment of human African trypanosomiasis. Res. Rep. Trop. Med..

[301] Apted F., Mulligan H., Pott W. (1970). Clinical manifestations and diagnosis of sleep-ing sickness. The African Trypanosomiases.

[302] Rocha G., Martins A., Gama G., Brandão F., Atouguia J. (2004). Possible cases of sexual and congenital transmission of sleeping sickness. Lancet.

[303] Suganuma K., Narantsatsral S., Battur B., Yamasaki S., Otgonsuren D., Musinguzi S.P., Davaasuren B., Battsetseg B., Inoue N. (2016). Isolation, cultivation and molecular characterization of a new Trypanosoma equiperdum strain in Mongolia. Parasit. Vectors.

[304] Danek J. (2005). Microorganisms in stallion semen. Med. Weter. Vet. Med. Sci. Pract..

[305] Ahmed Y., Hagos A., Merga B., Van Soom A., Duchateau L., Goddeeris B., Govaere J. (2018). Trypanosoma equiperdum in the horse - a neglected threat?. Vlaams Diergeneeskd. Tijdschr..

[306] Al-Busadah K.A., El-Bahr S.M., Khalafalla A.I. (2017). Serum biochemical profile and molecular detection of pathogens in semen of infertile male dromedary camels (Camelus dromedarius). Anim. Reprod. Sci..

[307] Ogundele F.A., Okubanjo O.O., Ajanusi O.J., Fadason S.T. (2016). Semen characteristics and reaction time of Yankasa rams experimentally infected with Trypanosoma evansi infection. Theriogenology.

[308] Bezerra N.M., Moura G.H.F., de Araújo H.N., Bezerra F.S.B., de Paiva K.A.R., de Freitas Mendonça Costa K.M., Costa W.P., Medeiros D.A.S., Batista J.S. (2018). Detection of Trypanosoma vivax DNA in semen from experimentally infected goats. Vet. Res. Commun..

[309] Ikede B.O. (1979). Genital lesions in experimental chronic Trypanosoma brucei infection in rams. Res. Vet. Sci..

[310] Anosa V.O., Isoun T.T. (1980). Further observations on the testicular pathology in Trypanosoma vivax infection of sheep and goats. Res. Vet. Sci..

[311] Kaaya G.P., Oduor-Okelo D. (1980). The effects of Trypanosoma congolense infection on the testis and epididymis of the goat. Bull. Anim. Health Prod. Afr..

[312] Isoun T.T., Anosa V.O. (1974). Lesions in the reproductive organs of sheep and goats experimentally infected with Trypanosoma vivax. Tropenmed. Parasitol..

[313] Adamu S., Fatihu M.Y., Useh N.M., Mamman M., Sekoni V.O., Esievo K.A.N. (2007). Sequential testicular and epididymal damage in Zebu bulls experimentally infected with Trypanosoma vivax. Vet. Parasitol..

[314] Anosa V.O., Isoun T.T. (1983). Pathology of experimental Trypanosoma vivax infection in sheep and goats. Zentralbl. Veterinarmed. B.

[315] Claes F., Vodnala S.K., van Reet N., Boucher N., Lunden-Miguel H., Baltz T., Goddeeris B.M., Büscher P., Rottenberg M.E. (2009). Bioluminescent imaging of Trypanosoma brucei shows preferential testis dissemination which may hamper drug efficacy in sleeping sickness. PLoS Negl. Trop. Dis..

[316] Carvalho T., Trindade S., Pimenta S., Santos A.B., Rijo-Ferreira F., Figueiredo L.M. (2018). Trypanosoma brucei triggers a marked immune response in male reproductive organs. PLoS Negl. Trop. Dis..

[317] Araujo P.F., Almeida A.B., Pimentel C.F., Silva A.R., Sousa A., Valente S.A., Valente V.C., Britto M.M., Rosa A.C., Alves R.M. (2017). Sexual transmission of American trypanosomiasis in humans: A new potential pandemic route for Chagas parasites. Mem. Inst. Oswaldo Cruz.

[318] Bittencourt A.L. (1963). Chagasic placentitis and congenital transmission of Chagas’ disease. Rev. Inst. Med. Trop. Sao Paulo.

[319] Vianna G. (1911). Contribuicao para o estudo da anatomia patolojica da “molestia de Carlos Chagas”. (Esquizotripanoze humana ou tireoidite parazitaria). Mem. Inst. Oswaldo Cruz.

[320] Chagas C. (1916). Tripanosomiase americana. Forma aguda da mo- 16stia. Mem. Inst. Oswaldo Cruz.

[321] Lamano Carvalho T., Ferreira A., Sahao M. (1982). Alteracoes do testIculo humano na molestia de Chagas. II—Estudo morfometrico do tecido intersticial. Rev. Inst. Med. Trop. Sao Paulo.

[322] Lenzi H.L., Castelo-Branco M.T., Pelajo-Machado M., Oliveira D.N., Gattass C.R. (1998). Trypanosoma cruzi: Compromise of reproductive system in acute murine infection. Acta Trop..

[323] Carvalho L.O.P., Abreu-Silva A.L., de Hardoim D.J., Tedesco R.C., Mendes V.G., da Costa S.C.G., da Calabrese K.S. (2009). Trypanosoma cruzi and myoid cells from seminiferous tubules: Interaction and relation with fibrous components of extracellular matrix in experimental Chagas’ disease. Int. J. Exp. Pathol..

[324] Ribeiro M., Nitz N., Santana C., Moraes A., Hagström L., Andrade R., Rios A., Sousa A., Dallago B., Gurgel-Gonçalves R. (2016). Sexual transmission of Trypanosoma cruzi in murine model. Exp. Parasitol..

[325] Crespillo-Andujar C., Díaz-Menéndez M., Mora-Rillo M. (2018). Evidence for Previously Unidentified Sexual Transmission of Protozoan Parasites. Emerg. Infect. Dis..

[326] Anderson C. (1935). Nouveaux essais de culture de Leishmania donovani. Arch. Inst. Pasteur Tunis..

[327] Henry A., Guilhon J. (1944). LE ROLE DU LAIT DANS LA TRANSMISSION DE QUELQUES PROTOZOOSES. Le Lait.

[328] Nathan-Larrier L. (1913). Sur le passage des trypanosomes dans le lait. Rev. Pathol. Comp..

[329] Calvo-Méndez M.L., Nogueda-Torres B., Alejandre-Aguilar R., Cortés-Jiménez M. (1994). Experimental Trypanosoma cruzi infection via contaminated water and food. Rev. Latinoam. Microbiol..

[330] Disko R., Krampitz H.E. (1971). Occurrence of Trypanosoma cruzi in the milk of infected mice. Z. Tropenmed. Parasitol..

[331] Miles M.A. (1972). Trypanosoma cruzi—Milk transmission of infection and immunity from mother to young. Parasitology.

[332] das Medina-Lopes M.D., Macêdo V. (1983). Trypanosoma cruzi no colostro humano. Rev. Soc. Bras. Med. Trop..

[333] Jörg M.E. (1992). The transmission of Trypanosoma cruzi via human milk. Rev. Soc. Bras. Med. Trop..

[334] Amato Neto V., Matsubara L., Campos R., Moreira A.A., Pinto P.L., Faccioli R., Zugaib M. (1992). Trypanosoma cruzi in the milk of women with chronic Chagas disease. Rev. Hosp. Clin..

[335] Campos R., Pinto P.L., Moreira A.A., Amato Neto V., Duarte M.I., de Sant’Ana E.J., Tiago G.G. (1988). Experimental study on the transmission of Chagas’ disease by milk. Rev. Hosp. Clin..

[336] Norman F.F., López-Vélez R. (2013). Chagas disease and breast-feeding. Emerg. Infect. Dis..

[337] Ribeiro R.D., Lopes R.A., Garcia T.A., Campos A. (1988). Histopathological study of the mammary gland in Trypanosoma cruzi-infected mice. Parasitol. Res..

[338] Ferreira C.S., Martinho P.C., Amato Neto V., Cruz R.R. (2001). Pasteurization of human milk to prevent transmission of Chagas disease. Rev. Inst. Med. Trop. Sao Paulo.

[339] Santos Ferreira C., Amato Neto V., Gakiya E., Bezerra R.C., Alarcón R.S.R. (2003). Microwave treatment of human milk to prevent transmission of Chagas disease. Rev. Inst. Med. Trop. Sao Paulo.

[340] Canário A., Queiroz M., Cunha G., Cavalcante T., Riesz V., Sharma R., de Noronha A., Correia T., Barral-Netto M., Barral A. (2019). Presence of parasite DNA in clinically unaffected nasal mucosa during cutaneous leishmaniasis caused by Leishmania (Viannia) braziliensis. Clin. Microbiol. Infect..

[341] Harrison N., Walochnik J., Ramsebner R., Veletzky L., Lagler H., Ramharter M. (2017). Progressive Perforation of the Nasal Septum Due to Leishmania major: A Case of Mucosal Leishmaniasis in a Traveler. Am. J. Trop. Med. Hyg..

[342] Muñoz-Madrid R., Belinchón-Lorenzo S., Iniesta V., Fernández-Cotrina J., Parejo J.C., Serrano F.J., Monroy I., Baz V., Gómez-Luque A., Gómez-Nieto L.C. (2013). First detection of Leishmania infantum kinetoplast DNA in hair of wild mammals: Application of qPCR method to determine potential parasite reservoirs. Acta Trop..

[343] Belinchón-Lorenzo S., Muñoz-Madrid R., Grano F.G., Iniesta V., Fernández-Cotrina J., Parejo J.C., Monroy I., Baz V., Gómez-Luque A., Barneto J.L. (2019). Application of qPCR method to hair and cerumen samples for the diagnosis of canine leishmaniosis in Araçatuba, Brazil. Vet. Parasitol. Reg. Stud. Rep..

[344] Ferro C., Cadena H., Travi B.L., Tabares C.J., Osorio Y. (2001). Canine visceral leishmaniasis in Colombia: Relationship between clinical and parasitologic status and infectivity for sand flies. Am. J. Trop. Med. Hyg..

[345] Goodrich E.S., Sears S.C., Sorrells T., Radike J.K., Miladi A., Glass J.S. (2017). A Case of Cutaneous Leishmaniasis guyanensis Mimicking Otitis Externa. Mil. Med..

[346] Penn D., Potts W.K. (1998). Chemical signals and parasite-mediated sexual selection. Trends Ecol. Evol..

[347] de Oliveira L.S., de Rodrigues F.M., de Oliveira F.S., Mesquita P.R.R., Leal D.C., Alcântara A.C., Souza B.M., Franke C.R., de Pereira P.A.P., de Andrade J.B. (2008). Headspace solid phase microextraction/gas chromatography-mass spectrometry combined to chemometric analysis for volatile organic compounds determination in canine hair: A new tool to detect dog contamination by visceral leishmaniasis. J. Chromatogr. B Analyt. Technol. Biomed. Life Sci..

[348] Magalhães-Junior J.T., Mesquita P.R.R., Oliveira W.F., Oliveira F.S., Franke C.R., de Rodrigues F.M., de Andrade J.B., Barrouin-Melo S.M. (2014). Identification of biomarkers in the hair of dogs: New diagnostic possibilities in the study and control of visceral leishmaniasis. Anal. Bioanal. Chem..

[349] Iniesta V., Belinchón-Lorenzo S., Soto M., Fernández-Cotrina J., Muñoz-Madrid R., Monroy I., Baz V., Gómez-Luque A., Parejo J.C., Alonso C. (2013). Detection and chronology of parasitic kinetoplast DNA presence in hair of experimental Leishmania major infected BALB/c mice by Real Time PCR. Acta Trop..

[350] Ortega M.V., Moreno I., Domínguez M., de la Cruz M.L., Martín A.B., Rodríguez-Bertos A., López R., Navarro A., González S., Mazariegos M. (2017). Application of a specific quantitative real-time PCR (qPCR) to identify Leishmania infantum DNA in spleen, skin and hair samples of wild Leporidae. Vet. Parasitol..

[351] de Sousa Gonçalves R., Franke C.R., Magalhães-Junior J.T., Souza B.M.P.S., Solcà M.S., Larangeira D.F., Barrouin-Melo S.M. (2016). Association between Leishmania infantum DNA in the hair of dogs and their infectiousness to Lutzomyia longipalpis. Vet. Parasitol..

[352] Karram S., Loya A., Hamam H., Habib R.H., Khalifeh I. (2012). Transepidermal elimination in cutaneous leishmaniasis: A multiregional study. J. Cutan. Pathol..

[353] Vasconcellos C., Sotto M.N. (1997). Experimental cutaneous leishmaniasis: Transmission electron microscopy of the inoculation site. Int. J. Exp. Pathol..

[354] de Mendonça I.L., Batista J.F., Alves L.C. (2015). Leishmania (infantum) chagasi in canine urinary sediment. Rev. Bras. Parasitol. Vet..

[355] Ghosh P., Bhaskar K.R.H., Hossain F., Khan M.A.A., Vallur A.C., Duthie M.S., Hamano S., Salam M.A., Huda M.M., Khan M.G.M. (2016). Evaluation of diagnostic performance of rK28 ELISA using urine for diagnosis of visceral leishmaniasis. Parasit. Vectors.

[356] Vogt F., Mengesha B., Asmamaw H., Mekonnen T., Fikre H., Takele Y., Adem E., Mohammed R., Ritmeijer K., Adriaensen W. (2018). Antigen Detection in Urine for Noninvasive Diagnosis and Treatment Monitoring of Visceral Leishmaniasis in Human Immunodeficiency Virus Coinfected Patients: An Exploratory Analysis from Ethiopia. Am. J. Trop. Med. Hyg..

[357] Fernández-Roldán C., Rodríguez-Grangér J., Javier Martínez R., López-Ruz M.A., Navarro-Marí J.M., Gutiérrez-Fernández J. (2017). Performance of the KAtex test in screening and diagnosis for visceral leishmaniasis in a reference hospital. Rev. Esp. Quimioter..

[358] Salam M.A., Khan M.G.M., Mondal D. (2011). Urine antigen detection by latex agglutination test for diagnosis and assessment of initial cure of visceral leishmaniasis. Trans. R. Soc. Trop. Med. Hyg..

[359] Corral R.S., Altcheh J.M., Freilij H.L. (1998). Presence of IgM antibodies to Trypanosoma cruzi urinary antigen in sera from patients with acute Chagas’ disease. Int. J. Parasitol..

[360] García-García J.A., Martín-Sánchez J., Gállego M., Rivero-Román A., Camacho A., Riera C., Morillas-Márquez F., Vergara S., Macías J., Pineda J.A. (2006). Use of noninvasive markers to detect Leishmania infection in asymptomatic human immunodeficiency virus-infected patients. J. Clin. Microbiol..

[361] Abeijon C., Dilo J., Tremblay J.M., Viana A.G., Bueno L.L., Carvalho S.F.G., Fujiwara R.T., Shoemaker C.B., Campos-Neto A. (2018). Use of VHH antibodies for the development of antigen detection test for visceral leishmaniasis. Parasite Immunol..

[362] Cruz I., Chicharro C., Nieto J., Bailo B., Cañavate C., Figueras M.-C., Alvar J. (2006). Comparison of new diagnostic tools for management of pediatric Mediterranean visceral leishmaniasis. J. Clin. Microbiol..

[363] Abeijon C., Campos-Neto A. (2013). Potential non-invasive urine-based antigen (protein) detection assay to diagnose active visceral leishmaniasis. PLoS Negl. Trop. Dis..

[364] Abeijon C., Singh O.P., Chakravarty J., Sundar S., Campos-Neto A. (2016). Novel Antigen Detection Assay to Monitor Therapeutic Efficacy of Visceral Leishmaniasis. Am. J. Trop. Med. Hyg..

[365] Boni S.M., Oyafuso L.K., de Soler R.C., Lindoso J.A.L. (2017). Efficiency of noninvasive sampling methods (swab) together with Polymerase Chain Reaction (PCR) for diagnosing American Tegumentary Leishmaniasis. Rev. Inst. Med. Trop. Sao Paulo.

[366] Aschar M., de Oliveira E.T.B., Laurenti M.D., Marcondes M., Tolezano J.E., Hiramoto R.M., Corbett C.E.P., da Matta V.L.R. (2016). Value of the oral swab for the molecular diagnosis of dogs in different stages of infection with Leishmania infantum. Vet. Parasitol..

[367] de Almeida Ferreira S., Leite R.S., Ituassu L.T., Almeida G.G., Souza D.M., Fujiwara R.T., de Andrade A.S.R., Melo M.N. (2012). Canine skin and conjunctival swab samples for the detection and quantification of Leishmania infantum DNA in an endemic urban area in Brazil. PLoS Negl. Trop. Dis..

[368] Di Muccio T., Veronesi F., Antognoni M.T., Onofri A., Piergili Fioretti D., Gramiccia M. (2012). Diagnostic value of conjunctival swab sampling associated with nested PCR for different categories of dogs naturally exposed to Leishmania infantum infection. J. Clin. Microbiol..

[369] Solano-Gallego L., Morell P., Arboix M., Alberola J., Ferrer L. (2001). Prevalence of Leishmania infantum infection in dogs living in an area of canine leishmaniasis endemicity using PCR on several tissues and serology. J. Clin. Microbiol..

[370] Ceccarelli M., Galluzzi L., Sisti D., Bianchi B., Magnani M. (2014). Application of qPCR in conjunctival swab samples for the evaluation of canine leishmaniasis in borderline cases or disease relapse and correlation with clinical parameters. Parasit. Vectors.

[371] Carvalho Ferreira A.L., Carregal V.M., de Almeida Ferreira S., Leite R.S., de Andrade A.S.R. (2014). Detection of Leishmania infantum in 4 different dog samples by real-time PCR and ITS-1 nested PCR. Diagn. Microbiol. Infect. Dis..

[372] Geisweid K., Weber K., Sauter-Louis C., Hartmann K. (2013). Evaluation of a conjunctival swab polymerase chain reaction for the detection of Leishmania infantum in dogs in a non-endemic area. Vet. J..

[373] Pilatti M.M., de Ferreira S.A., de Melo M.N., de Andrade A.S.R. (2009). Comparison of PCR methods for diagnosis of canine visceral leishmaniasis in conjunctival swab samples. Res. Vet. Sci..

[374] Gao C., Ding D., Wang J., Steverding D., Wang X., Yang Y., Shi F. (2015). Development of a LAMP assay for detection of Leishmania infantum infection in dogs using conjunctival swab samples. Parasit. Vectors.

[375] Belinchón-Lorenzo S., Parejo J.C., Iniesta V., Fernández-Cotrina J., Muñoz-Madrid R., Monroy I., Baz V., Gómez-Luque A., Serrano-Aguilera F.J., Barneto J.L. (2016). First detection of Leishmania kDNA in canine cerumen samples by qPCR. Vet. Parasitol..

[376] Belinchón-Lorenzo S., Iniesta V., Parejo J.C., Fernández-Cotrina J., Muñoz-Madrid R., Soto M., Alonso C., Gómez Nieto L.C. (2013). Detection of Leishmania infantum kinetoplast minicircle DNA by Real Time PCR in hair of dogs with leishmaniosis. Vet. Parasitol..

[377] Moher D., Liberati A., Tetzlaff J., Altman D.G. (2009). Preferred Reporting Items for Systematic Reviews and Meta-Analyses: The PRISMA Statement. PLoS Med..

[378] Nakagawa S., Noble D.W.A., Senior A.M., Lagisz M. (2017). Meta-evaluation of meta-analysis: Ten appraisal questions for biologists. BMC Biol..

[379] Freeman M., Tukey J. (1950). Transformations related to the angular and the square root. Ann. Math. Stat..

[380] Barendregt J.J., Doi S.A., Lee Y.Y., Norman R.E., Vos T. (2013). Meta-analysis of prevalence. J. Epidemiol. Community Health.

[381] Egger M., Davey Smith G., Schneider M., Minder C. (1997). Bias in meta-analysis detected by a simple, graphical test. BMJ.

