# Supplementary material for: Noninvasive Biological Samples to Detect and Diagnose Infections due to Trypanosomatidae Parasites: A Systematic Review and Meta-Analysis
Source: Int J Mol Sci. 2020 Feb 29;21(5):1684. doi: 10.3390/ijms21051684 (PMC7084391; doi:10.3390/ijms21051684)
Supplement: Supplementary file 1 [file ijms-21-01684-s001.zip › Supplementary data/TablerecapS3.docx]

TOverview of the number of data extracted according to the pathology, the origin of the biological sample, and the methodology for detection.

|  |  |  | Leish* | CD | HAT | AT |
| --- | --- | --- | --- | --- | --- | --- |
| Urine | Molecular | DNA/RNA | 9 | - | - | - |
|  | Immuno | Ab | 14 | - | - | - |
|  |  | Ag | 23 | 7 | - | - |
|  | Parasito | Parasite | 5 | - | - | - |
| Conjunctival swab | Molecular | DNA/RNA | 16 | - | - | - |
|  | Immuno | Ab | 1 | - | - | - |
|  |  | Ag | - | - | - | - |
|  | Parasito | Parasite | - | - | - | - |
| Oral/Saliva | Molecular | DNA/RNA | 6 | - | - | - |
|  | Immuno | Ab | - | 1 | 2 | - |
|  |  | Ag | 1 | - | - | - |
|  | Parasito | Parasite | - | - | - | - |
| Nasal | Molecular | DNA/RNA | 2 | - | - | - |
|  | Immuno | Ab | - | - | - | - |
|  |  | Ag | - | - | - | - |
|  | Parasito | Parasite | 1 | - | - | - |
| Hair/Bristle | Molecular | DNA/RNA | 4 | - | - | - |
|  | Immuno | Ab | - | - | - | - |
|  |  | Ag | - | - | - | - |
|  | Parasito | Parasite | - | - | - | - |

*Canine visceral leishmaniasis, visceral, cutaneous mucocutaneous Leishmaniasis
