# Supplementary material for: Noninvasive Biological Samples to Detect and Diagnose Infections due to Trypanosomatidae Parasites: A Systematic Review and Meta-Analysis
Source: Int J Mol Sci. 2020 Feb 29;21(5):1684. doi: 10.3390/ijms21051684 (PMC7084391; doi:10.3390/ijms21051684)
Supplement: Supplementary file 1 [file ijms-21-01684-s001.zip › Supplementary data/DatacollectionS2.docx]

**DatacollectionS2: Datasheet of quantitative data extracted**

1

| **References** | de Mendonça IL, Batista JF, Alves LC. Leishmania (infantum) chagasi in canine urinary sediment. Rev Bras Parasitol Vet. 2015 Jan-Mar;24(1):92-4. | | | |
| --- | --- | --- | --- | --- |
| **Authors** | de Mendonça IL, Batista JF, Alves LC. | | | |
| **Year** | 2015 | **Journal** | Revista Brasiliera de Parasitologia Veterinaria. | |
| **Notes** |  | | | |
| **Host** | Dogs | **Infection** | CVL |  |
| **Country** | Brazil | **Clinic** | Clinical sign+ Blood/ Bone Marrow aspirate. ELISA | |
| **Parasite** | *L. infantum* | **Biological origin** | | Urine |
| **Nb total of subjects** | | 70 | | |
| **Positive Ref N** | 17 | **Positive experiment N** | | 4 |
| **Methodologies** | Molecular | Immunology | Parasitology | Other |
| **Technical** |  |  | NNN |  |
|  |  |  | 4 |  |
|  |  | **Positivity** | 23% | |

2

| **References** | Fisa, R., Riera, C., Lopez-Chejade, P., Gallego, M., Falco, V., Ribera, E., Portus, M. 2008. *Leishmania infantum* DNA detection in Urine from patients with Visceral Leishmaniasis and after treatment control. Am. J. Trop. Med & Hyg. 78 (5) : 741-745. | | | |
| --- | --- | --- | --- | --- |
| **Authors** | Fisa, R., Riera, C ;, Lopez-Chejade, P., Gallego, M., Falco, V., Ribera, E., Portus, M. | | | |
| **Year** | 2008 | **Journal** | Am. J. Trop. Med & Hyg | |
| **Notes** |  | | | |
| **Host** | Human | **Infection** | VL-HIV |  |
| **Country** | Spain | **Clinic** | Clinical symptoms, Bone Marrow aspirate : culture and staining | |
| **Parasite** | *L. infantum* | **Biological origin** | | Urine |
| **Nb total of subjects** | | 28 of which 24 are VIH+ | | |
| **Positive Ref N** | 17 | **Positive experiment N** | | 15 |
| **Methodologies** | Molecular | Immunology | Parasitology | Other |
| **Result** | PCR | Katex |  |  |
|  | 15/17 | 15/17 |  |  |
|  |  | **Positivity** | Katex 88.0%  PCR 88.0% | |

3

| **References** | Solano-Gallego, L., Rodriguez-Cortes, A., Trotta, M., Zampieron C., Razia, L., Furlanello, T., Caldin, M., Roura, X., Alberola, J. 2007. Detection of Leishmania infantum DNA bry fret-based real-time PCR in urine from dogs with natural clinical leishmaniosis. Vet. Parasitol. 147 : 315-319. | | | |
| --- | --- | --- | --- | --- |
| **Authors** | Solano-Gallego, L., Rodriguez-Cortes, A., Trotta, M., Zampieron C., Razia, L., Furlanello, T., Caldin, M., Roura, X., Alberola, J. | | | |
| **Year** | 2007 | **Journal** | Vet. Parasitol | |
| **Notes** |  | | | |
| **Host** | Dogs | **Infection** | CVL | |
| **Country** | Spain & Italia | **Clinic** | Clinic, IFA, Bone Marrow aspirate (culture & staining), PCR, ELISA Clinical | |
| **Parasite** | *L. infantum* | **Biological origin** | | Urine |
| **Nb total of subjects** | | 53 | | |
| **Positive Ref N** | 43  (29 spain  14 Italia) | **Positive experiment N** | | 20 |
| **Methodologies** | Molecular | Immunology | Parasitology | Other |
| **Result** | qPCR : 20 |  |  |  |
|  |  | **Positivity** | 47% |  |

4

| **References** | Pessoa-e-Silva, R., Mendoça Trajano-Silva, L.A., Lopes da Silva, M.A., Gonçalved-de-Albuquerque, S.C., Correira de Goes, T., Silva de Morais., R.C., Lopes de Melo, F., Paiva-Cavalcanti, M. 2016. Evaluation of urine for Leishmania infantum DNA detection by real-time quantitative PCR. J. Microbiol. Meth. 131 : 34-41. | | | |
| --- | --- | --- | --- | --- |
| **Authors** | Pessoa-e-Silva, R., Mendoça Trajano-Silva, L.A., Lopes da Silva, M.A., Gonçalved-de-Albuquerque, S.C., Correira de Goes, T., Silva de Morais., R.C., Lopes de Melo, F., Paiva-Cavalcanti, M. | | | |
| **Year** | 2016 | **Journal** | J. Microbiol. Meth. | |
| **Notes** |  | | | |
| **Host** | Humans | **Infection** | VL & HIV-VL | |
| **Country** | Brazil | **Clinic** | Clinic + Bone Marrow aspirate smear + PCR Blood+ rK39 | |
| **Parasite** | *L. infantum* | **Biological origin** | | Urine |
| **Nb total of subjects** | |  | | |
| **Positive Ref N** | 18 | **Positive experiment N** | | 4 |
| **Methodologies** | Molecular | Immunology | Parasitology | Other |
| **Result** | 4/18 |  |  |  |
|  |  | **Positivity** | 22.2% |  |

5

| **References** | Ghosh, P., Bashkar, K.R.H., Hossain, F., Ashfaq Khan, Md A., Vallur, A.A., Duthie, M.S., Hamano, S., Abdus Salam, Md., Huda, MH., Musawwir-Khan, Pd.G., Coler, R.N., Reed, S.G., Mondal, D. 2016. Evaluation of diagnostic performance of rk28 ELISA using urine for diagnosis of visceral leishmaniasis. Parasites & Vectors. 9 : 383 | | | | |
| --- | --- | --- | --- | --- | --- |
| **Authors** | Ghosh, P., Bashkar, K.R.H., Hossain, F., Ashfaq Khan, Md A., Vallur, A.A., Duthie, M.S., Hamano, S., Abdus Salam, Md., Huda, MH., Musawwir-Khan, Pd.G., Coler, R.N., Reed, S.G., Mondal, D. | | | | |
| **Year** | 2016 | **Journal** | Parasites & Vectors | | |
| **Notes** |  | | | | |
| **Host** | Humans | **Infection** | VL | | |
| **Country** | Bangladesh | **Clinic** | Parasite in spleen or DAT blood on filter paper | | |
| **Parasite** | *L. donovani* | **Biological origin** | | | Urine |
| **Nb total of subjects** | | 168 | | | |
| **Positive Ref N** | 87 | **Positive experiment N** | | | 79 to 83 |
| **Methodologies** | Molecular | Immunology | | Parasitology | Other |
| **Result** |  | Antibody capture with rK39 : 83  rK28 : 82  rKRP42 : 79 | |  |  |
|  |  | **Positivity** | rK28 : 95.4 %  rK39 : 94.3 %  rKRP42 : 90.8 % | | |

6

| **References** | Franceshi, A., Merildi, V., Guidi, G., Mancianti, F. 2007. Occurrence of Leishmania DNA in urines of dogs Naturally infected with Leishmaniasis. Vet. Res. Com. 31 : 335-341. | | | |
| --- | --- | --- | --- | --- |
| **Authors** | Franceshi, A., Merildi, V., Guidi, G., Mancianti, F. | | | |
| **Year** | 2007 | **Journal** | Vet. Res. Com | |
| **Notes** |  | | | |
| **Host** | Dogs | **Infection** | CVL | |
| **Country** | NK | **Clinic** | Olygosymptomatic, IFAT, Lymph node smear | |
| **Parasite** | *L. infantum* | **Biological origin** | | Urine |
| **Nb total of subjects** | | 40 | | |
| **Positive Ref N** | 16 | **Positive experiment N** | | 10 |
| **Methodologies** | Molecular | Immunology | Parasitology | Other |
| **Technical** | PCR |  |  |  |
| **Result** | 10 |  |  |  |
|  |  | **Positivity** | 62.5% |  |

7

| **References** | Mebrahtu, Y.B., Hendricks, L.D., Oster, C.N., Lawyer, P.G., Perkins, P.V., Pamba, H., Koech, D., Roberts, C.R. 1993. *Leishmania donovani* parasites in the nasal secretion, tonsillopharyngeal mucosa, and urine centrifugates of visceral leishmaniasis patients in Kenya. Am. J. Trop. Med & Hyg. 48 : 520-525. | | | |
| --- | --- | --- | --- | --- |
| **Authors** | Mebrahtu, Y.B., Hendricks, L.D., Oster, C.N., Lawyer, P.G., Perkins, P.V., Pamba, H., Koech, D., Roberts, C.R. | | | |
| **Year** | 1993 | **Journal** | Am. J. Trop. Med & Hyg. | |
| **Notes** |  | | | |
| **Host** | Humans | **Infection** | VL | |
| **Country** | Kenya | **Clinic** | spleen aspirate, Culture Staining parasites | |
| **Parasite** |  | **Biological origin** | | Urine  Nasal  Throat |
| **Nb total of subjects** | | 64 | | |
| **Positive Ref N** | 38 to 64 | **Positive experiment N** | | 2 to 23 |
| **Methodologies** | Molecular | Immunology | Parasitology | Other |
| **Result** |  |  | Urine : 2/38  Nasal : 23/64  Throat :3/64 |  |
|  |  | **Positivity** | Urine : 5.3 %  Nasal : 36.0 %  Throat : 4.7 % |  |

8

| **References** | Zaragoza, C., Barrera, R., Centeno, F., Tapia, J.A., Duran, E ;, Gonzalez, M., Mane, M.C. 2003. SDS-PAGE and western blot of urinary proteins in dogs with leishmaniasis. Vet. Res. 34 : 137-151. | | | |
| --- | --- | --- | --- | --- |
| **Authors** | Zaragoza, C., Barrera, R., Centeno, F., Tapia, J.A., Duran, E ;, Gonzalez, M., Mane, M.C. | | | |
| **Year** | 2003 | **Journal** | Vet. Res | |
| **Notes** |  | | | |
| **Host** | Dog | **Infection** | CVL | |
| **Country** | Spain | **Clinic** | Clinical signs + ELISA+ Blood PCR | |
| **Parasite** | *L. infanfum* | **Biological origin** | | Urine |
| **Nb total of subjects** | | 42 | | |
| **Positive Ref N** | 22 | **Positive experiment N** | | 15 |
| **Methodologies** | Molecular | Immunology | Parasitology | Other |
| **Result** |  | WB : 15 |  |  |
|  |  | **Positivity** | 68.0% |  |

9

| **References** | Abeijon, C., Campos-Neto, A. 2013. Potential Non-invasive urine-based antigen (Protein) detection assay to diagnose active visceral leishmaniasis. Plos. Negl. Trop. Dis. 7 : e2161 | | | | |
| --- | --- | --- | --- | --- | --- |
| **Authors** | Abeijon, C., Campos-Neto, A. | | | | |
| **Year** | 2013. | **Journal** | Plos. Negl. Trop. Dis | | |
| **Notes** |  | | | | |
| **Host** | Human | **Infection** | VL | | |
| **Country** | Brazil | **Clinic** | Previously diagnose as VL | | |
| **Parasite** | *L. infantum* | **Biological origin** | | | Urine |
| **Nb total of subjects** | | 20 | | | |
| **Positive Ref N** | 20 | **Positive experiment N** | | |  |
| **Methodologies** | Molecular | Immunology | | Parasitology | Other |
| **Result** |  | Ag Capture (ELISA)  SOD : 12  Li-txn : 9  Li-ntf2 : 11  **SOD + txn+stf2 : 20/2**0 | |  |  |
|  |  | **Positivity** | | SOD : 60.0%  Li-txn : 45.0 %  Li-ntf2 : 55.0%  SOD + txn+stf2 : 100.0% | |

10

| **References** | Riera, C., Fisa, R., Lopez, P., Ribera, E., Carrio, J., Falco, V., Molina, I., Gallego, M., Portus, M. 2004. Evaluation of a latex agglutination test (Katex) for detection of Leishmania antigen in urine of patients HIV-Leishmania coninfection : value in diagnosis and post treatment follow-up. Eur. J. Clin. Microbiol. Infect. Dis. 23 : 899-904. | | | |
| --- | --- | --- | --- | --- |
| **Authors** | Riera, C ;, Fisa, R., Lopez, P., Ribera, E., Carrio, J., Falco, V., Molina, I., Gallego, M., Portus, M. | | | |
| **Year** | 2004 | **Journal** | Eur. J. Clin. Microbiol. Infect. Dis. | |
| **Notes** |  | | | |
| **Host** | Human | **Infection** | VL-HIV | |
| **Country** | Spain | **Clinic** | Clinical symptoms- Bone Marrow examination and parasite isolation. | |
| **Parasite** | *L. infantum* | **Biological origin** | | Urine |
| **Nb total of subjects** | | 109 (49 VL-HIV, 16 HIV, 43 Control) | | |
| **Positive Ref N** | 49 | **Positive experiment N** | | 42 |
| **Methodologies** | Molecular | Immunology | Parasitology | Other |
| **Result** |  | Antgigen agglutination test Katex : 42/49 |  |  |
|  |  | **Positivity** | 85.7% |  |

11

| **References** | Veland, N., Espinosa, D., Valencia, B.M., Ramos, A.P., Calderon, F., Arevalo, J., Low, D.R., Llanos-Cuentas, A., Boggild, A.K. 2011. Polymerase chain reaction detection of Leishmania kDNA from the urine of Peruvian patients with cutaneous and Mucocutaneous leishmaniasis. Am. J. Trop. Med & Hyg. 84 : 556-561. | | | |
| --- | --- | --- | --- | --- |
| **Authors** | Veland, N., Espinosa, D., Valencia, B.M., Ramos, A.P., Calderon, F., Arevalo, J., Low, D.R., Llanos-Cuentas, A., Boggild, A.K. | | | |
| **Year** | 2011. | **Journal** | Am. J. Trop. Med & Hyg | |
| **Notes** |  | | | |
| **Host** | Humans | **Infection** | CL, MCL | |
| **Country** | Peru | **Clinic** | Scrapping, smear, aspirate | |
| **Parasite** | *Leishmania (Viannia)*  *L. guyanensis*  *L. panamensis*  *L. braziliensis* | **Biological origin** | | Urine |
| **Nb total of subjects** | | 108 | | |
| **Positive Ref N** | 86 | **Positive experiment N** | | 18 |
| **Methodologies** | Molecular | Immunology | Parasitology | Other |
| **Result** | kDNA PCR  18/86 |  |  |  |
|  |  | **Positivity** | 20.9% |  |

12

| **References** | Abeijon, C., KAshino, S.S., Silva, F.O., Costa, D.L., Fujixara, R.T., Costa, C.H.N., Campos-Neto, A. 2012. Identification and diagnostic utility of Leishmania infantum proteins found in urine samples from patients with visceral leishmaniasis. Clin. Vaccine. Immunol. 19 : 935-943. | | | |
| --- | --- | --- | --- | --- |
| **Authors** | Abeijon, C., KAshino, S.S., Silva, F.O., Costa, D.L., Fujixara, R.T., Costa, C.H.N., Campos-Neto, A. | | | |
| **Year** | 2012 | **Journal** | Clin. Vaccine. Immunol. | |
| **Notes** |  | | | |
| **Host** | Humans | **Infection** | VL | |
| **Country** | Brazil | **Clinic** | Clinical symptoms, Bone Marrow aspirate and PCR | |
| **Parasite** | *L. infantum* | **Biological origin** | | Urine |
| **Nb total of subjects** | | 25 | | |
| **Positive Ref N** | 19 | **Positive experiment N** | | 17 |
| **Methodologies** | Molecular | Immunology | Parasitology | Other |
| **Result** |  | Ag Capture (ELISA)  SOD + txn+stf2 : 17/19 |  |  |
|  |  | **Positivity** | 89.5% | |

13

| **References** | Kohanteb, J., Ardehalli, S.M., Rezai, H.R. Detection of Leishmania donovani soluble antigen and antibody in the urine of visceral leishmaniasis pateints. 1987. Trans. Roy. Soc. Trop. Med & Hyg. 81 : 578-580. | | | |
| --- | --- | --- | --- | --- |
| **Authors** | Kohanteb, J., Ardehalli, S.M., Rezai, H.R. | | | |
| **Year** | 1987 | **Journal** | Trans. Roy. Soc. Trop. Med & Hyg | |
| **Notes** |  | | | |
| **Host** | Human | **Infection** | VL | |
| **Country** | Iran | **Clinic** | Clinical symptoms and serology | |
| **Parasite** | *L. infantum ?* | **Biological origin** | | Urine |
| **Nb total of subjects** | | 31 | | |
| **Positive Ref N** | 21 | **Positive experiment N** | | 21 |
| **Methodologies** | Molecular | Immunology | Parasitology | Other |
| **Result** |  | Ab diffusion method : 21/21  Ag Capture : 19/21 |  |  |
|  |  | **Positivity** | Ab : 100.0 %  Ag : 90.5 % | |

14

| **References** | Todoli, F., Solano-Gallego, L., Ojeda, A., Quintana, J., Lloret, A., Roura, X., Alberola, J., Rodriguez-Cortes, A. 2009. Antileishmanial IgA in urine samples from dogs with clinical leishmaniasis. Vet. Parasitol. 159 : 17-23. | | | |
| --- | --- | --- | --- | --- |
| **Authors** | Todoli, F., Solano-Gallego, L., Ojeda, A., Quintana, J., Lloret, A., Roura, X., Alberola, J., Rodriguez-Cortes, A. | | | |
| **Year** | 2009 | **Journal** | Vet. Parasitol | |
| **Notes** |  | | | |
| **Host** | Dog | **Infection** | CVL | |
| **Country** | Spain | **Clinic** | Clinical symptoms, ELISA protein A, Bone Marrow parasitology and PCR. | |
| **Parasite** | *L. infantum* | **Biological origin** | | Urine |
| **Nb total of subjects** | | 64 | | |
| **Positive Ref N** | 64 | **Positive experiment N** | | 38 |
| **Methodologies** | Molecular | Immunology | Parasitology | Other |
| **Result** |  | ELISA antileishmania IgG and IgA : 38 |  |  |
|  |  | **Positivity** | 59.3% | |

15

| **References** | Corral, R.S., Atcheh, J.M., Freiliji, H.L. 1998. Presence of IgM antibodies to *Trypanosoma cruzi* urinary antigen in sera from patients with acute Chagas’ disease. Int. J. Parasitol. 28 : 589-594. | | | |
| --- | --- | --- | --- | --- |
| **Authors** | Corral, R.S., Atcheh, J.M., Freiliji, H.L. | | | |
| **Year** | 1998 | **Journal** | Int. J. Parasitol | |
| **Notes** |  | | | |
| **Host** | Human | **Infection** | CD acute phase | |
| **Country** | Argentina  Paraguay | **Clinic** | Parasites in blood | |
| **Parasite** | *T. cruzi* | **Biological origin** | | Urine |
| **Nb total of subjects** | | 69 (30 CD-7VL-9 Tox- 3 Malaria- 20 healthy) | | |
| **Positive Ref N** | 30 | **Positive experiment N** | | 26 |
| **Methodologies** | Molecular | Immunology | Parasitology | Other |
| **Result** |  | Ab capture with the 80 Kda Ag (ELISA) 26/30 |  |  |
|  |  | **Positivity** | 86.6% | |

16

| **References** | Castro-Sesquen, Y.E., Gilman, R.H., Mejia, R.H., Clark, D.E., Choi, J., Reimer-Mc Atee , M.J., Castro, R., Valencia-Ayala, E., Flores, J., Bowman, N., Castillo-Neyra, R., Torrico, F., Liotta, L., Bern, C., Luchini, A., The Chagas/HIV Working group in Bolivia and Peru. 2016. Use of a chagas urine nanoparticle test (Chunap) to correlate with parasitemia levels in T. cruzi/HIV co-infected patients. Plos. Negl. Trop. Dis. 10 : e0004407. | | | |
| --- | --- | --- | --- | --- |
| **Authors** | Castro-Sesquen, Y.E., Gilman, R.H., Mejia, R.H., Clark, D.E., Choi, J., Reimer-Mc Atee , M.J., Castro, R., Valencia-Ayala, E., Flores, J., Bowman, N., Castillo-Neyra, R., Torrico, F., Liotta, L., Bern, C., Luchini, A., The Chagas/HIV Working group in Bolivia and Peru. | | | |
| **Year** | 2016. | **Journal** | Plos. Negl. Trop. Dis | |
| **Notes** |  | | | |
| **Host** | Human | **Infection** | CD/HIV | |
| **Country** | Bolivia  Peru | **Clinic** | CD infection 2 positive test : Chagatest ELISA and Chagatest ELISA (Wienner Lab, Rosario-Argentina) Indirect hemaggutination test IHA (Plolychaco) | |
| **Parasite** | *T. cruzi* | **Biological origin** | | Urine |
| **Nb total of subjects** | | 55 (31 CD-24 controls) | | |
| **Positive Ref N** | 31 | **Positive experiment N** | | 23 |
| **Methodologies** | Molecular | Immunology | Parasitology | Other |
| **Result** |  | (Chunap) Ag capture on nanoparticle WB IgM anti T. cruzi LPG : 23 |  |  |
|  |  | **Positivity** | 74.1% | |

17

| **References** | Malaga-Machaca, E.S., Romero-Ramirez, A., Gilman, R.H., Astupina-Figueroa, S., Angulo, N., Lorentini, A., Lovon-Luque, C.J., Gonzo, R.A., de Carpio-Sanz, A., Cabello, I., Camargo, R., Recuenco, F., Barrueta-Soria, L.A., Verastegui, M.R., Calderon, M., Mayta, H. 2017. Plyclonal antibodies for the detection of *Trypanosoma cruzi* circulatin antigens. Plos. Negl. Trop. Dis. 11 : e0006069.. | | | |
| --- | --- | --- | --- | --- |
| **Authors** | Malaga-Machaca, E.S., Romero-Ramirez, A., Gilman, R.H., Astupina-Figueroa, S., Angulo, N., Lorentini, A., Lovon-Luque, C.J., Gonzo, R.A., de Carpio-Sanz, A., Cabello, I., Camargo, R., Recuenco, F., Barrueta-Soria, L.A., Verastegui, M.R., Calderon, M., Mayta, H. | | | |
| **Year** | 2017 | **Journal** | Plos. Negl. Trop. Dis | |
| **Notes** |  | | | |
| **Host** | Human | **Infection** | CD/HIV | |
| **Country** | Peru | **Clinic** | Positive for at least one of immunological test : ELISA, TESA blot, IHA | |
| **Parasite** | *T. cruzi* | **Biological origin** | | Urine |
| **Nb total of subjects** | | 6 | | |
| **Positive Ref N** | 6 | **Positive experiment N** | | 4 |
| **Methodologies** | Molecular | Immunology | Parasitology | Other |
| **Result** |  | Ag ELISA capture : 4 |  |  |
|  |  | **Positivity** | 66.6% | |

18

| **References** | Castro-Sesquen, Y.E., Gilman, R.H., Galdos-Cardenas, G., Ferrufino, L., Sanchez, G., Valencia-Ayala, E., Liotta, L., Bern, C., Luchini, A., the Working group on Chasa Disease in Bolivia and Peru. 2014. Use of novel chagas urine nanoparticle test (Chunap) for diagnosis of congenital Chagas Disease. Plos. Negl. Trop. Dis. 8 : e3211 | | | |
| --- | --- | --- | --- | --- |
| **Authors** | Castro-Sesquen, Y.E., Gilman, R.H., Galdos-Cardenas, G., Ferrufino, L., Sanchez, G., Valencia-Ayala, E., Liotta, L., Bern, C., Luchini, A., the Working group on Chasa Disease in Bolivia and Peru. | | | |
| **Year** | 2014 | **Journal** | Plos. Negl. Trop. Dis | |
| **Notes** |  | | | |
| **Host** | Human | **Infection** | Congenital CD | |
| **Country** | Bolivia  Peru | **Clinic** | Positive for at least one of immunological test : ELISA, TESA blot, IHA | |
| **Parasite** | *T. cruzi* | **Biological origin** | | Urine |
| **Nb total of subjects** | | 97 | | |
| **Positive Ref N** | 23 | **Positive experiment N** | | 21 |
| **Methodologies** | Molecular | Immunology | Parasitology | Other |
| **Technical** |  | (Chunap) Ag capture on nanoparticle :  21 |  |  |
|  |  | **Positivity** | 91.3 % | |

19

| **References** | Manoel Sebastião da Costa Lima Junior, Andressa Cristina Lopes Hartkopf, Rosianne A. de Souza Tsujisaki, Elisa Teruya Oshiro, Julie Teresa Shapiro, Maria de Fatima Cepa Matos, Maria Elizabeth Cavalheiros Dorval. 2018. Isolation and molecular characterization of Leishmania infantum in urine from patients with visceral leishmaniasis in Brazil. Acta Trop. 178 : 248-251. | | | |
| --- | --- | --- | --- | --- |
| **Authors** | Manoel Sebastião da Costa Lima Junior, Andressa Cristina Lopes Hartkopf, Rosianne A. de Souza Tsujisaki, Elisa Teruya Oshiro, Julie Teresa Shapiro, Maria de Fatima Cepa Matos, Maria Elizabeth Cavalheiros Dorval | | | |
| **Year** | 2018 | **Journal** | Acta Tropica | |
| **Notes** |  | | | |
| **Host** | Human | **Infection** | VL | |
| **Country** | Brazil | **Clinic** | Diagnosed VL by PCR  Parasito | |
| **Parasite** | *L. infantum* | **Biological origin** | | Urine |
| **Nb total of subjects** | | 30 | | |
| **Positive Ref N** | 30 | **Positive experiment N** | | 1 to 6 |
| **Methodologies** | Molecular | Immunology | Parasitology | Other |
| **Technical** | PCR 6 |  | 1 |  |
| **Positivity** | | | 20.0% PCR  3.3% Parasito | |

20

| **References** | Atchara Phumee, Kanyarat Kraivichian, Sarunyou Chusri, Nopadon Noppakun, Asda Vibhagool, Vivornpun Sanprasert, Vich Tampanya, Henry Wilde, and Padet Siriyasatien. 2013. Detection of Leishmania siamensis DNA in Saliva by Polymerase Chain Reaction. Am. J. Trop. Med& Hyg. 89 : 899-905. | | | |
| --- | --- | --- | --- | --- |
| **Authors** | Atchara Phumee, Kanyarat Kraivichian, Sarunyou Chusri, Nopadon Noppakun, Asda Vibhagool, Vivornpun Sanprasert, Vich Tampanya, Henry Wilde, and Padet Siriyasatien | | | |
| **Year** | 2013 | **Journal** | Am. J. Trop. Med & Hyg | |
| **Notes** |  | | | |
| **Host** | Human | **Infection** | VL | |
| **Country** | Thailand | **Clinic** | Diagnosed VL | |
| **Parasite** | *L. (Mundinia) Martiniquensis* | **Biological origin** | | Urine  Saliva |
| **Nb total of subjects** | | 6 | | |
| **Positive Ref N** | 6 | **Positive experiment N** | | PCR |
| **Methodologies** | Molecular | Immunology | Parasitology | Other |
| **Technical** | Urine : 3  Saliva : 6 |  | 1 |  |
|  |  | **Positivity** | Urine : 50.0% PCR – 16,6% Parasito  Saliva : 100.0% PCR | |

21

| **References** | Umezawa, E.S., Shikani-Yasuda, M.A., Da Silveira, J.F., Cotrim, P.C., Paranhos, G., Katzin, A.M. 1993. *Trypanosoma cruzi*: detection of a circulating antigen in urine of chagasic patients sharing common epitopes with an immunodominant repetitive antigen. Exp. Parasitol. 76 : 352-357. | | | |
| --- | --- | --- | --- | --- |
| **Authors** | Umezawa, E.S., Shikani-Yasuda, M.A., Da Silveira, J.F., Cotrim, P.C., Paranhos, G., Katzin, A.M. | | | |
| **Year** | 1993 | **Journal** | Exp. Parasitol | |
| **Notes** |  | | | |
| **Host** |  | **Infection** | CD | |
| **Country** | Brazil | **Clinic** | Clinic + IFAT + IHA | |
| **Parasite** | *T. cruzi* | **Biological origin** | | Urine |
| **Nb total of subjects** | | 90 (60 + 30 control including-healthy-VL-Schisto-Malaria) | | |
| **Positive Ref N** | 60 | **Positive experiment N** | |  |
| **Methodologies** | Molecular | Immunology | Parasitology | Other |
| **Technical** |  | Ag-urine WB with anti-H49 serum : 36/60 |  |  |
|  |  | **Positivity** | 60.0% | |

22

| **References** | Corral, R.S., Altheh, J., Alexandre, S.R., Grinstein, S., Freilij, H., Katzin, A.M. 1996. Detection and characterization of antigens in urine of patients with acute, congenital and chronic chagas disease. J. clin. Microbiol. 34 : 1957-1962. | | | |
| --- | --- | --- | --- | --- |
| **Authors** | Corral, R.S., Altheh, J., Alexandre, S.R., Grinstein, S., Freilij, H., Katzin, A.M. | | | |
| **Year** | 1996 | **Journal** | J. clin. Microbiol. | |
| **Notes** |  | | | |
| **Host** | Human | **Infection** | CD : Acute, chronic, congenital and HIV/*T. cruzi* | |
| **Country** | Argentina | **Clinic** | Hematocrit positive | |
| **Parasite** |  | **Biological origin** | | Urine |
| **Nb total of subjects** | | 17 (impossible to discern the subgroups) | | |
| **Positive Ref N** | 17 | **Positive experiment N** | | 17 |
| **Methodologies** | Molecular | Immunology | Parasitology | Other |
| **Technical** |  | Ag capture ELISA (MAB *T. cruzi* UAg) : 17 |  |  |
|  |  | **Positivity** | 100.0% | |

23

| **References** | Katzin, A.M., Marcipar, A., Freilji, H., Corral, R., Yanovsky, J.F. 1989. Rapid determination of Trypanosoma cruzi antigens in human chronic chagas disease by agglutination test. Exp. Parasitol. 68 : 208-215. | | | |
| --- | --- | --- | --- | --- |
| **Authors** | Katzin, A.M., Marcipar, A., Freilji, H., Corral, R., Yanovsky, J.F. | | | |
| **Year** | 1989 | **Journal** | Exp. Parasitol | |
| **Notes** |  | | | |
| **Host** | Human | **Infection** | CD chronic | |
| **Country** | Argentina | **Clinic** | Direct agglutination and IFI | |
| **Parasite** | *T. cruzi* | **Biological origin** | | Urine |
| **Nb total of subjects** | | 118 (58 CD, 30 healthy, 8 Schisto, 2 toxo) | | |
| **Positive Ref N** | 58 | **Positive experiment N** | | 54 |
| **Methodologies** | Molecular | Immunology | Parasitology | Other |
| **Technical** |  | DAT with horse IgG anti *T. cruzi :* 54/58 |  |  |
|  |  | **Positivity** | 85.0% | |

24

| **References** | Ben-Abid, M., Galaï, Y., Habboul, Z., Ben-Abdelaziz, R., Ben-Sghaeir,I., Aoun, K., Bouratbine, A. 2017. Diagnosis of mediterranean visceral leishmaniasis by detection of Leishmania-related antigen in urine and oral fluid samples. Acta. Trop. 167 : 72-72. | | | |
| --- | --- | --- | --- | --- |
| **Authors** | Ben-Abid, M., Galaï, Y., Habboul, Z., Ben-Abdelaziz, R., Ben-Sghaeir,I., Aoun, K., Bouratbine, A. | | | |
| **Year** | 2017 | **Journal** | Acta Tropica | |
| **Notes** |  | | | |
| **Host** | Human | **Infection** | VL | |
| **Country** | Tunisia | **Clinic** | Clinic Bone Marrow smear | |
| **Parasite** | *L. infantum* | **Biological origin** | | Urine  Oral swab |
| **Nb total of subjects** | | 97 | | |
| **Positive Ref N** | 35 | **Positive experiment N** | | 18 to 28 |
| **Methodologies** | Molecular | Immunology | Parasitology | Other |
| **Technical** |  | Katex  Urine 18/35  Oral Swab 28/35 |  |  |
|  |  | **Positivity** | 51.4 %  80.0 % | |

25

| **References** | Abeijon C, Dilo J, Tremblay JM, Viana AG, Bueno LL, Carvalho SFG, Fujiwara RT, Shoemaker CB, Campos-Neto A. Use of VHH antibodies for the development of antigen detection test for visceral leishmaniasis. Parasite Immunol. 2018 Nov;40(11):e12584. | | | |
| --- | --- | --- | --- | --- |
| **Authors** | Abeijon C, Dilo J, Tremblay JM, Viana AG, Bueno LL, Carvalho SFG, Fujiwara RT, Shoemaker CB, Campos-Neto A. | | | |
| **Year** | 2018 | **Journal** | Parasite Immunology | |
| **Notes** |  | | | |
| **Host** | Human | **Infection** | AVL | |
| **Country** | Brazil | **Clinic** | Clinic + BM aspirate PCR rapid  immunochromatographic test, IT-Leish | |
| **Parasite** | *L. infantum* | **Biological origin** | | Urine |
| **Nb total of subjects** | | 36 (12 Control) | | |
| **Positive Ref N** | 24 | **Positive experiment N** | | 10 |
| **Methodologies** | Molecular | Immunology | Parasitology | Other |
| **Technical** |  | ELISA Capture using recombinant camelid VHHs Li-isd1VHH & Lintf2-VHH  13/24 |  |  |
|  |  | **Positivity** | 41.0% | |

26

| **References** | Islam MZ, Itoh M, Shamsuzzaman SM, Mirza R, Matin F, Ahmed I, Shamsuzzaman Choudhury AK, Hossain MA, Qiu XG, Begam N, Furuya M, Leafasia JL, Hashiguchi Y, Kimura E. Diagnosis of visceral leishmaniasis by enzyme-linked immunosorbent  assay using urine samples. Clin Diagn Lab Immunol. 2002 Jul;9(4):789-94. | | | |
| --- | --- | --- | --- | --- |
| **Authors** | Islam MZ, Itoh M, Shamsuzzaman SM, Mirza R, Matin F, Ahmed I, Shamsuzzaman Choudhury AK, Hossain MA, Qiu XG, Begam N, Furuya M, Leafasia JL, Hashiguchi Y, Kimura E. | | | |
| **Year** | 2002 | **Journal** | Clin Diagn Lab Immunol | |
| **Notes** |  | | | |
| **Host** | Human | **Infection** |  | |
| **Country** | Bangladesh | **Clinic** |  | |
| **Parasite** | *L. donovani* | **Biological origin** | | Urine |
| **Nb total of subjects** | | 276 (62VL-112Healthy-59Pfalci-13 tub- 23CL-7other) | | |
| **Positive Ref N** | 62 | **Positive experiment N** | | 58 |
| **Methodologies** | Molecular | Immunology | Parasitology | Other |
| **Technical** |  | ELISA (Acetone Promsatigotes) 58 |  |  |
|  |  | **Positivity** | 93.6% | |

27

| **References** | Vogt F, Mengesha B, Asmamaw H, Mekonnen T, Fikre H, Takele Y, Adem E, Mohammed R, Ritmeijer K, Adriaensen W, Melsew Y, van Griensven J, Diro E. Antigen Detection in Urine for Noninvasive Diagnosis and Treatment Monitoring of Visceral Leishmaniasis in Human Immunodeficiency Virus Coinfected Patients: An Exploratory  Analysis from Ethiopia. Am J Trop Med Hyg. 2018 Oct;99(4):957-966. | | | |
| --- | --- | --- | --- | --- |
| **Authors** | Vogt F, Mengesha B, Asmamaw H, Mekonnen T, Fikre H, Takele Y, Adem E, Mohammed R, Ritmeijer K, Adriaensen W, Melsew Y, van Griensven J, Diro E | | | |
| **Year** | 2018 | **Journal** | Am. J. Trop. Med. Hyg | |
| **Notes** |  | | | |
| **Host** |  | **Infection** |  | |
| **Country** | Ethiopia | **Clinic** | VL-HIV (tissue aspirate +) | |
| **Parasite** |  | **Biological origin** | | Urine |
| **Nb total of subjects** | | 87 | | |
| **Positive Ref N** | 87 | **Positive experiment N** | | 77 |
| **Methodologies** | Molecular | Immunology | Parasitology | Other |
| **Technical** |  | KAtex |  |  |
|  |  | **Positivity** | 88.5% | |

28

| **References** | Sarkari B, Hatam GR, Mikaeili F, Sadeghi H, Ebrahimi S. A comparative study of antigen and antibody detection in visceral leishmaniasis using serum and urine-based ELISA. Trop Biomed. 2008 Aug;25(2):96-9. | | | |
| --- | --- | --- | --- | --- |
| **Authors** | Sarkari B, Hatam GR, Mikaeili F, Sadeghi H, Ebrahimi S. | | | |
| **Year** | 2008 | **Journal** | Trop Biomed | |
| **Notes** |  | | | |
| **Host** | Human | **Infection** | VL | |
| **Country** | Iran ? | **Clinic** | Bone marrow aspirate and IFAT | |
| **Parasite** | *L. infantum* | **Biological origin** | | Urine |
| **Nb total of subjects** | | 69 | | |
| **Positive Ref N** | 35 | **Positive experiment N** | | 21 |
| **Methodologies** | Molecular | Immunology | Parasitology | Other |
| **Technical** |  | ELISA :21 |  |  |
|  |  | **Positivity** | 60.0% | |

29

| **References** | Mirzaei A, Ahmadipour F, Cannet A, Marty P, Delaunay P, Perrin P, Dorkeld F, Sereno D, Akhoundi M. Immunodetection and molecular determination of visceral and cutaneous Leishmania infection using patients' urine. Infect Genet Evol. 2018 Sep;63:257-268. | | | |
| --- | --- | --- | --- | --- |
| **Authors** | Mirzaei A, Ahmadipour F, Cannet A, Marty P, Delaunay P, Perrin P, Dorkeld F, Sereno D, Akhoundi M | | | |
| **Year** | 2018 | **Journal** | Infection Genertic and Evolution | |
| **Notes** |  | | | |
| **Host** | Human | **Infection** | VL, CL | |
| **Country** | Iran | **Clinic** | Clinic, Parasito (Bone marrow aspirate, Smear), PCR, WB | |
| **Parasite** | *L. infantum (L.i)*  *L. major (L.m)*  *L. tropica (L.t)* | **Biological origin** | | Urine |
| **Nb total of subjects** | | 40 (14 VL + 24CL+3Healthy control) | | |
| **Positive Ref N** | 37 | **Positive experiment N** | |  |
| **Methodologies** | Molecular | Immunology | Parasitology | Other |
| **Technical** | PCR  VL (L .i) : 13  CL (L.m+L.t) : 20 | WB  VL (L .i) : 13  CL (L.m+L.t) : 22 |  |  |
|  |  | **Positivity** | PCR : 89.1%  WB : 94.5% | |

30

| **References** | Khan MG, Alam MS, Podder MP, Itoh M, Jamil KM, Haque R, Wagatsuma Y. Evaluation of rK-39 strip test using urine for diagnosis of visceral leishmaniasis in an endemic area in Bangladesh. Parasit Vectors. 2010 Nov 26;3:114. | | | |
| --- | --- | --- | --- | --- |
| **Authors** | Khan MG, Alam MS, Podder MP, Itoh M, Jamil KM, Haque R, Wagatsuma Y. | | | |
| **Notes** |  | | | |
| **Year** | 2010 | **Journal** | Parasit Vectors. | |
| **Host** | Human | **Infection** | VL | |
| **Country** | Bangladesh | **Clinic** | Clinical symptom + Serology | |
| **Parasite** | *L. donovani* | **Biological origin** | | Urine |
| **Nb total of subjects** | | 175=100 VL-25 Pf-25 Healthy (endemic)-25 Healthy (Nonendemic) | | |
| **Positive Ref N** | 100 | **Positive experiment N** | | 95 |
| **Methodologies** | Molecular | Immunology | Parasitology | Other |
| **Technical** |  | rK39 antibody : 95 |  |  |
|  |  | **Positivity** | 95.0% | |

31

| **References** | Salam MA, Khan MG, Mondal D. Urine antigen detection by latex agglutination test for diagnosis and assessment of initial cure of visceral leishmaniasis. Trans R Soc Trop Med Hyg. 2011 May;105(5):269-72. | | | |
| --- | --- | --- | --- | --- |
| **Authors** | Salam MA, Khan MG, Mondal D. | | | |
| **Year** | 2011 | **Journal** | Trans R Soc Trop Med & Hyg | |
| **Notes** |  | | | |
| **Host** | Human | **Infection** | VL | |
| **Country** | India | **Clinic** | Parasitology Clinic | |
| **Parasite** | *L. donovani* | **Biological origin** | | Urine |
| **Nb total of subjects** | | 76 | | |
| **Positive Ref N** | 36 | **Positive experiment N** | | 27 |
| **Methodologies** | Molecular | Immunology | Parasitology | Other |
| **Technical** |  | KAtex |  |  |
|  |  | **Positivity** | 75.0% | |

32

| **References** | De Colmenares M, Portus M, Riera C, Gallego M, Aisa MJ, Torras S, Munoz C. Short report: detection of 72-75-kD and 123-kD fractions of Leishmania antigen in urine of patients with visceral leishmaniasis. Am J Trop Med Hyg. 1995 May;52(5):427-8. | | | |
| --- | --- | --- | --- | --- |
| **Authors** | De Colmenares M, Portus M, Riera C, Gallego M, Aisa MJ, Torras S, Munoz C. | | | |
| **Year** | 1995 | **Journal** | Am. J. Trop. Med & Hyg | |
| **Notes** |  | | | |
| **Host** | Human | **Infection** | VL | |
| **Country** | Spain | **Clinic** | Clinic and Parasitology | |
| **Parasite** | *L. infantum* | **Biological origin** | | Urine |
| **Nb total of subjects** | | 35 | | |
| **Positive Ref N** | 15 | **Positive experiment N** | | 14 |
| **Methodologies** | Molecular | Immunology | Parasitology | Other |
| **Technical** |  | WB |  |  |
|  |  | **Positivity** | 93.3% | |

33

| **References** | Islam MZ, Itoh M, Mirza R, Ahmed I, Ekram AR, Sarder AH, Shamsuzzaman SM, Hashiguchi Y, Kimura E. Direct agglutination test with urine samples for the diagnosis of visceral leishmaniasis. Am J Trop Med Hyg. 2004 Jan;70(1):78-82. | | | |
| --- | --- | --- | --- | --- |
| **Authors** | Islam MZ, Itoh M, Mirza R, Ahmed I, Ekram AR, Sarder AH, Shamsuzzaman SM, Hashiguchi Y, Kimura E | | | |
| **Year** | 2004 | **Journal** | Am. J. Trop. Med & Hyg | |
| **Notes** |  | | | |
| **Host** | Human | **Infection** |  | |
| **Country** | Bangladesh | **Clinic** | Parasitology | |
| **Parasite** | *L. donovani* | **Biological origin** | | Urine |
| **Nb total of subjects** | | Screen with DAT 194  Screen with ELISA 300 | | |
| **Positive Ref N** | DAT 57  ELISA 75 | **Positive experiment N** | | DAT 39  ELISA 70 |
| **Methodologies** | Molecular | Immunology | Parasitology | Other |
| **Technical** |  | DAT : 29  ELISA : 70 |  |  |
|  |  | **Positivity** | DAT : 68.4%  ELISA : 93.3% | |

34

| **References** | Goswami RP, Goswami RP, Das S, Ray Y, Rahman M. Testing urine samples with rK39 strip as the simplest non-invasive field diagnosis for visceral leishmaniasis: an early report from eastern India. J Postgrad Med. 2012 Jul-Sep;58(3):180-4. | | | |
| --- | --- | --- | --- | --- |
| **Authors** | Goswami RP, Goswami RP, Das S, Ray Y, Rahman M. | | | |
| **Year** | 2012 | **Journal** | J Postgrad Med | |
| **Notes** | We consider only VL, PKDL and VL HIV patients for our meta analysis | | | |
| **Host** | Human | **Infection** | VL-VL HIV-PKDL | |
| **Country** | India | **Clinic** | Clinic and Parasitology | |
| **Parasite** | *L. donovani (inf)* | **Biological origin** | | Urine |
| **Nb total of subjects** | | 91 | | |
| **Positive Ref N** | **42 VL**  40 post traitement VL (not taken into account)  **6PKDL**  **3 HL HIV** | **Positive experiment N** | | 51 |
| **Methodologies** | Molecular | Immunology | Parasitology | Other |
| **Technical** |  | rK39 : 51 |  |  |
|  |  | **Positivity** | 100.0% | |

35

| **References** | Chakravarty J, Kumar S, Kumar R, Gautam S, Rai M, Sundar S. Evaluation of rk39 immunochromatographic test with urine for diagnosis of visceral leishmaniasis. Trans R Soc Trop Med Hyg. 2011 Sep;105(9):537-9. | | | |
| --- | --- | --- | --- | --- |
| **Authors** | Chakravarty J, Kumar S, Kumar R, Gautam S, Rai M, Sundar S. | | | |
| **Year** | 2011 | **Journal** | Trans R Soc Trop Med Hyg. | |
| **Notes** |  | | | |
| **Host** | Human | **Infection** | VL | |
| **Country** | India | **Clinic** | Parasitology | |
| **Parasite** | *L. donovani* | **Biological origin** | | Urine |
| **Nb total of subjects** | | 439 | | |
| **Positive Ref N** | 280 | **Positive experiment N** | | 270 |
| **Methodologies** | Molecular | Immunology | Parasitology | Other |
| **Technical** |  | RDT dipstick rK39 : 270 |  |  |
|  |  | **Positivity** | 96.4% | |

36

| **References** | Sundar S, Agrawal S, Pai K, Chance M, Hommel M. Detection of leishmanial antigen in the urine of patients with visceral leishmaniasis by a latex agglutination test. Am J Trop Med Hyg. 2005 Aug;73(2):269-71. | | | |
| --- | --- | --- | --- | --- |
| **Authors** | Sundar S, Agrawal S, Pai K, Chance M, Hommel M. | | | |
| **Year** | 2005 | **Journal** | Am J Trop Med Hyg | |
| **Notes** |  | | | |
| **Host** | Human | **Infection** | VL | |
| **Country** | India | **Clinic** | Parasitology | |
| **Parasite** | *L. donovani* | **Biological origin** | | Urine |
| **Nb total of subjects** | | 518 | | |
| **Positive Ref N** | 382 | **Positive experiment N** | | 333 |
| **Methodologies** | Molecular | Immunology | Parasitology | Other |
| **Technical** |  | Latex Urine : 333 |  |  |
|  |  | **Positivity** | 87.1% | |

37

| **References** | Vilaplana C, Blanco S, Domínguez J, Giménez M, Ausina V, TUral C, Muñoz C. Noninvasive method for diagnosis of visceral leishmaniasis by a latex agglutination test for detection of antigens in urine samples. J Clin Microbiol. 2004 Apr;42(4):1853-4. | | | |
| --- | --- | --- | --- | --- |
| **Authors** | Vilaplana C, Blanco S, Domínguez J, Giménez M, Ausina V, TUral C, Muñoz C. | | | |
| **Year** | 2004 | **Journal** | J Clin Microbiol | |
| **Notes** | We choose to gather results only on parasitologicaly positive patients | | | |
| **Host** | Humal | **Infection** | VL-HIV | |
| **Country** | Spain | **Clinic** | Parasitology- Clinic | |
| **Parasite** | *L. infantum* | **Biological origin** | | Urine |
| **Nb total of subjects** | | 89 | | |
| **Positive Ref N** | 16 | **Positive experiment N** | | 12 |
| **Methodologies** | Molecular | Immunology | Parasitology | Other |
| **Technical** |  | KAtex : 12 |  |  |
|  |  | **Positivity** | 75.0% | |

38

| **References** | Singh D, Pandey K, Das VN, Das S, Verma N, Ranjan A, Lal SC, Topno KR, Singh SK, Verma RB, Kumar A, Sardar AH, Purkait B, Das P. Evaluation of rK-39 strip test using urine for diagnosis of visceral leishmaniasis in an endemic region of India. Am J Trop Med Hyg. 2013 Feb;88(2):222-6. | | | |
| --- | --- | --- | --- | --- |
| **Authors** | Singh D, Pandey K, Das VN, Das S, Verma N, Ranjan A, Lal SC, Topno KR, Singh SK, Verma RB, Kumar A, Sardar AH, Purkait B, Das P | | | |
| **Year** | 2013 | **Journal** | Am J Trop Med Hyg | |
| **Notes** |  | | | |
| **Host** | Human | **Infection** | VL | |
| **Country** | India | **Clinic** | Clinic parasitology | |
| **Parasite** | *L. donovani* | **Biological origin** | | Urine |
| **Nb total of subjects** | | 786 | | |
| **Positive Ref N** | 365 | **Positive experiment N** | | 350 |
| **Methodologies** | Molecular | Immunology | Parasitology | Other |
| **Technical** |  | ICT rK39 : 350 |  |  |
|  |  | **Positivity** | 95.8% | |

39

| **References** | Diro E, Techane Y, Tefera T, Assefa Y, Kebede T, Genetu A, Kebede Y, Tesfaye A, Ergicho B, Gebre-Yohannes A, Mengistu G, Engers H, Aseffa A, Desjeux P, Boelaert M, Hailu A. Field evaluation of FD-DAT, rK39 dipstick and KATEX (urine latex agglutination) for diagnosis of visceral leishmaniasis in northwest Ethiopia. Trans R Soc Trop Med Hyg. 2007 Sep;101(9):908-14. | | | |
| --- | --- | --- | --- | --- |
| **Authors** | Diro E, Techane Y, Tefera T, Assefa Y, Kebede T, Genetu A, Kebede Y, Tesfaye A, Ergicho B, Gebre-Yohannes A, Mengistu G, Engers H, Aseffa A, Desjeux P, Boelaert M, Hailu A. | | | |
| **Year** | 2007 | **Journal** | Trans R Soc Trop Med Hyg | |
| **Notes** | Number of positive KATEX patients deduced from the given sensitivity value | | | |
| **Host** | Human | **Infection** | VL | |
| **Country** | Ethiopia | **Clinic** | Parasitology (Tissue aspirate NNN culture) | |
| **Parasite** | *L. donovani* | **Biological origin** | | Urine |
| **Nb total of subjects** | | 101 | | |
| **Positive Ref N** | 49 | **Positive experiment N** | | 38 |
| **Methodologies** | Molecular | Immunology | Parasitology | Other |
| **Technical** |  | KAtex : 38 |  |  |
|  |  | **Positivity** | 77.5% | |

40

| **References** | Motazedian M, Fakhar M, Motazedian MH, Hatam G, Mikaeili F. A urine-based polymerase chain reaction method for the diagnosis of visceral leishmaniasis in immunocompetent patients. Diagn Microbiol Infect Dis. 2008 Feb;60(2):151-4. | | | |
| --- | --- | --- | --- | --- |
| **Authors** | Motazedian M, Fakhar M, Motazedian MH, Hatam G, Mikaeili F. | | | |
| **Year** | 2008 | **Journal** | Diagn Microbiol Infect Dis. | |
| **Notes** |  | | | |
| **Host** | Human | **Infection** | VL | |
| **Country** | Iran | **Clinic** | Parasitology (smear) | |
| **Parasite** | *L. infantum* | **Biological origin** | | Urine |
| **Nb total of subjects** | | 60 | | |
| **Positive Ref N** | 30 | **Positive experiment N** | | 29 |
| **Methodologies** | Molecular | Immunology | Parasitology | Other |
| **Technical** | PCR : 29 |  |  |  |
|  |  | **Positivity** | 96.6% | |

41

| **References** | Singh DP, Goyal RK, Singh RK, Sundar S, Mohapatra TM. In search of an ideal test for diagnosis and prognosis of kala-azar. J Health Popul Nutr. 2010 Jun;28(3):281-5. | | | |
| --- | --- | --- | --- | --- |
| **Authors** | Singh DP, Goyal RK, Singh RK, Sundar S, Mohapatra TM. | | | |
| **Year** | 2010 | **Journal** | J Health Popul Nutr | |
| **Notes** |  | | | |
| **Host** | Human | **Infection** | VL | |
| **Country** | India | **Clinic** | Clinic and Parasitology | |
| **Parasite** | *L. donovani* | **Biological origin** | | Urine |
| **Nb total of subjects** | | 455 (160 Healthy from endemic country – 100 Healthy from non endemic country) | | |
| **Positive Ref N** | 150 | **Positive experiment N** | | 131 |
| **Methodologies** | Molecular | Immunology | Parasitology | Other |
| **Technical** |  | KAtex : 131 |  |  |
|  |  | **Positivity** | 87.3% | |

42

| **References** | Islam MZ, Itoh M, Takagi H, Islam AU, Ekram AR, Rahman A, Takesue A, Hashiguchi Y, Kimura E. Enzyme-linked immunosorbent assay to detect urinary antibody against recombinant rKRP42 antigen made from Leishmania donovani for the diagnosis of visceral leishmaniasis. Am J Trop Med Hyg. 2008 Oct;79(4):599-604. | | | |
| --- | --- | --- | --- | --- |
| **Authors** | Islam MZ, Itoh M, Takagi H, Islam AU, Ekram AR, Rahman A, Takesue A, Hashiguchi Y, Kimura E. | | | |
| **Year** | 2008 | **Journal** | Am J Trop Med Hyg | |
| **Notes** |  | | | |
| **Host** | Human | **Infection** | VL | |
| **Country** | Bangladesh | **Clinic** | Clinic, Parasitology, DAT, rK39 dipstick | |
| **Parasite** |  | **Biological origin** | | Urine |
| **Nb total of subjects** | | 355 | | |
| **Positive Ref N** | 115 | **Positive experiment N** | |  |
| **Methodologies** | Molecular | Immunology | Parasitology | Other |
| **Technical** |  | Anti rK42 Ab ELISA : 108 |  |  |
|  |  | **Positivity** | 93.9% | |

43

| **References** | van Griensven J, Mengesha B, Mekonnen T, Fikre H, Takele Y, Adem E, Mohammed R, Ritmeijer K, Vogt F, Adriaensen W, Diro E. Leishmania Antigenuria to Predict Initial Treatment Failure and Relapse in Visceral Leishmaniasis/HIV Coinfected Patients: An Exploratory Study Nested Within a Clinical Trial in Ethiopia. Front Cell Infect Microbiol. 2018 Mar 29;8:94. | | | |
| --- | --- | --- | --- | --- |
| **Authors** | van Griensven J, Mengesha B, Mekonnen T, Fikre H, Takele Y, Adem E, Mohammed R, Ritmeijer K, Vogt F, Adriaensen W, Diro E. | | | |
| **Year** | 2018 | **Journal** | Front Cell Infect Microbiol. | |
| **Notes** | Take only the initial KAtex results performed before treatment | | | |
| **Host** | Human | **Infection** | VL | |
| **Country** | Ethiopia | **Clinic** | Clinic and Parasitology | |
| **Parasite** | *L. donovani* | **Biological origin** | | Urine |
| **Nb total of subjects** | | 63 | | |
| **Positive Ref N** | 63 | **Positive experiment N** | | 52 |
| **Methodologies** | Molecular | Immunology | Parasitology | Other |
| **Technical** |  | KAtex : 52 |  |  |
|  |  | **Positivity** | 82.5% | |

44

| **References** | Attar ZJ, Chance ML, el-Safi S, Carney J, Azazy A, El-Hadi M, Dourado C,  Hommel M. Latex agglutination test for the detection of urinary antigens in visceral leishmaniasis. Acta Trop. 2001 Jan 15;78(1):11-6. | | | |
| --- | --- | --- | --- | --- |
| **Authors** | Attar ZJ, Chance ML, el-Safi S, Carney J, Azazy A, El-Hadi M, Dourado C,  Hommel M. | | | |
| **Year** | 2001 | **Journal** | Acta Tropica | |
| **Notes** | VL Brazil 25- Nepal 5- Yemen 29  Control  Cutaneous L eishmaniasis 12  Chagas disease 7  DD 15  Endemic control 23  Non Endemic control 312 | | | |
| **Host** | Human | **Infection** | VL | |
| **Country** | Brazil  Nepal  Yemen | **Clinic** | Parasitology | |
| **Parasite** |  | **Biological origin** | | Urine |
| **Nb total of subjects** | | 428 | | |
| **Positive Ref N** | 59 | **Positive experiment N** | | 46 |
| **Methodologies** | Molecular | Immunology | Parasitology | Other |
| **Technical** |  | Latex : 46 |  |  |
|  |  | **Positivity** | 77.9% | |

45

| **References** | Cruz I, Chicharro C, Nieto J, Bailo B, Cañavate C, Figueras MC, Alvar J.  Comparison of new diagnostic tools for management of pediatric Mediterranean visceral leishmaniasis. J Clin Microbiol. 2006 Jul;44(7):2343-7. | | | |
| --- | --- | --- | --- | --- |
| **Authors** | Cruz I, Chicharro C, Nieto J, Bailo B, Cañavate C, Figueras MC, Alvar J | | | |
| **Year** | 2006 | **Journal** | . J Clin Microbiol | |
| **Notes** | Only the 23 children from which urine are collected have been included in the table for meta analysis | | | |
| **Host** | Human (children) | **Infection** | VL | |
| **Country** | Spain | **Clinic** | Clinic, Parasitology , and PCR | |
| **Parasite** | *L. infantum* | **Biological origin** | | Urine |
| **Nb total of subjects** | | 25 | | |
| **Positive Ref N** | 25 but only 23 tested exam for urine | **Positive experiment N** | | 16 |
| **Methodologies** | Molecular | Immunology | Parasitology | Other |
| **Technical** |  | Immunoprecipitation : 16 |  |  |
|  |  | **Positivity** | 69.5% | |

46

| **References** | Vallur AC, Tutterrow YL, Mohamath R, Pattabhi S, Hailu A, Abdoun AO, Ahmed AE, Mukhtar M, Salam MA, Almeida ML, Almeida RP, Mondal D, Albertini A, Ghalib H, Duthie MS, Reed SG. Development and comparative evaluation of two antigen detection tests for Visceral Leishmaniasis. BMC Infect Dis. 2015 Sep 22;15:384. | | | |
| --- | --- | --- | --- | --- |
| **Authors** | Vallur AC, Tutterrow YL, Mohamath R, Pattabhi S, Hailu A, Abdoun AO, Ahmed AE, Mukhtar M, Salam MA, Almeida ML, Almeida RP, Mondal D, Albertini A, Ghalib H, Duthie MS, Reed SG. | | | |
| **Year** | 2015 | **Journal** | BMC Inf Dis | |
| **Notes** | Control *P. falciparum*, *M. tuberculosis*, healthy from endemic contry, healthy from non endemic country included | | | |
| **Host** | Human | **Infection** | VL | |
| **Country** | Ethiopia  Sudan  Bangladesh  Brazil | **Clinic** | Clinic signs and Parasitology | |
| **Parasite** | *L. donovani*  *L. infantum* | **Biological origin** | | Urine |
| **Nb total of subjects** | | 255 | | |
| **Positive Ref N** | Ethiopia (46)  Sudan (64)  Bangladesh (13)  Brazil (43) | **Positive experiment N** | | 166 |
| **Methodologies** | Molecular | Immunology | Parasitology | Other |
| **Technical** |  | ELISA Ag (IH) : 135  ELISA Ag ™ : 156  KAtex :101 |  |  |
|  |  | **Positivity** | ELISA Ag (IH) : 81.3%  ELISA Ag ™ : 93.9%  KAtex : 60.8% | |

47

| **References** | Rijal S, Boelaert M, Regmi S, Karki BM, Jacquet D, Singh R, Chance ML,  Chappuis F, Hommel M, Desjeux P, Van der Stuyft P, Le Ray D, Koirala S. Evaluation of a urinary antigen-based latex agglutination test in the diagnosis of kala-azar in eastern Nepal. Trop Med Int Health. 2004 Jun;9(6):724-9. | | | |
| --- | --- | --- | --- | --- |
| **Authors** | Rijal S, Boelaert M, Regmi S, Karki BM, Jacquet D, Singh R, Chance ML,  Chappuis F, Hommel M, Desjeux P, Van der Stuyft P, Le Ray D, Koirala S | | | |
| **Year** | 2004 | **Journal** | Trop Med Int Health | |
| **Notes** |  | | | |
| **Host** | Human | **Infection** | VL | |
| **Country** | Nepal | **Clinic** | Parasitology and DAT | |
| **Parasite** | *L. donovani* | **Biological origin** | | Urine |
| **Nb total of subjects** | | 232 | | |
| **Positive Ref N** | 155 | **Positive experiment N** | | 74 |
| **Methodologies** | Molecular | Immunology | Parasitology | Other |
| **Technical** |  | KAtex : 74 |  |  |
|  |  | **Positivity** | 47.7% | |

48

| **References** | Abeijon C, Singh OP, Chakravarty J, Sundar S, Campos-Neto A. Novel Antigen Detection Assay to Monitor Therapeutic Efficacy of Visceral Leishmaniasis. Am J Trop Med Hyg. 2016 Oct 5;95(4):800-802. | | | |
| --- | --- | --- | --- | --- |
| **Authors** | Abeijon C, Singh OP, Chakravarty J, Sundar S, Campos-Neto A. | | | |
| **Year** | 2016 | **Journal** | Am J Trop Med Hyg | |
| **Notes** | Not apllicable for our Meta analysis since ELISA was performed on 4 patients. | | | |
| **Host** | Human | **Infection** | VL | |
| **Country** | India | **Clinic** | Parasitology | |
| **Parasite** | *L. donovani* | **Biological origin** | | Urine |
| **Nb total of subjects** | | 7 | | |
| **Positive Ref N** | 7 | **Positive experiment N** | | 7 |
| **Methodologies** | Molecular | Immunology | Parasitology | Other |
| **Technical** |  | ELISA capture Ag Multiplex  Li-isd1  Li-trx1  Li-nft2 : 7 |  |  |
|  |  | **Positivity** | 100% | |

49

| **References** | García-García JA, Martín-Sánchez J, Gállego M, Rivero-Román A, Camacho A, Riera C, Morillas-Márquez F, Vergara S, Macías J, Pineda JA. Use of noninvasive markers to detect Leishmania infection in asymptomatic human immunodeficiency virus-infected patients. J Clin Microbiol. 2006 Dec;44(12):4455-8. | | | |
| --- | --- | --- | --- | --- |
| **Authors** | García-García JA, Martín-Sánchez J, Gállego M, Rivero-Román A, Camacho A, Riera C, Morillas-Márquez F, Vergara S, Macías J, Pineda JA | | | |
| **Year** | 2006 | **Journal** | J Clin Microbiol | |
| **Notes** | Only PCR positive patients were screened for inclusion in meta analysis | | | |
| **Host** | Human | **Infection** | VL HIV | |
| **Country** | Spain | **Clinic** | Blood PCR | |
| **Parasite** | *L. infantum* | **Biological origin** | | Urine |
| **Nb total of subjects** | | 92 | | |
| **Positive Ref N** | 28 | **Positive experiment N** | | 0 |
| **Methodologies** | Molecular | Immunology | Parasitology | Other |
| **Technical** |  | KAtex :0 |  |  |
|  |  | **Positivity** | 0.0% | |

50

| **References** | Fernández-Roldán C, Rodríguez-Grangér J, Javier Martínez R, López-Ruz MA, Navarro-Marí JM, Gutiérrez-Fernández J. [Performance of the KAtex test in screening and diagnosis for visceral leishmaniasis in a reference hospital]. Rev Esp Quimioter. 2017;30: 464–467. | | | |
| --- | --- | --- | --- | --- |
| **Authors** | Fernández-Roldán C, Rodríguez-Grangér J, Javier Martínez R, López-Ruz MA, Navarro-Marí JM, Gutiérrez-Fernández J. | | | |
| **Year** | 2017 | **Journal** | Rev Esp Quimioter | |
| **Notes** |  | | | |
| **Host** | Human | **Infection** | VL | |
| **Country** | Spain | **Clinic** | Bone Marrow parasitology | |
| **Parasite** | *L. infantum* | **Biological origin** | | Urine |
| **Nb total of subjects** | | 110 | | |
| **Positive Ref N** | 44 | **Positive experiment N** | | 22 |
| **Methodologies** | Molecular | Immunology | Parasitology | Other |
| **Technical** |  | Katex : 22 |  |  |
|  |  | **Positivity** | 50.0% | |

51

| **References** | De Almeida-Ferreira, S., Almeida, G.G., de Oliveira-Silva, S., Peixoto-Vogas, G., Toshio-Fijiwara, R., Ribeiro de Andrade, A.S., Norma-Melo, M. 2013. Nasal, Oral, and ear swabs for canine visceral leishmaniasis detection of *Leishmania infantum* DNA. Plos. Negl. Trop. Dis. 7 : e2150. | | | |
| --- | --- | --- | --- | --- |
| **Authors** | De Almeida-Ferreira, S., Almeida, G.G., de Oliveira-Silva, S., Peixoto-Vogas, G., Toshio-Fijiwara, R., Ribeiro de Andrade, A.S., Norma-Melo, M. | | | |
| **Year** | 2013. | **Journal** | Plos. Negl. Trop. Dis | |
| **Notes** | Data for oral swab the number of diagnosed dog sis reduced to 28 because saliva was preleved only on 28 diagnosed dogs. | | | |
| **Host** | Dogs | **Infection** | CanL | |
| **Country** | Brazil | **Clinic** | IFAT and ELISA positivity and or culture | |
| **Parasite** |  | **Biological origin** | | Swab  Nasal  Conjunctival  Oral  Ear |
| **Nb total of subjects** | | 72 (10 control) | | |
| **Positive Ref N** | 62 | **Positive experiment N** | | 12 to 54 |
| **Methodologies** | Molecular | Immunology | Parasitology | Other |
| **Technical** | PCR kDNA  Nasal : 54  Conjunctival : 47  Oral : 22  Ear : 12 |  |  |  |
|  |  | **Positivity** | Nasal : 87.0 %  Conjunctival : 76.9 %  Oral : 79.0 %  Ear : 43.0 % | |

52

| **References** | Pena, M.T., Naranjo, C., Klauss, G., Fondevila, D., Leiva, M., Roura, X., Davidson, M.G., Dubielzig, R.R. 2008. Histopathological features of occular Leishmaniosis in the dog. J. Comp. Pathol. 138 : 32-39. | | | |
| --- | --- | --- | --- | --- |
| **Authors** | Pena, M.T., Naranjo, C., Klauss, G., Fondevila, D., Leiva, M., Roura, X., Davidson, M.G., Dubielzig, R.R. | | | |
| **Year** | 2008 | **Journal** | Journal of comparative pathology | |
| **Notes** |  | | | |
| **Host** | Dog | **Infection** | CanL | |
| **Country** | Spain | **Clinic** | ELISA + PCR on Bone Marrow, Lymphnode or skin | |
| **Parasite** | *L. infantum* | **Biological origin** | | Occular |
| **Nb total of subjects** | | 60 | | |
| **Positive Ref N** | 60 | **Positive experiment N** | | 32 |
| **Methodologies** | Molecular | Immunology | Parasitology | Other |
| **Technical** |  |  | Histopathology 32 |  |
|  |  | **Positivity** | 53.3% | |

53

| **References** | Pilatti MM, Ferreira Sde A, de Melo MN, de Andrade AS. Comparison of PCR methods for diagnosis of canine visceral leishmaniasis in conjunctival swab samples. Res Vet Sci. 2009 Oct;87(2):255-7. | | | |
| --- | --- | --- | --- | --- |
| **Notes** |  | | | |
| **Year** | 2009 | **Journal** | Res Vet Sci | |
| **Notes** | Results of the best performing molecular methodologies are included in the Meta-analysis (Hybridation kDNA) | | | |
| **Host** | Dog | **Infection** | CVL | |
| **Country** | Brazil | **Clinic** | Clinic-ELISA-IFAT | |
| **Parasite** | *L. infantum* | **Biological origin** | | Conjunctival Swab |
| **Nb total of subjects** | | 23 | | |
| **Positive Ref N** | 23 | **Positive experiment N** | | 22 |
| **Methodologies** | Molecular | Immunology | Parasitology | Other |
| **Technical** | PCR-KDNA hybridation : 22 |  |  |  |
|  |  | **Positivity** | 95.6% | |

54

| **References** | Leite RS, Ferreira Sde A, Ituassu LT, de Melo MN, de Andrade AS. PCR diagnosis of visceral leishmaniasis in asymptomatic dogs using conjunctival swab samples. Vet Parasitol. 2010 Jun 24;170(3-4):201-6. | | | |
| --- | --- | --- | --- | --- |
| **Authors** | Leite RS, Ferreira Sde A, Ituassu LT, de Melo MN, de Andrade AS. | | | |
| **Year** | 2010 | **Journal** | Vet Parasitol | |
| **Notes** |  | | | |
| **Host** | Dog | **Infection** | CVL | |
| **Country** | Brazil | **Clinic** | Clinically negative but IFAT ELISA positive | |
| **Parasite** | *L. infantum* | **Biological origin** | | Conjunctival Swab |
| **Nb total of subjects** | | 36 | | |
| **Positive Ref N** | 30 | **Positive experiment N** | | 27 |
| **Methodologies** | Molecular | Immunology | Parasitology | Other |
| **Technical** | PCR-kDNA hybridation : 27 |  |  |  |
|  |  | **Positivity** | 90.0% | |

55

| **References** | Solano-Gallego, L., Morell, P., Arboix, M., Alberola, J., Ferrer, L. 2001. Prevalence of *Leishmania infantum* infection in dogs living in an area of canine leishmaniasis endemicity using PCR on several tissues and serology. J. Clin. Microbiol. 39 : 560-563. | | | |
| --- | --- | --- | --- | --- |
| **Authors** | Solano-Gallego, L., Morell, P., Arboix, M., Alberola, J., Ferrer, L. | | | |
| **Year** | 2001 | **Journal** | Journal of Clinical Microbiology | |
| **Notes** |  | | | |
| **Host** | Dog | **Infection** | CanL | |
| **Country** | Spain (Mallorca) | **Clinic** | ELISA, Bone Marrow aspirate | |
| **Parasite** | *L. infantum* | **Biological origin** | | Conjunctival swab |
| **Nb total of subjects** | | 100 | | |
| **Positive Ref N** | 67 | **Positive experiment N** | | 32 |
| **Methodologies** | Molecular | Immunology | Parasitology | Other |
| **Technical** | PCR : 32 | **Positivity** |  |  |
|  |  | **Positivity** | 32.0% | |

56

| **References** | Strauss-Ayali, D., Jaffe, C., Burshtain, O., Gonen, L., Baneth, G. 2004. Polymerase chain reaction using non ivasively obtained samples, for the detection of Leishmania infantum DNA in dogs. J. Infect. Dis. 189 : 1729-1733. | | | |
| --- | --- | --- | --- | --- |
| **Authors** | Strauss-Ayali, D., Jaffe, C., Burshtain, O., Gonen, L., Baneth, G. | | | |
| **Year** | 2004 | **Journal** | Journal of Infectious Disease | |
| **Notes** | We consider for metan-analysis the dogs from the group A Sero+ PCR+ Clinic | | | |
| **Host** | Dog | **Infection** | CanL & Experimental infection | |
| **Country** | Israel | **Clinic** | ELISA ,Bone Marrow and Lymph node parasitology | |
| **Parasite** |  | **Biological origin** | | Conjunctival Swab |
| **Nb total of subjects** | | 89 | | |
| **Positive Ref N** | 24 Ser+ | **Positive experiment N** | | 22 |
| **Methodologies** | Molecular | Immunology | Parasitology | Other |
| **Technical** | PCR : 22 |  |  |  |
|  |  | **Positivity** | 92.0% | |

57

| **References** | De Almeida-Ferreira, S., Ituassu, L.T., de Melo, M.N., de Andrade, A.S.R. 2008. Evaluation of the conjunctival swab for canine visceral leishmaniasis diagnosis by PCR-hybridization in Minas Gerais state, Brazil. Vet. Parasitol. 152 : 257-263. | | | |
| --- | --- | --- | --- | --- |
| **Authors** | De Almeida-Ferreira, S., Ituassu, L.T., de Melo, M.N., de Andrade, A.S.R. | | | |
| **Year** | 2008 | **Journal** | Veterinary Parasitology | |
| **Notes** | Of the 46 seropositive dogs were diveded into 2 groups for testing protocol of extraction we choose to include data of the best performing protocol. | | | |
| **Host** | Dog | **Infection** | CVL (Sympto) | |
| **Country** | Brazil | **Clinic** | Clinical sign + IFAT, ELISA and Complement reaction lysis. Symptomatic | |
| **Parasite** | *L. infantum* | **Biological origin** | | Conjunct swab |
| **Nb total of subjects** | | 66 | | |
| **Positive Ref N** | 46 (two groups 23-23) | **Positive experiment N** | | 23 |
| **Methodologies** | Molecular | Immunology | Parasitology | Other |
| **Technical** | PCR : 17  PCR dot blot kDNA : 21 |  |  |  |
|  |  | **Positivity** | PCR : 73.9 %  PCR hybridization : 91.3% | |

58

| **References** | De Almeida-Ferreira, S., Leite, R.S., Ituassu, L.T., Almeida, G.G., Souza, D.M., Fujirawa, R.T., de Andrade, A.S.R., Melo, M.N. 2012. Canine skin and conjunctival swab samples for the detection and quantification of Leishmania infantum DNA in an endemic urban area in Bazil. Plos. Negl. Trop. Dis. 6 : e1596. | | | |
| --- | --- | --- | --- | --- |
| **Authors** | De Almeida-Ferreira, S., Leite, R.S., Ituassu, L.T., Almeida, G.G., Souza, D.M., Fujirawa, R.T., de Andrade, A.S.R., Melo, M.N. | | | |
| **Year** | 2012 | **Journal** | PLos. Neglected Tropical Disease | |
| **Notes** |  | | | |
| **Host** | Dog | **Infection** | CVL | |
| **Country** | Brazil | **Clinic** | Clinic, IFAT, ELISA, BM : culture, smear, Blood PCR, and Skin : smear | |
| **Parasite** | *L. infantum* | **Biological origin** | | Conjuctival swab |
| **Nb total of subjects** | | 80 (40 clinic+, 40 clinic-) (10 control) | | |
| **Positive Ref N** | 80 | **Positive experiment N** | | 80 |
| **Methodologies** | Molecular | Immunology | Parasitology | Other |
| **Technical** | PCR, qPCR, PCR dot blot : 69 |  |  |  |
|  |  | **Positivity** | 86.2% | |

59

| **References** | Di Muccio, T., Antogoni, M.T., Fioretti, D.P., Gramiccia, M. 2012. Diagnostic value of conjunctival swab sampling associated with nested PCR for different categories of dogs naturally exposed to *Leishmania infantum* infection. J. Clin. Microbiol. 50 : 2651-2659. | | | |
| --- | --- | --- | --- | --- |
| **Authors** | Di Muccio, T., Antogoni, M.T., Fioretti, D.P., Gramiccia | | | |
| **Year** | 2012 | **Journal** | Journal of Clinical Microbiology | |
| **Notes** |  | | | |
| **Host** | Dog | **Infection** | CVL | |
| **Country** | Italia | **Clinic** | Clinic + (IFAT + parasitological test) | |
| **Parasite** | *L. infantum* | **Biological origin** | | Conjunctival swab |
| **Nb total of subjects** | | 273 | | |
| **Positive Ref N** | 72 | **Positive experiment N** | | 55 |
| **Methodologies** | Molecular | Immunology | Parasitology | Other |
| **Technical** | Nested PCR : 55 |  |  |  |
|  |  | **Positivity** | 76.3% | |

60

| **References** | Geisweid, K., Weber, K., Sauter-Louis, C., Hartmann, K. 2013. Evaluation of a conjunctival swab polymerase chain reaction for the detection of Leishmania infantum in dogs in a non-endemic area. Vet. J. 198 : 187-192. | | | |
| --- | --- | --- | --- | --- |
| **Authors** | Geisweid, K., Weber, K., Sauter-Louis, C., Hartmann, K. | | | |
| **Year** | 2013 | **Journal** | The Veterinary Journal | |
| **Notes** |  | | | |
| **Host** | Dog | **Infection** | CVL | |
| **Country** | Deutshland | **Clinic** | Clinic + Bone Marrow, Lymph Node, and blood PCR | |
| **Parasite** | *L. infantun* | **Biological origin** | | Conjunctival swab |
| **Nb total of subjects** | | 74 | | |
| **Positive Ref N** | 43 | **Positive experiment N** | | 39 |
| **Methodologies** | Molecular | Immunology | Parasitology | Other |
| **Technical** | PCR (kDNA) :  39 |  |  |  |
|  |  | **Positivity** | 90.6 % | |

61

| **References** | Ferreira, A.L.C., Carregal, V.M., de Almeida-Ferreira, S., Leite, R.S., de Andrade, A.S.R. 2014. Detection of *Leishmania infantum* in 4 different dog samples by real-time PCR and ITS1 nested PCR. 2014. Diag. Microbiol. Inf. Dis. 78 : 418-421. | | | |
| --- | --- | --- | --- | --- |
| **Authors** | Ferreira, A.L.C., Carregal, V.M., de Almeida-Ferreira, S., Leite, R.S., de Andrade, A.S.R. | | | |
| **Year** | 2014 | **Journal** | Diagnostic Microbiology and Infectious Disease. | |
| **Notes** | Only the best performing protocol was included in the meta analysis | | | |
| **Host** | Dog | **Infection** | CanL | |
| **Country** | Brazil | **Clinic** | Clinic, ELISA, IFAT, Parasitology | |
| **Parasite** | *L. infantum* | **Biological origin** | | Conjunctival swab |
| **Nb total of subjects** | | 60 | | |
| **Positive Ref N** | 60 | **Positive experiment N** | | 54 to 59 |
| **Methodologies** | Molecular | Immunology | Parasitology | Other |
| **Technical** | **qPCR**  CS : 59  **nPCR**  CS : 54 |  |  |  |
|  |  | **Positivity** | **qPCR**  CS : 98.0 %  **nPCR**  CS : 90.0 % | |

62

| **References** | Ceccarelli, M., Galluzzi, L., Sisti, D., Bianchi, B., Magnani, M. 2014. Application of qPCR in conjuctival swab samples for the evaluation of canine leishmaniasis in borderline cases or disease relapse and correlation with clinical parameter. Parasites & Vectors. 7 : 460 | | | |
| --- | --- | --- | --- | --- |
| **Authors** | Ceccarelli, M., Galluzzi, L., Sisti, D., Bianchi, B., Magnani, M. | | | |
| **Year** | 2014 | **Journal** | Parasites & Vectors | |
| **Notes** |  | | | |
| **Host** | Dog | **Infection** | CanL | |
| **Country** | Italia | **Clinic** | Clinic, IFAT, PCR | |
| **Parasite** | *L. infantum* | **Biological origin** | | Conjunctival swab |
| **Nb total of subjects** | | 80 | | |
| **Positive Ref N** | 56 | **Positive experiment N** | | 26 |
| **Methodologies** | Molecular | Immunology | Parasitology | Other |
| **Technical** | qPCR : 24 |  |  |  |
|  |  | **Positivity** | 46.4% | |

63

| **References** | Gao, C.H., Ding, D., Wang, J.Y., Steverding, D., Wang, X., Yang, Y.T., Shi, F. 2015. Development of a LAMP assay for detection of Leishmania infantum infection in dogs using conjunctival swab samples. Parasites & Vectors. 8 : 370 | | | |
| --- | --- | --- | --- | --- |
| **Authors** | Gao, C.H., Ding, D., Wang, J.Y., Steverding, D., Wang, X., Yang, Y.T., Shi, F. | | | |
| **Year** | 2015 | **Journal** | Parasites & Vectors | |
| **Notes** |  | | | |
| **Host** | Dogs | **Infection** | CanL | |
| **Country** | China | **Clinic** | Clinic, Bone Marrow PCR and Smear, ELISA | |
| **Parasite** | *L. infantum* | **Biological origin** | | Conjunctival swab |
| **Nb total of subjects** | | 112 (33 non endemic) | | |
| **Positive Ref N** | 5 | **Positive experiment N** | | 5 |
| **Methodologies** | Molecular | Immunology | Parasitology | Other |
| **Technical** | LAMP 5 |  |  |  |
|  |  | **Positivity** | 100.0% | |

64

| **References** | Lombardo, G., Pennisi, M.G., Lupo, T., Migliazzo, A., Capri, A., Solano-Gallego, L. 2012. Detection of Leishmania infantum DNA by real-time PCR in canine oral and conjunctival swabs and comparison with other diagnostic. Vet. Parasitol. 184 : 10-17. | | | |
| --- | --- | --- | --- | --- |
| **Authors** | Lombardo, G., Pennisi, M.G., Lupo, T., Migliazzo, A., Capri, A., Solano-Gallego, L | | | |
| **Year** | 2012 | **Journal** | Veterinary Parasitology | |
| **Notes** |  | | | |
| **Host** | Dog | **Infection** | CVL | |
| **Country** | Italia | **Clinic** | Clinic, Blood and Lymph node PCR | |
| **Parasite** | *L. infantum* | **Biological origin** | | Conjunctival swab  Oral swab |
| **Nb total of subjects** | | 163 | | |
| **Positive Ref N** | 57 | **Positive experiment N** | | 10 to 21 |
| **Methodologies** | Molecular | Immunology | Parasitology | Other |
| **Technical** | qPCR  OS : 10  CS : 21 |  |  |  |
|  |  | **Positivity** | OS : 17.5%  CS : 36.8% | |

65

| **References** | Benassi, J.C., Benvenga, G.U., Ferreira, H.L., Perreira, V.F., Keid, L.B., Soares, R., Ferreira, de Sousa Oliveira, T.M.F. 2017. Detection of Leishmania infantum DNA in conjuctival swabs of cats by quantitative real time PCR. Exp. Parasitol. 177 : 93-97. | | | |
| --- | --- | --- | --- | --- |
| **Authors** | Benassi, J.C., Benvenga, G.U., Ferreira, H.L., Perreira, V.F., Keid, L.B., Soares, R., Ferreira, de Sousa Oliveira, T.M.F. | | | |
| **Year** | 2017 | **Journal** | Experimental Parasitology | |
| **Notes** |  | | | |
| **Host** | Cat | **Infection** | Cat L | |
| **Country** | Brazil | **Clinic** | Clinic + PCR | |
| **Parasite** | NK | **Biological origin** | | Conjunctival swab |
| **Nb total of subjects** | | 108 | | |
| **Positive Ref N** | 2 | **Positive experiment N** | | 2 |
| **Methodologies** | Molecular | Immunology | Parasitology | Other |
| **Technical** | PCR : 2  qPCR : 2 |  |  |  |
|  |  | **Positivity** | 100.0% | |

66

| **References** | Pereira, V.F., Benassi, J.C., Starke-Buzetti, W.A., Silva, D.T., Ferreira, H.L., Keid, L.B., Soares, R.M., de Azevedo Ruiz, V.L., de Souza Oliveira, T.M.F. 2016. Detection of canine visceral leishmaniasis by conjunctival swab PCR. Rev. Soc. Bras. Med. Trop. 49 : 104-106. | | | |
| --- | --- | --- | --- | --- |
| **Authors** | Pereira, V.F., Benassi, J.C., Starke-Buzetti, W.A., Silva, D.T., Ferreira, H.L., Keid, L.B., Soares, R.M., de Azevedo Ruiz, V.L., de Souza Oliveira, T.M.F. | | | |
| **Year** | 2016 | **Journal** | Revista da Societa Brasilera de Medicina Tropical | |
| **Notes** |  | | | |
| **Host** | Dog | **Infection** | CanL | |
| **Country** | Brazil | **Clinic** | Clinic, IFAT (<1 :40), Blood PCR | |
| **Parasite** | *L. infatnum* | **Biological origin** | | Conjunctival swab |
| **Nb total of subjects** | | 213 | | |
| **Positive Ref N** | 60 | **Positive experiment N** | | 28 |
| **Methodologies** | Molecular | Immunology | Parasitology | Other |
| **Technical** | qPCR : 28  PCR : 28 |  |  |  |
|  |  | **Positivity** | 46.0% | |

67

| **References** | Aschar, M., de Oliveria, E.T.B., Laurenti, M.D., Marcondes, M., Tolezano, J.E., Hiramoto, R.M., Corbett, C.E.P., da Matta, V.L.R. 2016. Value of the oral swab for the molecular diagnosis of dogs in different stages of infection with Leishmania infantum. 225. Vet. Parasitol. 108-113. | | | |
| --- | --- | --- | --- | --- |
| **Authors** | Aschar, M., de Oliveria, E.T.B., Laurenti, M.D., Marcondes, M., Tolezano, J.E., Hiramoto, R.M., Corbett, C.E.P., da Matta, V.L.R. | | | |
| **Year** | 2016. | **Journal** | Veterinary Parasitology | |
| **Notes** |  | | | |
| **Host** | Dog | **Infection** | CanL | |
| **Country** | Brazil | **Clinic** | Clinic, Blood and Lymph Node PCR, DPP (CVL rapid test) EIE (Enzyme-immunoassay) Bio manguinhos | |
| **Parasite** | *L. infantum* | **Biological origin** | | Conjunctival swab  Oral swab |
| **Nb total of subjects** | |  | | |
| **Positive Ref N** | 92 | **Positive experiment N** | | 62 to 63 |
| **Methodologies** | Molecular | Immunology | Parasitology | Other |
| **Technical** | qPCR  Oral : 62  Conj : 63 |  |  |  |
|  |  | **Positivity** | Oral : 67.4 %  Conj : 68.5% | |

68

| **References** | De Sousa-Gonçalves, R., Franke, C.R., Magalhaes-Junior, J.T., Souza, B.M.P.S., Solca, M.S ;, Larangeira, D.F., Barrouin-Melo, S.M. 2016. Association between Leishmania infantum DNA in the hair of dogs and their infectiousness to *Lutzomyia longipalpis*. 2016. Vet. Parasitol. 232 : 43-47. | | | |
| --- | --- | --- | --- | --- |
| **Authors** | De Sousa-Gonçalves, R., Franke, C.R., Magalhaes-Junior, J.T., Souza, B.M.P.S., Solca, M.S ;, Larangeira, D.F., Barrouin-Melo, S.M. | | | |
| **Year** | 2016. | **Journal** | Veterinary Parasitol | |
| **Notes** |  | | | |
| **Host** | Dog | **Infection** | CVL | |
| **Country** | Brazil | **Clinic** | Clinic, ELISA, Culture, Splenic aspirate PCR, Xeno diagnosis | |
| **Parasite** | *L. infantum* | **Biological origin** | | Hair  keratinocyte |
| **Nb total of subjects** | | 23 | | |
| **Positive Ref N** | 15 | **Positive experiment N** | | 12 |
| **Methodologies** | Molecular | Immunology | Parasitology | Other |
| **Technical** | qPCR 12 |  |  |  |
|  |  | **Positivity** | 80.0% | |

69

| **References** | Belinchon-Lorenzo, S., Iniesta, V., Parejo, J.C., Fernandez-Cotrina, J., Munoz-Madrid, R., Soto, M., Alonso, C., Nieto, L.C.G. 2013. Detecction of Leishmania infantum kinetoplast minicercle DNA by real time PCR in hair of dogs with leishmaniosis. Vet. Parasitol. 192 : 43-50. | | | |
| --- | --- | --- | --- | --- |
| **Authors** | Belinchon-Lorenzo, S., Iniesta, V., Parejo, J.C., Fernandez-Cotrina, J., Munoz-Madrid, R., Soto, M., Alonso, C., Nieto, L.C.G. | | | |
| **Year** | 2013 | **Journal** | Veterinary Parasitology | |
| **Notes** |  | | | |
| **Host** | Dog | **Infection** | CanL | |
| **Country** | Spain | **Clinic** | Clinic, ELISA, IFAT, Blood PCR, | |
| **Parasite** | *L. infantum* | **Biological origin** | | Hair  Keratinocyte |
| **Nb total of subjects** | | 28 | | |
| **Positive Ref N** | 13 | **Positive experiment N** | | 10 |
| **Methodologies** | Molecular | Immunology | Parasitology | Other |
| **Technical** | qPCR : 10 |  |  |  |
|  |  | **Positivity** | 76.7 % | |

70

| **References** | Ortega, M.V., Moreno, I., Dominguez, M., de la Cruz, M.L., Martin, A.B., Rodriguez-Bertos, A., Lopez, R., Navarro, A., Gonzalez, S., Mazaiegos, M., Goyache, J., Dominguez, L., Garcia, N. 2017. Application of a specific quantitative real-time (qPCR) to identify Leishmania *infantum* DNA in spleen, skin and hair samples of wild leproridae. Vet. Parasitol. 243 : 92-99. | | | |
| --- | --- | --- | --- | --- |
| **Authors** | Ortega, M.V., Moreno, I., Dominguez, M., de la Cruz, M.L., Martin, A.B., Rodriguez-Bertos, A., Lopez, R., Navarro, A., Gonzalez, S., Mazaiegos, M., Goyache, J., Dominguez, L., Garcia, N. | | | |
| **Year** | 2017 | **Journal** | Veterinary Parasitology | |
| **Notes** | Data of wild leporidae were agregated to gain a global sensitivity | | | |
| **Host** | Rabbit  *Oryctolagus cuniculus*  *Lepus granatensis* | **Infection** | Natural | |
| **Country** | Spain | **Clinic** | IFAT, (<1 :50), Spleen and Blood PCR and culture | |
| **Parasite** | *L. infantum* | **Biological origin** | | Hair |
| **Nb total of subjects** | | 294 | | |
| **Positive Ref N** | 65 (33 O. cun + 32 L. gran) | **Positive experiment N** | | 17 to 22 |
| **Methodologies** | Molecular | Immunology | Parasitology | Other |
| **Technical** | qPCR an nPCR  22/33 *O. cun*  17/32 *L. gra* |  |  |  |
|  |  | **Positivity** | 66.3% O. cun  32.6% L. gra | |

71

| **References** | Vaish, M., Mehrotra, S., Chakravarty, J., Sundar, S. 2011. Nonivasive molecular diagnosis of human visceral leishmaniasis. J. Clin. Microbiol. 49 : 2003-2005. | | | |
| --- | --- | --- | --- | --- |
| **Authors** | Vaish, M., Mehrotra, S., Chakravarty, J., Sundar, S. | | | |
| **Year** | 2011 | **Journal** | Journal of Clinical Microbiology | |
| **Notes** |  | | | |
| **Host** | Human | **Infection** | VL-Kala azar | |
| **Country** | India | **Clinic** | Parasitology | |
| **Parasite** | *L. donovani* | **Biological origin** | | Oral Swab |
| **Nb total of subjects** | | 307 | | |
| **Positive Ref N** | 148 | **Positive experiment N** | | 123 |
| **Methodologies** | Molecular | Immunology | Parasitology | Other |
| **Technical** | PCR  123 |  |  |  |
|  |  | **Positivity** | 83.1% | |

72

| **References** | Pinho, R.T., Pedrosa, R.C., Costa-Martins, P., Castello-Branco, L.R.R. 1999. Saliva ELISA : a method for the diagnosis of chronic chagas disease in endemic areas. Acta. Trop. 72 : 31-38. | | | |
| --- | --- | --- | --- | --- |
| **Authors** | Pinho, R.T., Pedrosa, R.C., Costa-Martins, P., Castello-Branco, L.R.R. | | | |
| **Year** | 1999 | **Journal** | Acta Tropica | |
| **Notes** |  | | | |
| **Host** | Human | **Infection** | CD | |
| **Country** | Brazil | **Clinic** | Clinic, ELISA, Hemagglutination, IFAT, Cardiac dysfunction | |
| **Parasite** | *T. cruzi* | **Biological origin** | | Saliva |
| **Nb total of subjects** | | 214 | | |
| **Positive Ref N** | 114 | **Positive experiment N** | | 103 |
| **Methodologies** | Molecular | Immunology | Parasitology | Other |
| **Technical** |  | ELISA 103 |  |  |
|  |  | **Positivity** | 90.3% | |

73

| **References** | Pandey, N., Siripattanapipong, S., Leelayoova, S., Manomat, J., Mungthin, M., Tan-ariya, P., Bualert, L., Naaglor, T., Siriyasatien, P., Piyaraj, P. 2018. Detection of Leishmania DNA in saliva among patients with HIV/AIDS in Trang Province, southern Thailand. Acta. Trop. 185: 294-300 | | | |
| --- | --- | --- | --- | --- |
| **Authors** | Pandey, N., Siripattanapipong, S., Leelayoova, S., Manomat, J., Mungthin, M., Tan-ariya, P., Bualert, L., Naaglor, T., Siriyasatien, P., Piyaraj, P. | | | |
| **Year** | 2018 | **Journal** | Acta Tropica | |
| **Notes** |  | | | |
| **Host** | Human | **Infection** | HIV-Leish | |
| **Country** | Thailand | **Clinic** | Buffy coat PCR, ELISA | |
| **Parasite** | *Leishmania sp* | **Biological origin** | | Saliva |
| **Nb total of subjects** | | 307 | | |
| **Positive Ref N** | 130 | **Positive experiment N** | | 46 |
| **Methodologies** | Molecular | Immunology | Parasitology | Other |
| **Technical** | nPCR  46 |  |  |  |
|  |  | **Positivity** | 35.8% | |

74

| **References** | Boni S.M., Oyafuso, L.K, de Cassia Soler, R., Lindoso, J.A.L. 2017. Efficiency of noninvasive sampling methods (swab) together with Polymerase Chain Reaction (PCR) for diagnosing American Tegumentary Leishmaniasis. Rev. Inst. Med. Trop. Sao Paulo. 59 : e38. | | | |
| --- | --- | --- | --- | --- |
| **Authors** | Boni S.M., Oyafuso, L.K, de Cassia Soler, R., Lindoso, J.A.L. | | | |
| **Year** | 2017 | **Journal** | Revistat de Instituto de Medicina Tropical Sao Paulo | |
| **Notes** |  | | | |
| **Host** | Human | **Infection** | ATL | |
| **Country** | Brazil | **Clinic** | Lesions, Biopsy, Culture, PCR | |
| **Parasite** | L. (Viannia) sp. | **Biological origin** | | Nasal swab |
| **Nb total of subjects** | | 25 | | |
| **Positive Ref N** | 15 (CL+MCL) | **Positive experiment N** | | 15 |
| **Methodologies** | Molecular | Immunology | Parasitology | Other |
| **Technical** | PCR, qPCR  15 |  |  |  |
|  |  | **Positivity** | 100.0% | |

75

| **References** | Lejon V, Kwete J, Büscher P. Towards saliva-based screening for sleeping sickness? Trop Med Int Health. 2003 Jul;8(7):585-8. | | | |
| --- | --- | --- | --- | --- |
| **Authors** | Lejon V, Kwete J, Büscher P. | | | |
| **Year** | 2003 | **Journal** | Trop Med & Int Health | |
| **Notes** |  | | | |
| **Host** | Human | **Infection** | Sleeping sickness | |
| **Country** | DRC | **Clinic** | Diagnosis | |
| **Parasite** | *T . b. gambiense* | **Biological origin** | | Saliva |
| **Nb total of subjects** | | 47 | | |
| **Positive Ref N** | 23 | **Positive experiment N** | | 23 |
| **Methodologies** | Molecular | Immunology | Parasitology | Other |
| **Technical** |  | ELISA Ab : 23 |  |  |
|  |  | **Positivity** | 100.0% | |

76

| **References** | Lejon V, Jamonneau V, Solano P, Atchade P, Mumba D, Nkoy N, Bébronne N, Kibonja T, Balharbi F, Wierckx A, Boelaert M, Büscher P. Detection of trypanosome-specific antibodies in saliva, towards non-invasive serological diagnosis of sleeping sickness. Trop Med Int Health. 2006 May;11(5):620-7. | | | |
| --- | --- | --- | --- | --- |
| **Authors** | Lejon V, Jamonneau V, Solano P, Atchade P, Mumba D, Nkoy N, Bébronne N, Kibonja T, Balharbi F, Wierckx A, Boelaert M, Büscher P | | | |
| **Year** | 2006 | **Journal** | Trop Med & Int Health | |
| **Notes** |  | | | |
| **Host** | Human | **Infection** | Sleeping sickness | |
| **Country** | DRC  Benin | **Clinic** |  | |
| **Parasite** | *T.b. gambiense* | **Biological origin** | | Saliva |
| **Nb total of subjects** | | 206 | | |
| **Positive Ref N** | 78 | **Positive experiment N** | | 74 |
| **Methodologies** | Molecular | Immunology | Parasitology | Other |
| **Technical** |  | CATT  & ELISA Ab |  |  |
|  |  | **Positivity** | 94.8% | |

77

| **References** | Belinchon-Lorenzo, S., Pajero, J.C., Iniesta, V., Fernandez-Cotrina, J., Munoz-Madrid, R., Monroy, I., Baz, V., Gomez-Luque, A., Serrano-Aguilera, F.J., Berneto, J.L. Gomez-Nieto, L.C. 2016. First detection of Leishmania kDNA in canine cerumen samples by qPCR. Vet parasitol. 228 : 65-68. | | | |
| --- | --- | --- | --- | --- |
| **Authors** | Belinchon-Lorenzo, S., Pajero, J.C., Iniesta, V., Fernandez-Cotrina, J., Munoz-Madrid, R., Monroy, I., Baz, V., Gomez-Luque, A., Serrano-Aguilera, F.J., Berneto, J.L. Gomez-Nieto, L.C. | | | |
| **Year** | 2016 | **Journal** | Vet parasitol | |
| **Notes** |  | | | |
| **Host** | Dog | **Infection** | CVL | |
| **Country** | Spain | **Clinic** | IFAT and SLA ELISA positivity | |
| **Parasite** | *L. infantum* | **Biological origin** | | Ear/Cerumen |
| **Nb total of subjects** | | 38 | | |
| **Positive Ref N** | 33 | **Positive experiment N** | | 30 |
| **Methodologies** | Molecular | Immunology | Parasitology | Other |
| **Technical** | kDNA qPCR  30 |  |  |  |
|  |  | **Positivity** | 90.9% | |
